# The Role of Shape Commensurability in Chirality Transfer: Gold Nanoshape Solutes in a Discotic Nematic Liquid Crystal Solvent

**DOI:** 10.1002/anie.1907246

**Published:** 2026-05-14

**Authors:** Gourab Acharjee, Lara Querciagrossa, Grace A. R. Rohaley, Nicholas M. Kamuti, Ashwathanarayana Gowda, Suraj Kumar Pathak, Asmita Shah, Kun Zhang, Jianfang Wang, Claudio Zannoni, Torsten Hegmann

**Affiliations:** ^1^ Department of Chemistry and Biochemistry Kent State University Kent Ohio USA; ^2^ Cineca HPC Bologna Italy; ^3^ Advanced Materials and Liquid Crystal Institute Materials Science Graduate Program Kent State University Kent Ohio USA; ^4^ Department of Physics The Chinese University of Hong Kong Shatin Hong Kong SAR China; ^5^ Dipartimento Di Chimica Industriale and INSTM Università Di Bologna Bologna Italy; ^6^ Brain Health Research Institute Kent State University Kent Ohio USA

**Keywords:** chiral discotic nematic, chirality, chirality index, helical twisting power, shape analysis

## Abstract

Chirality, as an inherently geometric concept, is well understood at most length scales and is a principal attribute of objects and figures. Quantitative models predicting the efficacy of chirality transmission across length scales have only recently begun to emerge. We provide further proof‐of‐concept data and calculations for a modus operandi for nanoshape solutes featuring a chiral ligand shell in an achiral discotic nematic (ND) liquid crystal solvent, demonstrating that chirality transfer can be understood through remarkably simple geometric considerations. This mechanism is based on the product of a pseudoscalar chirality indicator and a geometric shape compatibility factor based on the 2D isoperimetric quotients for nanoshape solutes and N_D_ molecule. The model is tested on an experimental set of precisely engineered gold nanoshapes, rods, prisms, and discs, that validates that shape commensurability between nanoscale solute and nematic solvent is a prerequisite for efficacious chirality transfer as determined by the helical twisting power of the nanoshapes in the induced chiral N_D_* phase. Thus, we predict that libraries of calculated and in‐parallel acquired experimental data among related nanoshapes and even small organic molecules pave the way for predictive calculations of chirality transfer in nanoscale, macromolecular, biological, and small‐molecule systems.

## Graphical abstract

We report on a suitable approach to predict the chirality “strength” and efficacy of chirality transfer from chiral nanoshape solutes to an achiral discotic nematic (N_D_) liquid crystal solvent. Highly efficacious chirality transfer based on shape commensurability between nanoshape solute (in the form of gold nanodiscs, GNDs) and a N_D_ solvent was experimentally confirmed and *a priori* predicted by an independently calculated, solute–solvent shape compatibility factor‐corrected pseudoscalar chirality indicator.

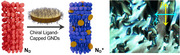

## 1 Introduction

Methodologies to quantify the chirality of objects and molecules by establishing a degree of chirality that equals zero only if the object is achiral fall into distinct categories that have continued to be of great interest over many decades [1, 2, 3]. Accordingly, measures of a degree of chirality [4] are commonly defined in a relative or absolute way. The relative approach quantifies chirality either by (i) the extent to which a chiral object differs from its achiral counterpart or (ii) the extent to which two enantiomorphic shapes differ from one another. Examples of the first type include Guye's geometric chirality product [5] or the distortions of a chemical structure from a more symmetrical reference structure as introduced by Dunitz et al. [6, 7, 8]. A classic example of this methodology is the Hausdorff chirality measure [9, 10, 11, 12] that determines the distances between sets of points representing the two specular images of the enantiomorphs (the Hausdorff distances). Other chirality measures based on more rigid body shapes and a corresponding shape analysis include Kuz'min's [13] and Mezey's methods [14], calculating either a dissymmetry function based on the geometry of the molecule or by identifying the minimal resolution needed to fill the interior of Jordan curves representing the object in 2D with geometrically identical 2‐D shapes, such as squares, respectively. A commonality among these methods is that the degree of chirality of the underlying geometric figures or objects is measured by their shape and remains invariant with changes in size [1]. Notice also that the relative approaches measure a magnitude of chirality but not its sign.

In contrast, there also exists an absolute (as opposed to being relative to any reference) pseudoscalar chirality indicator that is derived purely from a molecule's or object's geometry [15, 16, 17, 18], termed the average or maximum chirality index $\left\langle G_{oa}^{a}\right\rangle$ (Equation 1) or $|G_{oa,max}^{a}|$, starting from the expression:

(1) $$\begin{array}{ccc} & & G_{\mathrm{oa}}^{a}=\sum_{\mathrm{all}\;\mathrm{perm}\mathrm{utat}\mathrm{ion}\;\mathrm{of}i,j,k,l}\\ & & \left\{\begin{array}{cc} \frac{1}{N}\frac{\left[\left(r_{\mathrm{ij}}\times r_{\mathrm{kl}}\right)\cdot r_{\mathrm{il}}\right]\left(r_{\mathrm{ij}}\cdot r_{\mathrm{jk}}\right)\left(r_{\mathrm{jk}}\cdot r_{\mathrm{kl}}\right)}{{\left(r_{\mathrm{ij}}r_{\mathrm{jk}}r_{\mathrm{kl}}\right)}^{2}r_{\mathrm{il}}} & \mathrm{if}\;i<j<k<l\in [1,n]\\ & 0\;\mathrm{otherwise} \end{array}\right. \end{array}$$

in which *N* is the total number of atoms (or coarse‐grain beads) considered, and **r**_*ij*_ is the vector distance between any two atoms *i* and *j* (subscript *oa* stands for overall). Notice that $G_{oa}^{a}$ is scale (e.g., dilation) invariant but changes sign with handedness. The utility of $\left\langle G_{oa}^{a}\right\rangle$ was previously validated for small molecules [19], proteins [17], and erodium awns [20]. Most notably, however, $\left\langle G_{oa}^{a}\right\rangle$ was also calculated for sets of chiral ligand monolayer‐capped gold nanoparticles varying in shape and size that were in parallel studied experimentally regarding their efficacy of inducing a chiral nematic liquid crystal (N*‐LC) phase in an achiral nematic liquid crystal (N‐LC) solvent [16, 21]. $\left\langle G_{oa}^{a}\right\rangle$, as we have shown for gold nanoshapes, depends solely on geometric information, that is, the position and orientation of the chiral ligands (or, more precisely, their coarse‐grained (CG) representations) with respect to the nanoparticle frames, and thus indirectly on the shapes and sizes of the nanoparticles. These studies were among the first to address what Mislow indicated as: “the daunting challenge of bridging the gap between the results of chiral shape analysis and the world of experimental observables” [1].

As such an observable, our earlier work established a direct correlation between $|G_{oa,max}^{a}|$ and the molar helical twisting power (β_*mol*_) derived from the experimentally measured helical pitch, *p*, of the chiral nematic liquid crystal (N*‐LC) phases induced by the chiral ligand‐capped gold nanoparticles with either quasi‐spherical (polyhedral) or rod‐like shape: β_*mol*_ = 1/*p* · *x_Ligand_*  · *r*, in which *p* is the helical pitch, *x_Ligand_* the mole fraction of the chiral ligand, and *r* the enantiomeric purity of the well‐dispersed nanoshapes. More specifically, we assumed:

(2) $$\beta_{mol}\propto \left|G_{oa,max}^{a}\right|$$

where $|G_{oa,max}^{a}|$ is the absolute maximum of the average $\left\langle G_{oa}^{a}\right\rangle$ values obtained for the various ligand orientations on these gold nanoshapes [16]. The use of $|G_{oa,max}^{a}|$ is supported by the fact that the β_*mol*_ data, calculated from the pitch, *p*, of the induced N*‐LC phases, are known to be especially sensitive to the highest rather than average chirality values. Extending our study to nanoparticle solutes increasingly deviating from the two initially examined basic shapes (i.e., quasi‐spherical polyhedra and rods), we found it important to introduce a shape compatibility factor, *S^XN^*, serving as a transmission coefficient of the chiral solute *X* to the achiral N‐LC molecules *N*, so that:

(3) $$\beta_{mol}\propto \left|G_{oa,max}^{a}S^{XN}\right|$$

Considering the stacking between the solute and the nematic that we imagine at the origin of the pitch formation, *S^XN^* is in turn taken to be related to the inverse difference between the 2‐D isoperimetric ratios (*IPR*_2D_) of solute *X* and solvent *N* (Equation 4): [22, 23]

(4) $$S^{XN}=\frac{1}{\Delta IPR_{2D}^{XN}}=\frac{1}{\left|IPR_{2D}^{X}-IPR_{2D}^{N}\right|}$$

**FIGURE 1:**
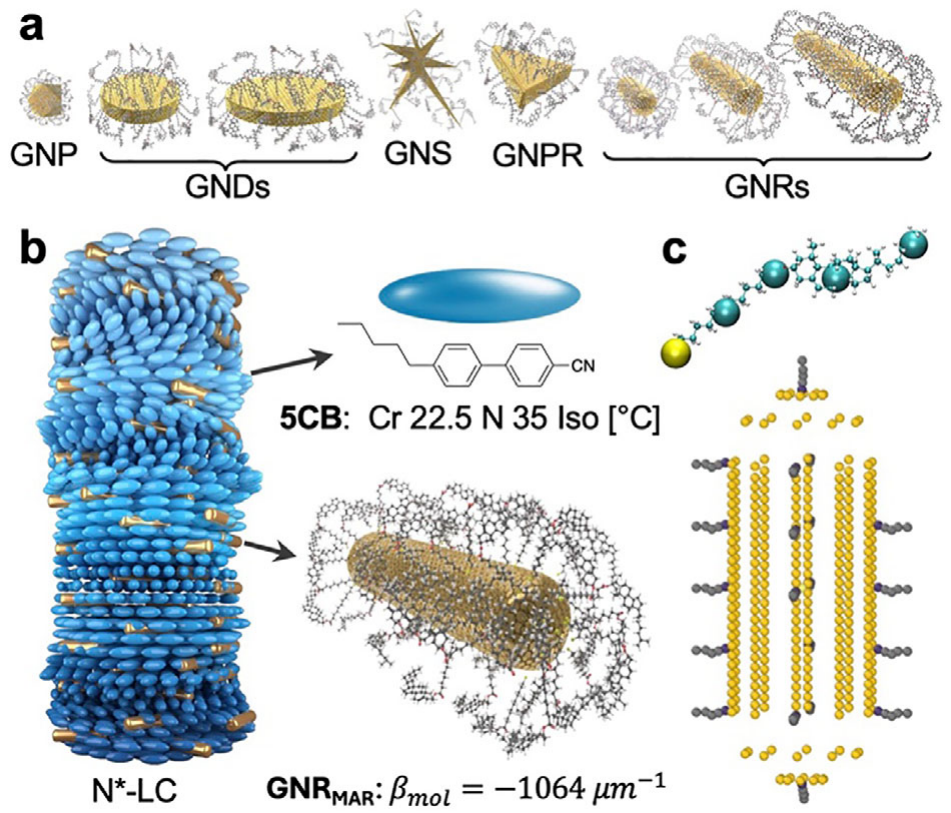
(a) Variety of gold nanoshapes with a chiral cholesterol‐thiol ligand shell: gold nanoparticles (GNP) varying in diameter, gold nanodisks (GND) and gold nanorods (GNR) varying in aspect ratio as well as gold nanostars (GNS) and gold nanoprisms (GNPR) previously studied (b) as chiral solutes in 5CB to induce a chiral nematic liquid crystal (N*‐LC) phase (shown for GNRs with medium aspect ratio (GNR_MAR_) in 5CB—not necessarily to scale). (c) Coarse‐grained (CG) representations of the cholesterol‐thiol ligand and the CG‐ligand‐capped GNR_MAR_ used for the calculation of $|G_{oa,max}^{a}S^{XN}|$ with the best shape match to 5CB (almost identical *IPR*_2D_) and among the highest |β_*mol*_| value ever recorded. The negative sign for β_*mol*_ indicates the experimentally observed left‐handed twist of the induced N*‐LC phase [22, 23].

Consequently, the degree of close correlation between *S^XN^* and β_*mol*_ provided direct insights into the critical role of shape complementarity in chirality transfer [22]. Note that *S^XN^* is a scalar quantity to the degree that the orientation of one particle with respect to another is nonzero and scales invariantly under all spatial transformations, even in the achiral case. Furthermore, *IPR*_2D_ and thus *S^XN^*, like all the previously discussed shape chirality measures, remains invariant with changes in size. The closely matching trends between our experimental β_*mol*_ data with the calculated $|G_{oa,max}^{a}S^{XN}|$ values verified that the size and aspect ratio of chiral ligand–decorated nanoshapes affected in very predictable ways the chiral perturbation induced in an achiral host medium given by an N‐LC phase. Considering the range of gold nanoshapes tested (Figure 1a), we firmly established a systematic correlation between purely geometric concepts of an independently calculated chirality indicator and experimental chirality transfer data. While each surveyed gold nanoshape capped with a cholesterol‐thiol ligand outperformed the neat organic chiral cholesterol molecule with respect to its ability and effectiveness to transfer chirality to the N‐LC host medium (5CB, 4‐cyano‐4’‐pentylbiphenyl), not surprisingly, the medium aspect ratio GNR_MAR_ with the largest $|G_{oa,max}^{a}S^{XN}|$ values and the closest match between $IPR_{2D}^{\mathrm{GNR}}$ and $IPR_{2D}^{5\mathrm{CB}}$ turned out to be the most powerful chiral inducer with an astonishingly high value of |β_*mol*_| = 1064 µm^−1^ (Figure 1b,c) [22, 23].

To further substantiate that (a) solute‐solvent shape commensurability is a prerequisite for efficacious chiral transfer in such condensed matter systems and (b) that our approach of predicting this via the calculation of an independent chirality measure such as $|G_{oa,max}^{a}S^{XN}|$ is also accurate for other solvent‐solute shape combinations in condensed matter systems, we sought to validate our approach by using a corresponding room‐temperature discotic nematic liquid crystal (N_D_‐LC) phase—formed by disc‐like (discotic) rather than rod‐like (calamitic) molecules [24, 25]—and three distinct gold nanoshapes (GNR, GNPR, and GND) all monolayer‐capped with a suitable (i.e., structurally‐related) chiral discotic molecule ligand shell [26].

## 2 Results and Discussion

**FIGURE 2:**
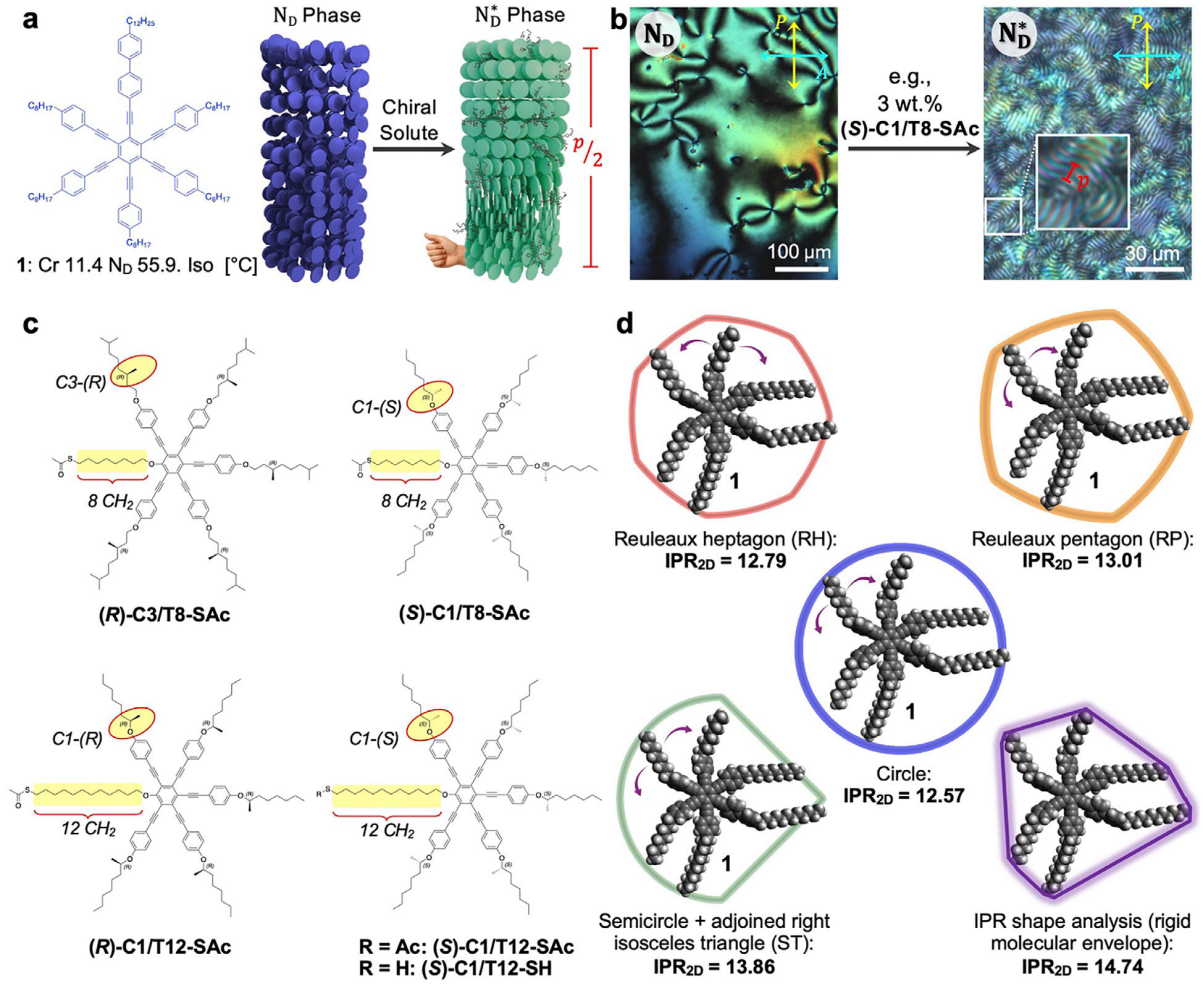
(a) Chemical structure of **1** [27, 28, 29], phase sequence, and phase transition temperatures (Cr = crystal, N_D_ = discotic nematic, and Iso = isotropic liquid phase), and models of the achiral N_D_ and the induced N_D_* phase. (b) Textural characteristics observed by POM for the N_D_ phase before and after addition of a chiral solute such as (*S*)‐C1/T8‐SAc. Such fingerprint textures with the prerequisite boundary conditions were used to measure *p* of the induced N_D_* phase. (c) Chemical structures of the four pentakis(4‐alkyloxyphenylethynyl)benzene aliphatic thioacetate‐based chiral solutes with the adjusted structural parameters highlighted: position/configuration of chiral centers (C3 vs. C1) and length of aliphatic thiol tether (T = 8 or 12). (d) The five 2‐D shapes considered to mimic **1** and their calculated *IPR*_2D_ values.

The room‐temperature N_D_‐LC material **1** [27, 28, 29], based on a hexaynylbenzene core with one of the 4‐alkylphenylethynyl peripherals de facto replaced by a 4’‐alkylbiphenylethynyl group, is equally capable of forming a chiral N_D_* phase when a suitable chiral solute with a disc‐like overall shape is introduced (Figure 2a) [30]. The textures observed by polarized optical microscopy (POM) for the achiral N_D_ and for the induced chiral N_D_* phase resemble those of the N and N* phase and analogously allow for the measurement of *p* and the subsequent calculation of β_*mol*_ (Figure 2b) [30]. However, prior to examining the role of gold nanoparticle shape with respect to chirality transfer efficacy, we examined the role of two consequential structural parameters of chiral ligands (dopants) that closely resemble the structure of N_D_‐LC host **1**: (a) position and configuration of the chiral centers in the pentakis(4‐alkyloxyphenyl‐ethynyl)benzene derivatives that are structurally related to those previously studied by Heppke et al. [26, 30]. and (b) the length of the aliphatic thiol tether that allowed us, by elongation from 8 to 12 methylene groups, to more closely match the shape of **1** (Figure 1c). The synthesis and characterization of **1** and of the four, non‐mesogenic [30] chiral thioacetates (*S*)‐ or (*R*)‐C_x_T_y_‐SAc with x = 1 or 3 and y = 8 or 12 (one of which will serve as the precursor for the thiolate ligand shell, *vide infra*) is detailed in Sections S1 and S2, further including X‐ray diffraction studies of the N_D_ phase formed by **1** in Section S3.

To calculate *S^XN^* and determine the relative shape commensurability between **1** and the chiral thioacetates (rather than using the thiols that are prone to oxidation to disulfides) as well as between **1** and the capped gold nanoshapes, we considered five 2‐D shapes that could potentially best embody the shape of **1**. The *IPR*_2D_ values calculated for the five 2‐D shapes [31, 32], assumed among the closest representations with the overlayed, energy‐minimized structure of **1**, are provided in Figure 2d. Because the aliphatic chains of **1** in the fluid N_D_ phase have a significant degree of conformational freedom, we considered the circle the best match and the semicircle with an adjoined right isosceles triangle (ST) a close match for a more rigid shape of **1**. The Reuleaux penta‐ and heptagon (RP and RH), for which we will show our calculations as well, simply give too much empty space when ‘filled’ with **1**. However, we also considered a rigid molecular envelope projected onto the principle XY plane of the tensor of inertia Eigenframe to calculate the convex hull perimeter and enclosed area using a Python code Section S4.

As determined next, we used the most potent chiral inducer among the three general chiral thioacetate structures with C1‐(*S*) configurations in the aliphatic chains and the longer aliphatic thiol tether (after deprotection of the acetyl group; (*S*)‐C1/T12‐SH) to surface‐functionalize the initially surfactant‐capped gold nanoshapes [33, 34, 35, 36]. Specifics of the synthesis and full characterization are provided in Sections S5 and S6.

**FIGURE 3:**
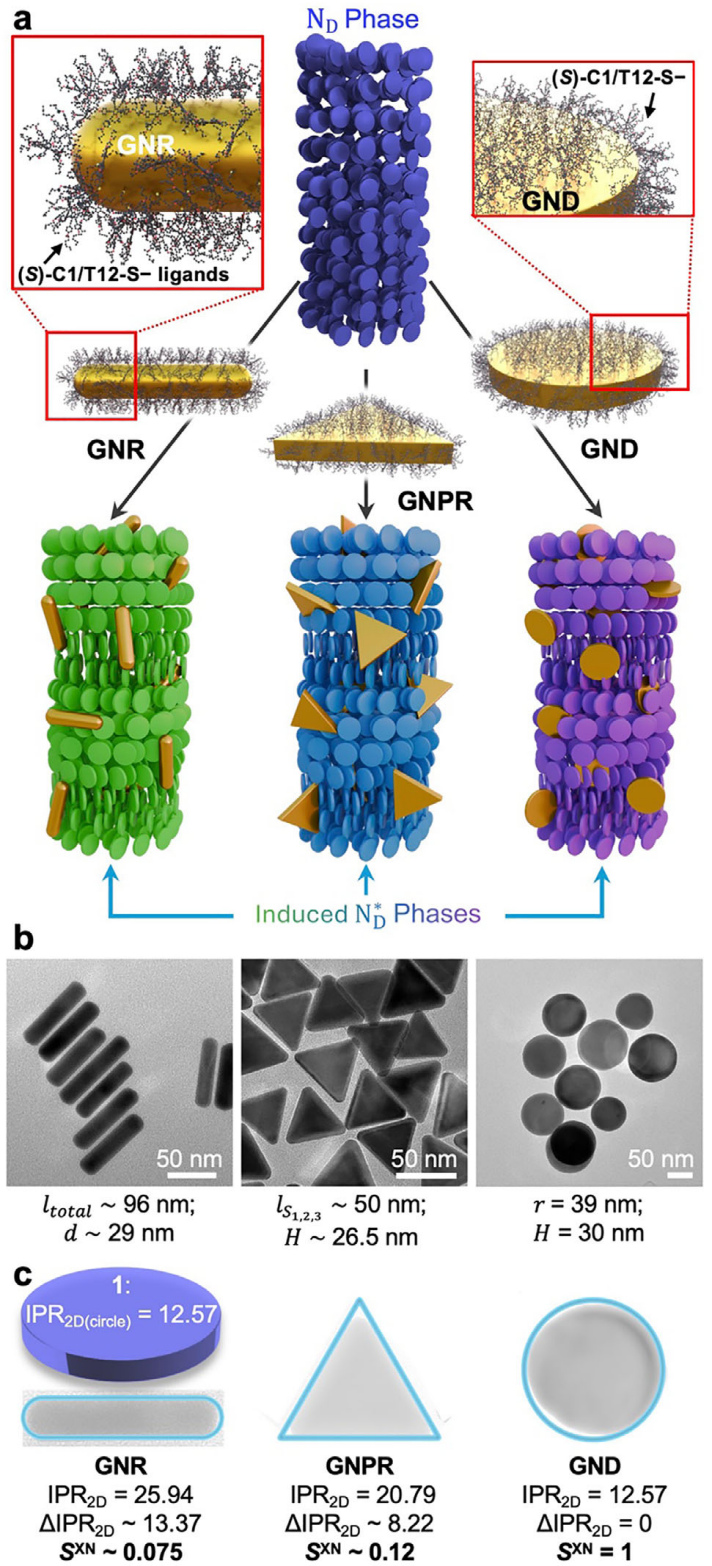
(a) Models of the N_D_ phase formed by **1** and the induced N_D_* phase after dispersion of the (*S*)‐C1/T12‐S− monolayer‐capped gold nanoshapes. (b) TEM images of the (*S*)‐C1/T12‐S‐capped GNRs, GNPRs, and GNDs—average dimensions given below (*l_total_* = total length and *d* = diameter for GNR, $l_{s_{1,2,3}}$ = side length for GNPR, *H* = height or thickness for GND and GNPR; for further TEM images see Section S4). (c) Shape analysis ($\Delta IPR_{2D}^{XN}$ values) used for the calculation of *S^XN^* used the *IPR*_2D_ value for the circular shape of **1**.

All thioacetates and monolayer‐capped gold nanoshapes (GNR, GNPR, and GND) were then each dispersed in **1** at a range of concentrations (ranging from 1 wt.% up to 30 wt.% for (*R*)‐C3/T8‐SAc and up to 5 wt.% for all others) to measure *p* of the induced N_D_* phase (Figure 3a), determine β_*mol*_, and finally compare the trends, here individually for the thioacetates and the nanoshapes, with the calculated $|G_{oa,max}^{a}S^{XN}|$ data. Transmission electron microscopy (TEM) images and the average dimensions of the gold nanoshapes obtained by image analysis using ImageJ [37] are given in Figure 3b. Assuming the aforementioned circular shape for **1**, the calculated values for $IPR_{2D}^{X}$, $|\Delta IPR_{2D}^{XN}|$, and *S^XN^* are shown in Figure 3c.

**FIGURE 4:**
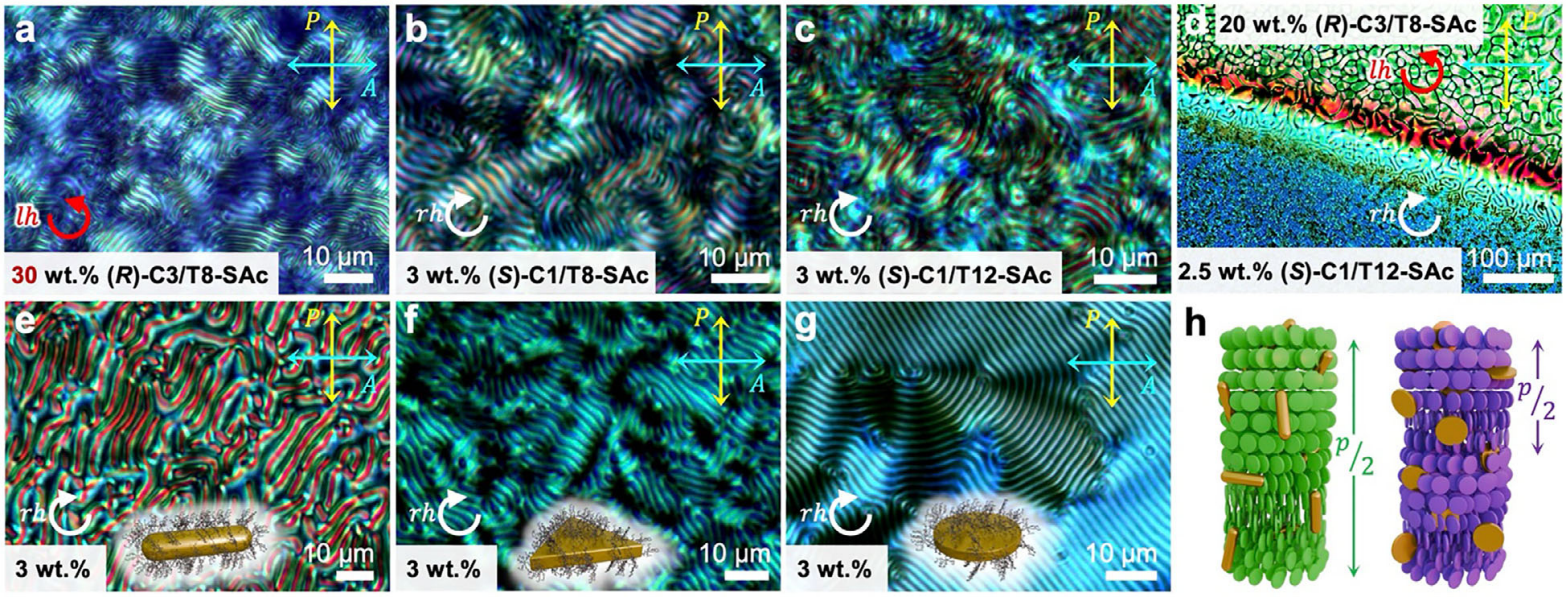
Polarized light optical photomicrographs (crossed polarizer *P* and analyzer *A*) between glass slides treated to induce homeotropic boundary conditions of the N_D_*‐LC phase of **1** induced by the addition of: (a) 30 wt.% (*R*)‐C3/T8‐SAc, (b) 3.0 wt.% (*S*)‐C1/T8‐SAc, (c) 3.0 wt.% (*S*)‐C1/T12‐SAc, (d) 20 wt.% (*R*)‐C3/T8‐SAc and 2.5 wt.% (*S*)‐C1/T12‐SAc in a contact preparation (see discontinuation and formation of an achiral N_D_ phase in the contact zone), (e) 3.0 wt.% (*S*)‐C1/T12‐S−GNR, (f) 3.0 wt.% (*S*)‐C1/T12‐S−GNPR, and (g) (*S*)‐C1/T12S−GND (nanoshapes schematically shown in the bottom of each image) on cooling from the isotropic liquid phase at the same reduced temperature *T* − *T_NI_* = −5°C (5°C below the isotropic‐N_D_* phase transition). Curly arrows indicate the handedness of the N_D_* phase. (h) Models showing the difference in *p* of the N_D_* phase of **1** doped with GNR versus GND. Additional POM images, including for contact preparations to determine the handedness of the induced N_D_* phases are given in Sections S7 and S8. Further, thin film induced circular dichroism spectra of the induced N_D_* phases supporting the handedness assignments are given in Section S7.3.

Representative POM images showing the characteristic fingerprint textures of the induced N_D_* phases of the chiral thioacetates are given in Figure 4a–d. Considering position and configuration of the chiral centers as well as the handedness of induced N_D_* phases experimentally determined for the pentakis(4‐alkyloxyphenylethynyl)benzene derivatives, we here confirm, as already reported by Heppke et al. [30], that the quasi‐empirical Gray & McDonnell rules established for N* phase of rod‐like chiral molecules (or solutes) [38] also apply to chiral solutes in N_D_* phase (Figures 4a–d). Moreover, moving the chiral centers away from the core‐chain junctures, thereby reducing any restraint of rotation about the chiral center, reduces the effects of chirality in aliphatic side chains as established by Goodby and coworkers [39]. Evidence of this diminished chirality were seen in the measurements of *p* of the induced N_D_* phase depending on the concentration of chiral solutes: p∼ 3.5 µm at 30 wt.% of (*R*)‐C3/T8‐SAc versus p∼ 4.5 µm at 3.0 wt.% of (*S*)‐C1/T8‐SAc (Table 1). As predicted by our new modus operandi, $|G_{oa,max}^{a}S^{XN}|$, a closer shape match between **1** and the chiral solute molecules—realized by elongating the aliphatic thiol tether from (CH_2_)_8_ to (CH_2_)_12_—leads to a further reduction in *p* at identical chiral solute concentrations for (*R*)‐ and (*S*)‐C1/T12SAc (*vide infra*). Accordingly, POM images captured for the gold nanoshapes capped with (*S*)‐C1/T12‐S− (Figures 4e–g) immediately convey that at an identical concentration of 3.0 wt.%, the GNDs induce a much tighter *p* than the GNRs (Figure 4h) and a slightly tighter *p* than the GNPRs. The calculated values for β_*mol*_, $|G_{oa,max}^{a}|$, *S^XN^*, and $|G_{oa,max}^{a}S^{XN}|$ are given in Table 1. As in all previous studies [21, 22, 23, 40, 41], the β_*mol*_ values obtained for the nanoshapes capped with (*S*)‐C1/T12‐S− continue to exceed those of the free organic chiral solutes.

**TABLE 1:** Composition, chiral center configuration, experimental data for *p* (for one representative concentration of chiral solute) and *β*_mol_, and values calculated for $|G_{oa,max}^{a}|$, *S^XN^*, and $|G_{oa,max}^{a}S^{XN}|$.

Chiral solute	Configuration	*β*_mol_ (µm^−1^)^a^	$|G_{oa,max}^{a}|$	*S^XN^*	$|G_{oa,max}^{a}S^{XN}|$	*p* (µm) at *x* wt.%^e^
(*R*)‐C3/T8‐SAc	(*R*,*R*,*R*,*R*,*R*)	−0.93^b^	0.78	1.0^c^	0.78	3.58 at 30.0 wt.%
(*S*)‐C1/T8‐SAc	(*S*,*S*,*S*,*S*,*S*)	+6.89^b^	0.99	1.0^c^	0.99	4.48 at 3.0 wt.%
(*R*)‐C1/T12‐SAc	(*R*,*R*,*R*,*R*,*R*)	−14.27^b^	1.20	1.0^c^	1.20	2.39 at 3.0 wt.%
(*S*)‐C1/T12‐SAc	(*S*,*S*,*S*,*S*,*S*)	+14.27^b^	1.20	1.0^c^	1.20	2.39 at 3.0 wt.%
(*S*)‐C1/T12‐S−GNR	(*S*,*S*,*S*,*S*,*S*)	+15.05	9850	0.06^d^	591	9.23 at 3.0 wt.%
(*S*)‐C1/T12‐S−GNPR	(*S*,*S*,*S*,*S*,*S*)	+38.02	8194	0.18^d^	1475	3.27 at 3.0 wt.%
(*S*)‐C1/T12‐S−GND	(*S*,*S*,*S*,*S*,*S*)	+67.84	2669	1.0^d^	2669	3.01 at 3.0 wt.%

^a^ β_*mol*_ is the helical twisting power value when the dimensionless mole fraction (*x_solute_*) of the chiral additive is used. For the current dataset, *x_solute_* = *x_ligand_* because the nanoshapes are intrinsically achiral. To determine *x_ligand_*, experimental TGA data were used (Supporting Information, Section S6.3). A negative sign indicates a left‐handed, a positive sign a right‐handed N_D_* phase.

^b^ Handedness in agreement with that previously reported by Heppke [30].

^c^ Assuming a circular shape for both **1** and the chiral organic solutes. Other *S^XN^* values for the ST, RH and RP shapes ($S_{ST}^{XN}$, $S_{RH}^{XN}$, and $S_{RP}^{XN}$) as well as a rigid molecular envelope ($S_{mol}^{XN}$) assumed for **1** are given in the Supporting Information (Section S9).

^d^ Assuming a circular shape for **1** (see Figure 3c).

^e^ Values of *p* obtained using glass substrates treated to favor homeotropic anchoring conditions, as seen previously [16, 21, 22, 23, 40, 41], were obtained with an accuracy better than ±0.3 µm.

However, to evaluate the power and accuracy of our predictive method for chirality transfer efficacy, we now need to compare trends of $|G_{oa,max}^{a}S^{XN}|$ and |β_*mol*_|, separately for the free organic solutes and the three thiol‐monolayer‐capped nanoshapes (for plots of the inverse pitch, 1/*p*, vs. mole fraction to calculate |β_*mol*_| and details of the $|G_{oa,max}^{a}|$ calculations see Sections S10 and S11). Justification for this separate treatment of the two datasets is justified by the fact that we need to ensure that the isotropic to N_D_ phase transition temperature, *T_NI_*, is practically the same for each of the two series of chiral solutes, and that *p* was measured at the same temperature below *T_NI_*, which was congruent to the experiment conditions. Thus, we assumed the order parameter〈*P*_2_〉 of the induced N_D_*‐LC phase to be identical in either series for each system.

**FIGURE 5:**
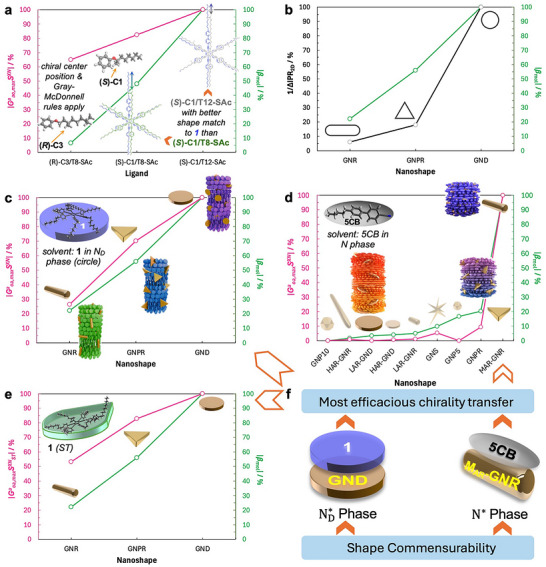
Comparisons of $|G_{oa,max}^{a}S^{XN}|$ or *S^XN^* with experimentally determined values for |β_*mol*_|. (a) Trends of $|G_{oa,max}^{a}S^{XN}|$ in comparison to |β_*mol*_| for the three organic chiral solutes. (b) Trends of $S^{XN}=1/\Delta IPR_{2D}^{XN}$ in comparison to |β_*mol*_| for the three gold nanoshapes. (c) Trends of $|G_{oa,max}^{a}S^{XN}|$ versus |β_*mol*_| assuming a circular shape of **1**, models of the induced N_D_* phase with decreasing pitch from the GNRs to the GNDs. (d) Trends of $|G_{oa,max}^{a}S^{XN}|$ versus |β_*mol*_| of an extended series of gold nanoshapes capped with a chiral cholesterol‐thiol ligands shell studied in the N‐phase formed by 5CB (data from ref 22). (e) Trends of $|G_{oa,max}^{a}S^{XN}|$ versus |β_*mol*_| assuming an ST shape of **1** (plots using the *S^XN^* values for the RH and RP shapes ($S_{RH}^{XN}$ and $S_{RP}^{XN}$) as well as the rigid molecular envelope for **1** ($S_{mol}^{XN}$) are given in the Supporting Information, Sections S11.5 and S11.6). (f) Depiction of the role of shape commensurability in chirality transfer in both the induced N* and N_D_* phases.

Supposing that **1** and each of the chiral organic solutes have a similar and, on average, circular shape, the trends of the computed $|G_{oa,max}^{a}S^{XN}|$ and |β_*mol*_| are at least in reasonable agreement insofar as (*R*)‐C3/T8‐SAc is the weakest (lowest value of $|G_{oa,max}^{a}|$) and (*S*)‐ or (*R*)‐C1/T12‐SAc (differing only in the handedness of the induced N_D_* phase) the strongest chiral solutes (highest value of $|G_{oa,max}^{a}|$), and therefore the chiral inducers with the highest value of |β_*mol*_| (Figure 5a). Accordingly, even without making a specific argument about shape complementarity (assuming for the organic chiral solutes and solvent **1** that *S^XN^* = 1), $|G_{oa,max}^{a}|$, as a pseudoscalar chirality indicator derived from the molecules’ geometry, captures both the diminished chirality of the chiral center at C‐3 for (*R*)‐C3/T8‐SAc as well as the closer shape complementarity between **1** and (*S*)‐C1/T12‐SAc. This also holds if a rigid molecular envelope is assumed for **1** as well as the chiral organic solutes (Section S4, S9, and Table S2). In fact, trends for $|G_{oa,max}^{a}S_{mol}^{XN}|$ and |β_*mol*_| are in even closer agreement than in the case where a circular shape for **1** and the chiral solutes is presumed (Section S11.7, Figure S49a). However, the experimentally established conformational freedom of the aliphatic chains in the N_D_* phase (see XRD data in Section S3, Figure S28, indicated by the curled arrows in Figure 2d) somewhat limit the usefulness of a representation of these molecules by a rigid molecular envelope to calculate *S^XN^*.

Finally, for the series of (*S*)‐C1/T12‐S−capped gold nanoshapes, while the correlation between *S^XN^* and |β_*mol*_| is existing but weak (Figure 5b), the almost perfect alignment of the trends between $|G_{oa,max}^{a}S^{XN}|$ and |β_*mol*_| while assuming a circular shape of **1** (Figure 5c) offers new proof that a shape commensurability‐corrected, computed chirality indicator (based purely on geometric information) serves as an exceptional predictor for the efficacy of the ways chiral surface‐modified nanoshapes affect chiral perturbations in achiral host environments. While the medium aspect ratio GNR_MAR_ (*AR* = 4.3) with an *IPR*_2D_ matching the one of the rod‐like N‐LC 5CB were, as predicted by $|G_{oa,max}^{a}S^{XN}|$ and experimentally proven via |β_*mol*_|, the most powerful chiral inducers in the N‐LC series (Figure 5d) [22], now GNDs with an *IPR*_2D_ matching the one of the N_D_‐LC **1**, are the most powerful inducers of N_D_* phases with the tightest values of the induced *p*. Even assuming the shape of **1** is a semicircle with an adjoined right isosceles triangle (ST), trends for $|G_{oa,max}^{a}S^{XN}|$ and |β_*mol*_| show a reasonably close match (Figure 5e), which continues to highlight the role of shape commensurability in predicting chirality transfer efficacy in these condensed phase systems (Figure 5f). Furthermore, trends for $|G_{oa,max}^{a}S^{XN}|$ and |β_*mol*_| are preserved no matter what shape is assumed for **1**, even if a rigid molecular envelope is chosen (Section S11.6). To further support these arguments, underscoring that while *S^XN^* is scale invariant for GNDs when assuming a circular shape for **1** and $|G_{oa,max}^{a}|$ scales with a change in GND *AR*, we prepared a second set of GNDs with an almost identical diameter of *d* = 74 nm and a lower height of *H* = 14 nm (i.e., a higher *AR* = 5.4; see Section S12.1). Our calculations illustrate that $|G_{oa,max}^{a}|$ rises with an increasing *AR* of the GNDs. Thus, if our theory holds, |β_*mol*_| for GNDs with a higher *AR* (GND_AR ∼ 5.4_) should increase as well, which is exactly what was experimentally observed. The (*S*)‐C1/T12‐S−capped GND_AR ∼ 5.4_ induce an N_D_* phase with a tighter pitch at lower concentrations of the GNDs (e.g., *p* = 2.64 µm at 2.5 wt.% GND_AR ∼ 5.4_ vs. *p* = 3.01 µm at 3.0 wt.% GND_AR ∼ 2.6_) and yield a higher value of |β_*mol*_| (|β_*mol*_| = 103.6 µm^−1^; Section S12.3, Figure S51 and S52). Future studies will further investigate series of GNDs, focusing on systematically varying both *H* and *d* (including, if experimentally accessible, GNDs that represent perfect dilations apart from the aliphatic tether chain flexibility, i.e., *H* and *d* for the bare nanodiscs scale identically).

## 3 Conclusions

Combined, all now existing datasets [16, 21, 22, 23, 40, 41] for chiral ligand‐capped gold nanoshapes inducing N*‐LC and N_D_*‐LC phases not only demonstrate the predictive power of $|G_{oa,max}^{a}S^{XN}|$, but they also further underpin the critical importance of shape [42, 43, 44] and shape commensurability in chirality transfer. This new modus operandi for predicting chirality transfer efficacy permits precise forecasts well beyond the previous, more empirical arguments that similarity between chiral solutes and achiral hosts results in effective chirality transfer. The argument we are making that is supported by the current datasets is that “similar” means: similar in shape of the 2‐D projections but invariant with changes in overall size with respect to the shape correcting factor *S^XN^* introduced in this study. Furthermore, computing the values for $|G_{oa,max}^{a}S^{XN}|$ for nanoshapes capped with either type of chiral ligand shell (tailored to the N‐LC or the N_D_‐LC host) allowed us to *a priori* predict that GNPRs are relatively effective chiral solutes in either of these two types of nematic LC phases; arguably impossible to be predicted with any of the aforementioned empirical arguments. Furthermore, while nanoscale gold particles with commensurable shape significantly amplify chirality in each case (in the induced N*‐LC and N_D_*‐LC phases), independent calculations [45, 46] and measurements of the elastic constants for splay, twist, and bend (*K*_11_, *K*_22_, and *K*_33_) for N‐ and N_D_‐phases [47] already predicted that it is indeed significantly easier to twist rod‐like than disc‐like molecules in their respective nematic phases, thus accounting for the lower |β_*mol*_| values found here for the most powerful GND chiral solutes in the induced N_D_* phase formed by **1** (|β_*mol*_| ranging from 68 µm^−1^ to 103 µm^−1^) in comparison to the most potent GNR_MAR_ inducing the N* phase formed by 5CB (|β_*mol*_| = 1064 µm^−1^) [22].

We reason that the extension of this straightforwardly applied geometric approach, and potentially other closely related approaches [48, 49, 50, 51], to systems beyond soft matter, reenforced by machine learning methods, could provide a vital tool for experimentalists in the many fields where chirality transfer is essential. These may include the continued development of chiral nanomaterials with inherently chiral shapes [52, 53, 54], chiral nanomaterial catalysts for solid‐ or liquid‐phase asymmetric synthesis [55], or for the development of chiral stationary phases required for the chiral separation of enantiomers [56, 57]. Finally, using a highly predictive geometric tool should facilitate a better understanding of countless chiral molecular recognition and amplification events occurring in living systems [58] or exploited in chiral nanophotonics [59], plasmonics [60], metamaterials, and [61] metal‐organic frameworks (MOFs) [62].

## Author Contributions

**Gourab Acharjee**: writing – original draft, validation, visualization, data curation, formal analysis, investigation, writing – review and editing. **Lara Querciagrossa**: investigation, writing – original draft, validation, visualization, formal analysis, data curation, writing – review and editing. **Grace A. R. Rohaley**: investigation, visualization, validation, writing – review and editing. **Nicholas M. Kamuti**: writing – review and editing, data curation, investigation. **Ashwathanarayana Gowda**: investigation, writing – review and editing, data curation.**Suraj Kumar Pathak**: investigation, writing – review and editing, formal analysis, data curation. **Asmita Shah**: investigation, writing – review and editing, formal analysis, data curation. **Kun Zhang**: investigation, data curation. **Jianfang Wang**: methodology, funding acquisition, writing – review and editing, supervision, resources. **Claudio Zannoni**: conceptualization, writing – original draft, writing – review and editing, methodology, formal analysis, supervision, resources. **Torsten Hegmann**: conceptualization, funding acquisition, writing – original draft, writing – review and editing, visualization, validation, methodology, formal analysis, project administration, supervision, resources.

## Conflicts of Interest

The authors declare no conflicts of interest.

## Supporting information



The authors have cited additional references within the Supporting Information [63, 64, 65, 66, 67, 68, 69, 70, 71].
**Supporting File**: anie72702‐sup‐0001‐SuppMat.pdf.

## Data Availability

The data that supports the findings of this study are available in the supplementary material of this article.

## References

[1] A. B. Buda , T. A. Derheyde , and K. Mislow , “On Quantifying Chirality,” Angewandte Chemie International Edition 31 (1992): 989–1007, 10.1002/anie.199209891.

[2] E. Abraham and A. Nitzan , “Molecular Chirality Quantification: Tools and Benchmarks,” Journal of Chemical Physics 160 (2024): 164104, 10.1063/5.0200716. 38651805

[3] M. Hwang , H. Jung , and J. Y. Kim , “Chirality Quantification for High‐Performance Nanophotonic Biosensors,” Small Methods 9 (2025): 2500112, 10.1002/smtd.202500112. 40200644

[4] G. Gilat , “On quantifying chirality — Obstacles and Problems Towards Unification,” Journal of Mathematical Chemistry 15 (1994): 197–205, 10.1007/BF01277559.

[5] P. A. Guye , “Influence de la Constitution Chimique Des Dérivés Du Carbone sur Le Sens et les Variations De Leur Pouvoir Rotatoire,” Comptes Rendus Hebdomadaires des Seances De L Academie Des Sciences 110 (1890): 714–716.

[6] P. Murray‐Rust , H. B. Burgi , and J. D. Dunitz , “Distortions of M_4_ molecules From _d_ Symmetry. I. Kernel, Co‐kernel and Averaged Configurations,” Acta Crystallographica Section B, Structural Science 34 (1978): 1787–1793, 10.1107/S0567740878006718.

[7] P. Murray‐Rust , H. B. Burgi , and J. D. Dunitz , “Distortions of M_4_ Molecules From _d_ Symmetry. II. Analysis of PO_4_ , SO_4_ and AlCl_4_ Species,” Acta Crystallographica Section B, Structural Science 34 (1978): 1793–1803, 10.1107/S056774087800672X.

[8] P. Murray‐Rust , H. B. Burgi , and J. D. Dunitz , “Description of Molecular Distortions in Terms of Symmetry Coordinates,” Acta Crystallographica Section A, Crystal Physics, Diffraction, Theoretical and General Crystallography 35 (1979): 703–713, 10.1107/S0567739479001686.

[9] A. B. Buda and K. Mislow , “A Hausdorff Chirality Measure,” Journal of the American Chemical Society 114 (1992): 6006–6012, 10.1021/ja00041a016.

[10] E. O. Yewande , M. P. Neal , and R. Low , “The Hausdorff Chirality Measure and a Proposed Hausdorff Structure Measure,” Molecular Physics 107 (2009): 281–291, 10.1080/00268970902835611.

[11] F. Hausdorff , Set Theory (Chelsea Publishing Company, 1962).

[12] J. J. Pelayo , R. L. Whetten , and I. L. Garzón , “Geometric Quantification of Chirality in Ligand‐Protected Metal Clusters,” Journal of Physical Chemistry C 119 (2015): 28666–28678, 10.1021/acs.jpcc.5b10235.

[13] V. E. Kuzmin , I. B. Stelmakh , I. V. Yudanova , D. V. Pozigun , and M. B. Bekker , “Quantitative Aspects of Chirality. II. Analysis of Dissymmetry Function Behaviour With Different Changes in the Structure of the Model Systems,” Journal of Physical Organic Chemistry 5 (1992): 299–307, 10.1002/poc.610050604.

[14] P. G. Mezey , “The Degree of Similarity of Three‐dimensional Bodies: Application to Molecular Shape Analysis,” Journal of Mathematical Chemistry 7 (1991): 39–49, 10.1007/BF01200814.

[15] M. A. Osipov , B. T. Pickup , and D. A. Dunmur , “A New Twist to Molecular Chirality: Intrinsic Chirality Indices,” Molecular Physics 84 (1995): 1193–1206, 10.1080/00268979500100831.

[16] A. Nemati , S. Shadpour , L. Querciagrossa , et al., “Chirality Amplification by Desymmetrization of Chiral Ligand‐capped Nanoparticles to Nanorods Quantified in Soft Condensed Matter,” Nature Communications 9 (2018): 3908, 10.1038/s41467-018-06400-0. PMC6156227 30254259

[17] A. Pietropaolo , L. Muccioli , R. Berardi , and C. Zannoni , “A Chirality Index for Investigating Protein Secondary Structures and Theirtime Evolution,” Proteins 70 (2008): 667–677, 10.1002/prot.21578. 17879347

[18] M. Solymosi , R. J. Low , M. Grayson , and M. P. Neal , “A Generalized Scaling of a Chiral Index for Molecules,” Journal of Chemical Physics 116 (2002): 9875–9881, 10.1063/1.1476321.

[19] A. B. Harris , R. D. Kamien , and T. C. Lubensky , “Molecular Chirality and Chiral Parameters,” Review of Modern Physics 71 (1999): 1745–1757, 10.1103/RevModPhys.71.1745.

[20] A. P. C. Almeida , L. Querciagrossa , P. E. S. Silva , et al., “Reversible Water Driven Chirality Inversion in Cellulose‐based Helices Isolated From Erodium awns,” Soft Matter 15 (2019): 2838–2847, 10.1039/C8SM02290A. 30869683

[21] A. Nemati , S. Shadpour , L. Querciagrossa , T. Mori , C. Zannoni , and T. Hegmann , “Highly Sensitive, Tunable Chirality Amplification Through Space Visualized for Gold Nanorods Capped With Axially Chiral Binaphthyl Derivatives,” ACS Nano 13 (2019): 10312–10326, 10.1021/acsnano.9b03787. 31424907

[22] A. Nemati , L. Querciagrossa , C. Callison , et al., “Effects of Shape and Solute‐solvent Compatibility on the Efficacy of Chirality Transfer: Nanoshapes in Nematics,” Science Advances 8 (2022): eabl4385, 10.1126/sciadv.abl4385. 35080976 PMC8791610

[23] A. Sharma , T. Mori , A. Nemati , et al., “The Significance of Nanoparticle Shape in Chirality Transfer to a Surrounding Nematic Liquid Crystal Reporter Medium,” Materials Advances 3 (2022): 3346–3354, 10.1039/D2MA00093H.

[24] S. Laschat , A. Baro , N. Steinke , et al., “Discotic Liquid Crystals: From Tailor‐Made Synthesis to Plastic Electronics,” Angewandte Chemie International Edition 46 (2007): 4832–4887, 10.1002/anie.200604203. 17568461

[25] T. Wöhrle , I. Wurzbach , J. Kirres , et al., “Discotic Liquid Crystals,” Chemical Reviews 116 (2016): 1139–1241. 26483267 10.1021/acs.chemrev.5b00190

[26] M. Langner , K. Praefcke , D. Kruerke , and G. Heppke , “Chiral Radial Pentaynes Exhibiting Cholesteric Discotic Phases,” Journal of Materials Chemistry 5 (1995): 693–699, 10.1039/JM9950500693.

[27] H. H. Chen , H. A. Lin , S. C. Chien , et al., “Single‐Component Room‐Temperature Discotic Nematic Liquid Crystals Formed by Introducing an Attraction‐Enhancing in‐Plane Protrusion Onto the Hexa(phenylethynyl)Benzene Core,” Journal of Materials Chemistry 22 (2012): 12718–12722, 10.1039/c2jm16263f.

[28] K. Praefcke , B. Kohne , and D. Singer , “Hexaalkynyltriphenylene: A New Type of Nematic‐Discotic Hydrocarbon,” Angewandte Chemie International Edition 29 (1990): 177–179, 10.1002/anie.199001771.

[29] S. Marguet , D. Markovitsi , D. Goldmann , D. Janietz , K. Praefcke , and D. Singer , “Spectroscopic Properties of Nematic Discotic Phenylethynylbenzene Derivatives: Symmetry Effects,” Journal of the Chemical Society, Faraday Transactions 93 (1997): 147–155, 10.1039/a603265f.

[30] C. J. Booth , D. Kruerke , and G. Heppke , “Highly Twisting Enantiomeric Radial Multiyne Dopants for Discotic Liquid‐Crystalline Systems,” Journal of Materials Chemistry 6 (1996): 927–934, 10.1039/JM9960600927.

[31] K. Ball , “Volume Ratios and a Reverse Isoperimetric Inequality,” Journal of the London Mathematical Society 44 (1991): 351–359, 10.1112/jlms/s2-44.2.351.

[32] M. M. Conroy , “A Table of Isoperimetric Ratios” https://sites.math.washington.edu/~conroy/isoperimetrics/isoperimetrics.pdf 2025.

[33] J. E. Millstone , S. J. Hurst , G. S. Métraux , J. I. Cutler , and C. A. Mirkin , “Colloidal Gold and Silver Triangular Nanoprisms,” Small 5 (2009): 646–664, 10.1002/smll.200801480. 19306458

[34] B. Nikoobakht and M. A. El‐Sayed , “Preparation and Growth Mechanism of Gold Nanorods (NRs) Using Seed‐ mediated Growth Method,” Chemical Materials 15 (2003): 1957–1962, 10.1021/cm020732l.

[35] X. M. Cui , F. Qin , Q. F. Ruan , X. L. Zhuo , and J. F. Wang , “Circular Gold Nanodisks With Synthetically Tunable Diameters and Thicknesses,” Advanced Functional Materials 28 (2018): 1705516, 10.1002/adfm.201705516.

[36] S. Umadevi , X. Feng , and T. Hegmann , “Large Area Self‐Assembly of Nematic Liquid‐Crystal‐Functionalized Gold Nanorods,” Advanced Functional Materials 23 (2013): 1403.

[37] C. A. Schneider , W. S. Rasband , and K. W. Eliceiri , “NIH Image to ImageJ: 25 Years of Image Analysis,” Nature Methods 9 (2012): 671–675, 10.1038/nmeth.2089. 22930834 PMC5554542

[38] G. W. Gray and D. G. McDonnell , “The Relationship between Helical Twist Sense, Absolute Configuration and Molecular Structure for Non‐Sterol Cholesteric Liquid Crystals,” Molecular Crystals & Liquid Crystals 34 (1976): 211–217, 10.1080/15421407708083708.

[39] J. W. Goodby , A. J. Slaney , C. J. Booth , et al., “Chirality and Frustration in Ordered Fluids,” Molecular Crystals and Liquid Crystals 243 (1994): 231–298, 10.1080/10587259408037771.

[40] A. Sharma , T. Mori , H. C. Lee , M. Worden , E. Bidwell , and T. Hegmann , “Detecting, Visualizing, and Measuring Gold Nanoparticle Chirality Using Helical Pitch Measurements in Nematic Liquid Crystal Phases,” ACS Nano 8 (2014): 11966–11976, 10.1021/nn504980w. 25383947

[41] T. Mori , A. Sharma , and T. Hegmann , “Significant Enhancement of the Chiral Correlation Length in Nematic Liquid Crystals by Gold Nanoparticle Surfaces Featuring Axially Chiral Binaphthyl Ligands,” ACS Nano 10 (2016): 1552–1564, 10.1021/acsnano.5b07164. 26735843

[42] A. Guerrero‐Martínez , B. Auguié , J. L. Alonso‐Gómez , et al., “Intense Optical Activity From Three‐Dimensional Chiral Ordering of Plasmonic Nanoantennas,” Angewandte Chemie International Edition 50 (2011): 5499–5503, 10.1002/anie.201007536. 21506211

[43] E. Grelet and M. M. C. Tortora , “Elucidating Chirality Transfer in Liquid Crystals of Viruses,” Nature Materials 23 (2024): 1282, 10.1038/s41563-024-01897-x. 38783105

[44] G. A. R. Rohaley and T. Hegmann , “Let's Twist Again,” Nature Materials 23 (2024): 1161–1163, 10.1038/s41563-024-01969-y. 39215157

[45] J. Stelzer , M. A. Bates , L. Longa , and G. R. Luckhurst , “Computer Simulation Studies of Anisotropic Systems. XXVII. The Direct Pair Correlation Function of the Gay–Berne Discotic Nematic and Estimates of Its Elastic Constants,” Journal of Chemical Physics 107 (1997): 7483–7492, 10.1063/1.474988.

[46] K. Singh and N. S. Pandey , “Elastic Constants of Discotic (Nematic) Liquid Crystals: Effect of Packing,” Liquid Crystals 25 (1998): 411–417, 10.1080/026782998206227.

[47] G. Heppke and D. Krüerke , “Nematic Discotic Liquid Crystals,” in Encyclopedia of Materials: Science and Technology, ed. K. H. J. Buschow , R. W. Cahn , M. C. Flemings , B. Ilschner , E. J. Kramer , S. Mahajan , and P. Veyssière , (Elsevier, 2001), 6046–6051.

[48] S. Dussi , S. Belli , R. van Roij , and M. Dijkstra , “Cholesterics of Colloidal Helices: Predicting the Macroscopic Pitch From the Particle Shape and Thermodynamic state,” Journal of Chemical Physics 142 (2015): 074905, 10.1063/1.4908162. 25702029

[49] E. Frezza , A. Ferrarini , H. B. Kolli , A. Giacometti , and G. Cinacchi , “Left or Right Cholesterics? A Matter of Helix Handedness and Curliness,” Physical Chemistry Chemical Physics 16 (2014): 16225–16232, 10.1039/C4CP01816H. 24969095

[50] H. B. Kolli , G. Cinacchi , A. Ferrarini , and A. Giacometti , “Chiral Self‐assembly of Helical Particles,” Faraday Discussions 186 (2016): 171–186, 10.1039/C5FD00132C. 26767786

[51] A. Ferrarini , G. J. Moro , and P. L. Nordio , “A Shape Model for the Twisting Power of Chiral Solutes in Nematics,” Liquid Crystals 19 (1995): 397–399, 10.1080/02678299508031997.

[52] D. P. N. Gonçalves and T. Hegmann , “Chirality Transfer From an Innately Chiral Nanocrystal Core to a Nematic Liquid Crystal: Surface‐Modified Cellulose Nanocrystals,” Angewandte Chemie International Edition 60 (2021): 17344–17349, 10.1002/anie.202105357. 33949085

[53] A. Visheratina , P. Kumar , and N. Kotov , “Engineering of Inorganic Nanostructures With Hierarchy of Chiral Geometries at Multiple Scales,” AIChE Journal 68 (2022): e17438, 10.1002/aic.17438.

[54] W. Ma , L. G. Xu , A. F. de Moura , et al., “Chiral Inorganic Nanostructures,” Chemical Reviews 117 (2017): 8041–8093, 10.1021/acs.chemrev.6b00755. 28426196

[55] H. H. Cao , E. Yang , Y. Kim , Y. Zhao , and W. Ma , “Biomimetic Chiral Nanomaterials With Selective Catalysis Activity,” Advancement of Science 11 (2024): 2306979, 10.1002/advs.202306979. PMC11187969 38561968

[56] S. R. Beeram , E. Rodriguez , S. Doddavenkatanna , et al., “Nanomaterials as Stationary Phases and Supports in Liquid Chromatography,” Electrophoresis 38 (2017): 2498–2512, 10.1002/elps.201700168. 28762520

[57] Y. Niihori , Y. Kikuchi , A. Kato , M. Matsuzaki , and Y. Negishi , “Understanding Ligand‐Exchange Reactions on Thiolate‐Protected Gold Clusters by Probing Isomer Distributions Using Reversed‐Phase High‐Performance Liquid Chromatography,” ACS Nano 9 (2015): 9347–9356, 10.1021/acsnano.5b03435. 26168308

[58] R. Breslow and Z. L. Cheng , “On the Origin of Terrestrial Homochirality for Nucleosides and Amino Acids,” Proceedings National Academy of Science USA 106 (2009): 9144–9146, 10.1073/pnas.0904350106. PMC2695116 19478058

[59] S. Im , S. Mousavi , Y.‐S. Chen , and Y. Zhao , “Perspectives of Chiral Nanophotonics: From Mechanisms to Biomedical Applications,” NPG Nanophotonics 1 (2024): 46, 10.1038/s44310-024-00045-2.

[60] S. H. Lee , C. H. Fan , A. Movsesyan , et al., “Unraveling the Chirality Transfer From Circularly Polarized Light to Single Plasmonic Nanoparticles,” Angewandte Chemie International Edition 63 (2024): e202319920, 10.1002/anie.202319920. 38236010

[61] W. Ma , F. Cheng , and Y. M. Liu , “Deep‐Learning‐Enabled on‐Demand Design of Chiral Metamaterials,” ACS Nano 12 (2018): 6326–6334, 10.1021/acsnano.8b03569. 29856595

[62] S. Y. Zhang , D. Fairen‐Jimenez , and M. J. Zaworotko , “Structural Elucidation of the Mechanism of Molecular Recognition in Chiral Crystalline Sponges,” Angewandte Chemie International Edition 59 (2020): 17600–17606, 10.1002/anie.202006438. 32589318 PMC7540565

[63] X. Feng , W. Pisula , L. Zhi , M. Takase , and K. Müllen , “Controlling the Columnar Orientation of C_3_ ‐Symmetric “Superbenzenes” Through Alternating Polar/Apolar Substitutents,” Angewandte Chemie International Edition 47 (2008): 1703–1706, 10.1002/anie.200703967. 18203222

[64] X. Feng , W. Pisula , T. Kudernac , et al., “Controlled Self‐Assembly of C_3_ ‐Symmetric Hexa‐ peri ‐hexabenzocoronenes With Alternating Hydrophilic and Hydrophobic Substituents in Solution, in the Bulk, and on a Surface,” Journal of the American Chemical Society 131 (2009): 4439–4448, 10.1021/ja808979t. 19271704

[65] S.‐C. Chien , H.‐H. Chen , H.‐C. Chen , et al., “Stable, Low‐Temperature Discotic Nematic Superstructures by Incorporating a Laterally Substituted Sidearm in Hexakis(phenylethynyl)benzene Discogens,” Advanced Functional Materials 17 (2007): 1896–1902, 10.1002/adfm.200601019.

[66] M. Gupta , S. S. Mohapatra , S. Dhara , and S. K. Pal , “Supramolecular Self‐assembly of Thiol Functionalized Pentaalkynylbenzene‐decorated Gold Nanoparticles Exhibiting a Room Temperature Discotic Nematic Liquid Crystal Phase,” Journal of Materials Chemistry C 6 (2018): 2303–2310, 10.1039/C7TC05444K.

[67] H. Yu and G. H. Mehl , “A Facile Synthesis of a Room‐temperature Chiral Discotic Nematic Liquid Crystal Based on Pentaalkynylbenzene Core,” Liquid Crystals 48 (2021): 1750–1757, 10.1080/02678292.2021.1900433.

[68] X. Ye , C. Zheng , J. Chen , Y. Gao , and C. B. Murray , “Using Binary Surfactant Mixtures To Simultaneously Improve the Dimensional Tunability and Monodispersity in the Seeded Growth of Gold Nanorods,” Nano Letters 13 (2013): 765–771, 10.1021/nl304478h. 23286198

[69] L. Scarabelli , M. Coronado‐Puchau , J. J. Giner‐Casares , J. Langer , and L. M. Liz‐Marzán , “Monodisperse Gold Nanotriangles: Size Control, Large‐scale Self‐assembly, and Performance in Surface‐enhanced Raman Scattering,” ACS Nano 8 (2014): 5833–5842, 10.1021/nn500727w. 24848669

[70] Y. Li , D. Yu , L. Dai , A. Urbas , and Q. Li , “Organo‐Soluble Chiral Thiol‐Monolayer‐Protected Gold Nanorods,” Langmuir 27 (2011): 98–103, 10.1021/la104131y. 21142010

[71] F. Qin , T. Zhao , R. Jiang , et al., “Thickness Control Produces Gold Nanoplates With Their Plasmon in the Visible and Near‐Infrared Regions,” Advanced Optical Materials 4 (2016): 76–85, 10.1002/adom.201500496.

