# Supplementary material for: The Role of Shape Commensurability in Chirality Transfer: Gold Nanoshape Solutes in a Discotic Nematic Liquid Crystal Solvent
Source: Angew Chem Int Ed Engl. 2026 May 14;65(27):e1907246. doi: 10.1002/anie.1907246 (PMC13327582; doi:10.1002/anie.1907246)
Supplement: Supplementary file 1 — The authors have cited additional references within the Supporting Information [63, 64, 65, 66, 67, 68, 69, 70, 71]. Supporting File: anie72702‐sup‐0001‐SuppMat.pdf. [file ANIE-65-e1907246-s001.pdf]

Supporting Information
©Wiley-VCH 2025
69451 Weinheim, Germany

The Role of Shape Commensurability in Chirality Transfer: Gold Nanoshape Solutes in a Discotic Nematic Liquid Crystal Solvent

Gourab Acharjee, Lara Querciagrossa, Grace A. R. Rohaley, Nicholas M. Kamuti, Ashwathanarayana Gowda, Suraj Kumar Pathak, Asmita Shah, Kun Zhang, Jianfang Wang, Claudio Zannoni, Torsten Hegmann*

Abstract: Chirality, as an inherently geometric concept, is well understood at most length scales and a principal attribute of objects and figures. Quantitative models predicting the efficacy of the transmission of chirality across length scales have only recently begun to emerge. We here provide further proof-of concept data and calculations for a modus operandi for nanoshape solutes featuring a chiral ligand shell in an achiral discotic nematic (N_D) liquid crystal solvent showing that chirality transfer may be understood by remarkably simple geometric considerations. This mechanism is based on the product of a pseudoscalar chirality indicator and a geometric shape compatibility factor based on the two-dimensional isoperimetric quotients for nanoshape solutes and N_D molecule. The model is tested on an experimental set of precisely engineered gold nanoshapes, rods, prisms, and discs, that validates that shape commensurability between nanoscale solute and nematic solvent is a prerequisite for efficacious chirality transfer as determined by the helical twisting power of the nanoshapes in the induced chiral N_D* phase. Thus, we predict that libraries of calculated and in-parallel acquired experimental data among related nanoshapes and even small organic molecules pave the way for predictive calculations of chirality transfer in nanoscale, macromolecular, biological, and small molecule systems.

DOI:

Table of Contents

Table of Contents ... 2
S1. Materials and Methods ... 4
S1.1. Basic Synthesis Equipment ... 4
S2. Procedures ... 5
S2.1. Synthesis of 4-dodecyl-4'-ethynylbipheny[35] (Intermediate-A, 5): ... 5
S2.1.1 Step-I: Synthesis of 4'-bromobiphenyl-4-yl)ethynyl)trimethylsilane, 2a ... 5
S2.1.2 Step-II: Synthesis of 4-dodecylbiphenyl-4'ethynyltrimethylsilane[36], 3a ... 6
S2.1.3 Step-III: Synthesis of 4-dodecyl-4'-ethynylbiphenyl (Intermediate-A, 5): ... 7
S2.2. Synthesis of room temperature discotic nematic (N_D) liquid crystal **1**: ... 9
S2.2.1. Method-I: Synthesis via pentalkynyl substituted benzene[15a,37] (8): ... 9
S2.2.2. Method-II: Modified synthesis of room-temperature discotic nematic LC **1** ... 11
S2.2.2.1: Step-I: Synthesis of monosubstituted intermediate-B ... 11
S2.2.2.2: Step-II: Synthesis of discotic nematic LC **1** ... 13
S2.3. Synthesis of (*R*)-C3/T8-SAc chiral solute ... 15
S2.3.1. Synthesis of pentabromo-C8 intermediate[38,39] (13): ... 15
S2.3.2. Synthesis of pentabromo-C8 thioacetate[38] (15): ... 16
S2.3.3. Synthesis of (*R*)-C3 chiral intermediate step-I: ... 18
S2.3.4. Synthesis of (*R*)-C3 chiral intermediate[16] step-II: ... 18
S2.3.5. Synthesis of (*R*)-C3 chiral intermediate step-III: ... 19
S2.3.6. Synthesis of (*R*)-C3 chiral intermediate step-IV: ... 20
S2.3.7. Synthesis of (*R*)-C3/T8-SAc thioacetate (23): ... 22
S2.4. Synthesis of (*S*)-C1/T8-SAc chiral solute ... 23
S2.4.1. Synthesis of (*S*)-C1 chiral intermediate step-I: ... 23
S2.4.2. Synthesis of (*S*)-C1 chiral intermediate[14] step-II ... 24
S2.4.3. Synthesis of (*S*)-C1 chiral intermediate step-III ... 26
S2.4.4. Synthesis of (*S*)-C1/T8-SAc thioacetate ... 27
S2.5. Synthesis of (*S*)-C1/T12-SAc chiral solute and (*S*)-C1/T12-SH ligand ... 29
S2.5.1. Synthesis of pentabromo-C12 intermediate (35): ... 29
S2.5.2. Synthesis of pentabromo-C12 thioacetate (36): ... 31
S2.5.3. Synthesis of (*S*)-C1/T12-SAc thioacetate (37): ... 32
S2.5.3. Synthesis of (*S*)-C1/T12-SH thiol (38): ... 34
S2.6. Synthesis of (*R*)-C1/T12-SAc chiral solute ... 36
S2.6.1. Synthesis of (*R*)-C1 chiral intermediate step-I: ... 36
S2.6.2. Synthesis of (*R*)-C1 chiral intermediate step-II ... 38
S2.6.3. Synthesis of (*R*)-C1 chiral intermediate step-III ... 39
S2.6.4. Synthesis of (*R*)-C1/T12-SAc thioacetate (45): ... 41
S2.6.5. Synthesis of (*R*)-C1/T12-SH thiol (46): ... 42
S3. X-ray diffraction (XRD) data of N_D phase ... 45
S4. Calculations of IPR_{2D} using molecular envelope for **1** and the chiral organic solutes ... 46
S5. Synthesis of gold nanoshapes ... 47
S5.1. Synthesis of gold nanorods (GNRs)[40] ... 47
S.4.1.1. TEM images of GNRs ... 48
S.5.2. Synthesis of gold nanoprisms (GNPRs)[41] ... 48
S5.2.1. TEM images of GNPRs ... 50

S5.3. Synthesis of gold nanodiscs (GNDs)[18c] 51
S5.3.1. TEM images of GNDs 52
S6. Surface functionalization of gold nanoshapes 53
S6.1 UV-vis and TEM 53
S6.2 ^{1}H-NMR spectroscopy 54
S6.3 Thermogravimetric analysis (TGA) of (*S*)-C1/T12-S−capped gold nanoshapes & calculations of surface coverage 55
S7. N_D*-LC phase induced by chiral free ligands 56
S7.1. Polarized optical microscopy images of induced N_D*-LC phase 56
S7.2 Twist direction of induced N_D*-LC phase depending on position and configuration of chiral center for the chiral ligands (Gray-McDonnell Rules) 57
S7.3 Thin film induced circular dichroism (ICD) spectropolarimetry of induced N_D*-LC phase and solution CD 58
S8. Polarized optical microscopy images of the N_D*-LC phase obtained by admixing gold nanoshapes 59
S9. S^{XN} values for ST, RP, and RH shapes 63
S10. Plots of inverse pitch vs. mole fraction: calculation of $|\beta_{mol}|$ 63
S11. Quantification of chirality transfer by geometrical chirality model 63
S11.1 Atomistic and coarse-grained ligand structures 63
S11.2 Gold nanoshapes for ligand-shell 64
S11.3 Chiral ligands 64
S11.4 Ligand decorated nanoshapes[8b,12a] 64
S11.5 Values of IPR_{2D}^{X}, S_{mol}^{XN}, and $|G_{oa,max}^{a} S_{mol}^{XN}|$ for chiral organic solutes and nanoshapes assuming rigid molecular envelope 65
S11.6 Plots of $|G_{oa,max}^{a} S^{XN}|$ or $|G_{oa,max}^{a} S_{mol}^{XN}|$ vs. $|\beta_{mol}|$ assuming RH and RP shapes as well as rigid molecular envelopes of **1** 66
S12. Variation of GND height (i.e., aspect ratio, *AR*) 67
S12.1 Synthesis and characterization of GND with *AR* ~ 5.4 ($GND_{AR\,\sim\,5.4}$) 67
S12.2 Polarized optical microscopy images of the N_D*-LC phase obtained by admixing GND with *AR* ~ 5.4 69
S12.3 Plots of $1/p$ vs. mole fraction and $|G_{oa,max}^{a} S^{XN}|$ vs. $|\beta_{mol}|$ for $GND_{AR\,\sim\,5.4}$ assuming a circular shape for **1** 70
Author Contributions 70

S1. Materials and Methods

All chemicals and solvents were purchased from commercial sources and used without further purification, except when otherwise noted. Hexadecyltrimethylammonium bromide (CTAB; CAS: 57-09-0), sodium oleate (NaOL; CAS: 143-19-1), hydrogen tetrachloroaurate trihydrate ($HAuCl_4{\cdot}3H_2O$; CAS: 16961-25-4), silver nitrate ($AgNO_3$; CAS: 7761-88-8), sodium borohydride ($NaBH_4$; CAS: 16940-66-2), L-ascorbic Acid (AA; CAS: 50-81-7), cetyltrimethylammonium chloride (CTAC; CAS: 112-02-7) were purchased from Sigma-Aldrich (USA). Hydrochloric acid (HCl) was purchased from Thermo Fisher Scientific, US. Pentabromophenol, 1-ethynyl-4-octylbenzene, copper iodide, bis(triphenylphosphine)-palladium (II) dichloride, 4-hydroxy-4'-iodobiphenyl, triphenylphosphine, XPhos-Pd-G3, XPhos, triethylamine, 1-bromooctanol, 1-bromododecanol, diisopropyl azodicarboxylate (DIAD), (*R*)-(+)-*β*-citronellol, potassium carbonate, potassium iodide, potassium thioacetate, methanol, butanone, dimethylformamide (DMF), and dichloromethane (DCM), tetrahydrofuran (THF) were all purchased from Sigma-Aldrich (USA). ^1H and ^{13}C NMR spectra were recorded in $CDCl_3$ on a Bruker AV 400 MHz NMR-spectrometer operating at 400 MHz (^1H), 101 MHz (^{13}C) and an Agilent 500 MHz NMR-spectrometer operating at 500 MHz (^1H) and 126 MHz (^{13}C). Chemical shifts are in δ units (ppm) with the residual solvent peak or TMS as the internal standard. The coupling constant (J) is reported in hertz (Hz). NMR splitting patterns are designated as follows: s, singlet; d, doublet; t, triplet; and m, multiplet. Column chromatographic separations were performed on silica gel (60 – 120, 100 – 200, and 230 – 400 mesh) or using a CombiFlash NextGen 300 Automated Flash Chromatography System by Teledyne ISCO with internal UV detector 200-400 nm and PeakTrak software at flow rates ranging from 1 to 300 mL min^{-1} and maximum pressure limit of 160 psi (11 bar) as well as active solvent level sensing. Columns used were RediSep Gold® silica gel disposable flash columns (particle size: 20 – 40 µm spherical; mesh size = 400 – 632; pore size: 60 Å, 4.0 g – 40.0 g; cartridge: RediSep® empty disposable sample load cartridges. Thin layer chromatography (TLC) was performed on aluminum sheets pre-coated with silica gel (Merck, Kieselgel 60, F254). Spots were rendered visible by exposing the plate to UV light. Microwave assisted synthesis were performed on Anton Parr Monowave 200 instrument. UV-vis spectra were measured by a Thermo Fisher evolution 220 spectrometer. High-resolution (HR) and gas chromatography (GC) mass spectrometry were performed at Indiana University's mass spectrometry laboratory using either a Thermo Fisher Scientific Q-Exactive Plus with ESI or APCI ionization operated at 70,000 resolving power or using a high-resolution GC-MS analysis instrument (Agilent 7890B/7250 GC-Quadrupole Time-of-Flight (QTof)). The data for the latter was acquired with 15 eV electron impact ionization. Samples were separated on a 20 meter DB-5 MS GC column (Agilent). TEM images were taken on a FEI Tecnai TF20 TEM instrument at an accelerating voltage of 200 kV. CD spectrophotometry used an OLIS 17 spectrophotometer, and thermogravimetric analysis a TGA Q500 (TA Instruments). Polarized light optical microscopy utilized an Olympus BX3 microscope equipped with an LTS 420E heating/cooling stage from Linkam Scientific Instruments. Variable-angle, temperature-controlled XRD experiments were performed using a Xenocs Xeuss 3.0 using a Cu Kα source (λ = 1.54 Å) equipped with a Linkam HFSX350 heating-cooling stage. Samples were sealed into X-ray diffraction glass capillaries (Charles Supper Co.). Admixing of the gold nanoshapes was achieved following a standard protocol[8b, 12a] as follows: precise quantities were weighted into rigorously cleaned glass vials using an ultramicrobalance. Standardized solutions of each nanoshape additive and N-LC host 5CB in a common organic solvent (purified $CHCl_3$) were prepared and the desired volumes of each solution were combined using calibrated Eppendorf pipettes and thoroughly mixed. Thereafter, the solvent was evaporated under a steady stream of nitrogen followed by mild vacuum. Homogeneous dispersions were ensured by mixing on a mechanical shaker at 240 rpm for 14 h at 25 °C and mild, pulsed sonication at 25 °C, that is, with the mixtures in the induced N_D*-LC phase. To measure p of the induced N_D*-LC phase, preparations with homeotropic anchoring conditions were used. Fingerprint textures were obtained using two cleaned glass substrates spin-coated with polyimide SE5661 and baked to provide strong homeotropic anchoring. In these preparations, the distances between dark striations under crossed polarizers were measured using the imaging software calibrated *via* a micro-ruler.

S1.1. Basic Synthesis Equipment

Round hotplate stirrer with corresponding temperature controller 6.81 in × 10.9 in, 100 – 1,400 rpm (Heidolph 036110519)
Disposable borosilicate vials with plastic caps (VWR, Cat.no.66011-143)
Micropipettes with disposable tips (ranges: 2 µL – 1,000 µL; Eppendorf)
PP Disposable microcentrifuge tubes, capacity 2 mL (VWR, cat.no.20170-170)
Glassware (Chem Glass)
PVC disposable plastic syringes (VWR, volume range: 1 – 10 mL)
Precleaned substrates, glass (Loveland, CO)
Ultrasonic bath (Branson 1510)
Digital orbital shaker by Southwest science, speed range 50-300 rpm in 10 rpm steps
Carbon-coated TEM grids (400 mesh, TED PELLA, Inc.) for sample preparation S1.2.

General preparation. All glassware was pre-cleaned and washed with *aqua regia* Including disposable glassware used as reaction vessels for the nanomaterial syntheses. All the samples were prepared using disposable glass pipettes. Moreover, no sonication or vortex spinning of any precursor solution was done during the nanomaterial synthesis; samples were mildly shaken and kept all solution capped before using them at a constant temperature. Every gold precursor solution was always freshly prepared. All ligand exchange reactions were performed under nitrogen purging.

S2. Procedures

S2.1. Synthesis of 4-dodecyl-4'-ethynylbipheny[35] (Intermediate-A, 5):

S2.1.1 Step-I: Synthesis of 4'-bromobiphenyl-4-yl)ethynyl)trimethylsilane, ***2a***

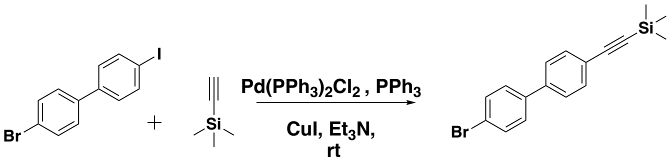

Experimental procedure:

To a stirring solution of 4-bromophenyl-4'-iodobenzene (5.0 g,13.93 mmol) in triethylamine (60 mL) were added $Pd(PPh_3)_2Cl_2$ (293.3 mg, 0.42 mmol), CuI (159.1 mg, 0.84 mmol) and triphenylphosphine (220.3 mg, 0.84 mmol) was degassed by bubbling through Ar for 40 min and then trimethylsilylacetylene (2.48 mL, 17.41 mmol) was added slowly at room temperature. The mixture was stirred at room temperature for 16 h. The reaction mixture was diluted with dichloromethane (50 mL) and filtered through celite on a fritted disk funnel. The filtrate was washed twice with 1M HCl (50 mL), twice with H_2O (200 mL), and dried over Na_2SO_4. The solvent was removed under vacuum, and the residue was purified by flash chromatography to obtain 4.16 g of pure compound **2a** as a white solid with 91% yield. ^1H NMR (400 MHz, $CDCl_3$) δ 7.64 – 7.43 (m, 8H, Ar-Biph-H), 0.33 – 0.23 (m, 9H,). ^{13}C NMR (101 MHz, $CDCl_3$) δ 95.40, 104.87, 122.10, 122.58, 126.79, 128.73, 132.10, 132.64, 139.35, 140.02. GC-MS: m/z calcd. for $C_{17}H_{17}BrSi$ $[M]^+$, $[M+H]^+$ = 328.0283, 330.0262; observed = 328.0282, 330.0262.

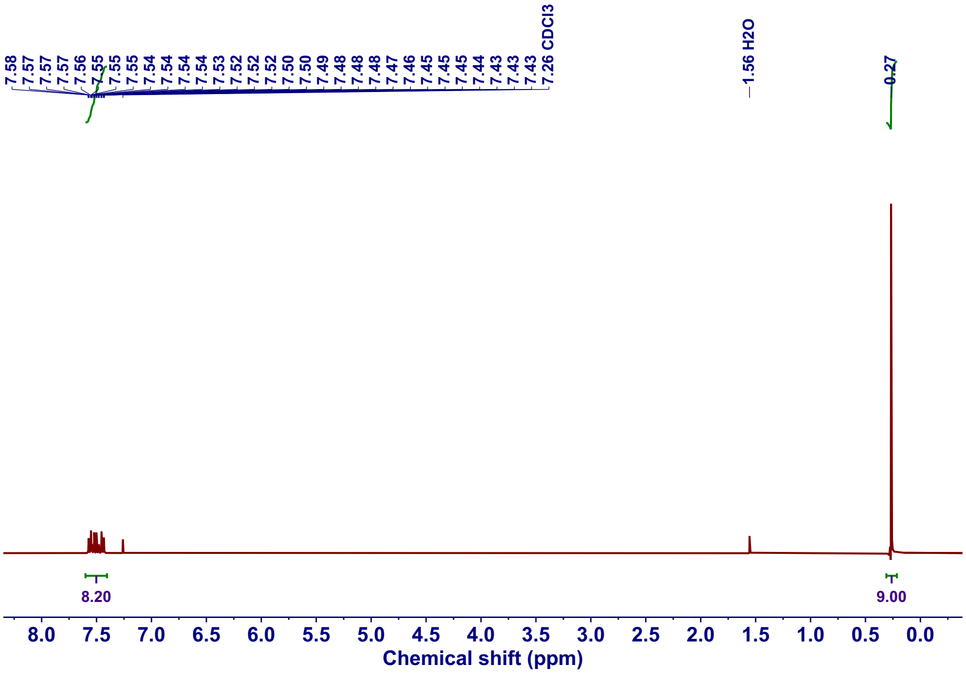

Figure S1a. ^1H-NMR spectrum of compound **2a**.

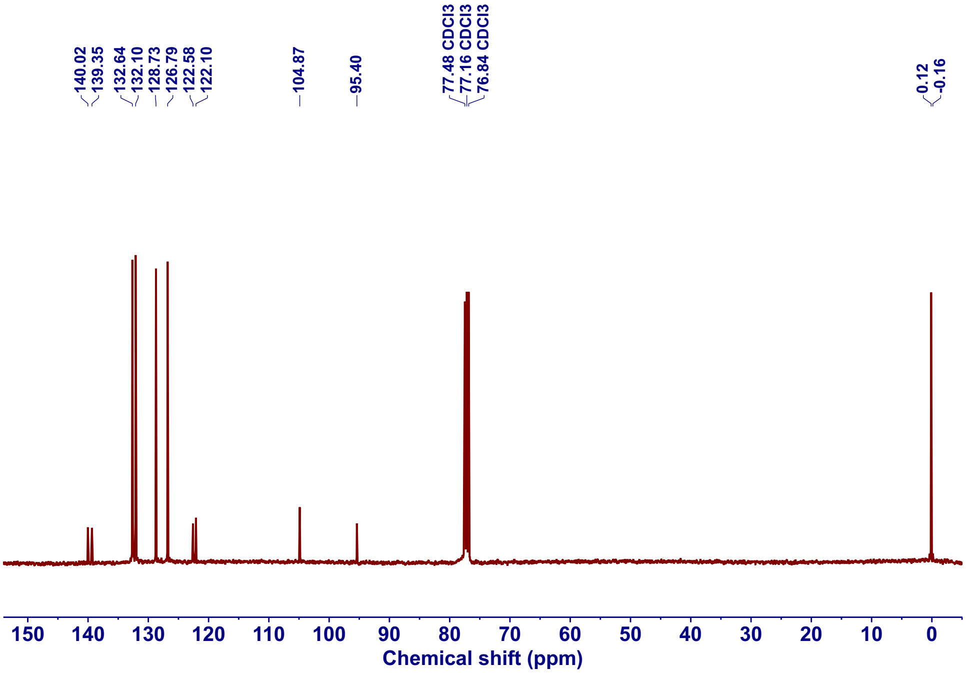

Figure S1b. ^{13}C -NMR spectrum of compound **2a**.

S2.1.2 Step-II: Synthesis of 4-dodecylbiphenyl-4'ethynyltrimethylsilane[36], ***3a***

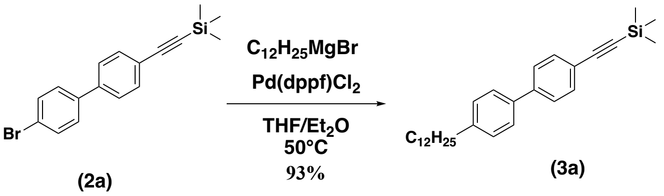

Experimental procedure:

To a stirring solution of 4'-bromobiphenyl-4-yl)ethynyl)trimethylsilane, **2a**, (7.0 g, 21.26 mmol) in 100.0 ml dry THF was added Pd(dppf)Cl_2 (0.75 g, 1.06 mmol). The mixture was degassed slowly by Ar, and then dodecylmagnesium bromide 32 mL (1M in diethyl ether) was added dropwise at 0 °C under Ar atmosphere. The mixture was stirred at 50 °C for 16 h. The reaction mixture was quenched by adding methanol and extracted with ethyl acetate. The organic layer was washed with water (200 mL) and brine (100 mL) and combined organic layer was dried over Na_2SO_4. The solvent was removed under vacuum, and the residue was purified by flash chromatography to obtain pure 8.3 g white solid product **3a** with 93% yield. ^{1}H NMR (400 MHz, $CDCl_3$) δ 7.57 – 7.47 (m, 6H, Ar-Biph-H), 7.29 – 7.21 (m, 2H, Ar-Biph-H), 2.68 – 2.60 (m, 2H), 1.70 – 1.58 (m, 2H), 1.37 – 1.28 (m, 7H), 1.26 (s, 11H), 0.93 – 0.85 (m, 3H), 0.27 (s, 9H). ^{13}C NMR (101 MHz, $CDCl_3$) δ 14.29, 22.85, 29.50, 29.52, 29.68, 29.75, 29.80, 29.83, 31.64, 32.08, 35.77, 94.79, 105.24, 121.76, 126.78, 126.97, 129.06, 132.49, 137.71, 141.27, 142.78. GC-MS: m/z calcd. for $C_{29}H_{42}Si$ $[M]^+$, $[M+H]^+$ = 418.3056, 419.3089; observed = 418.3056, 419.3079.

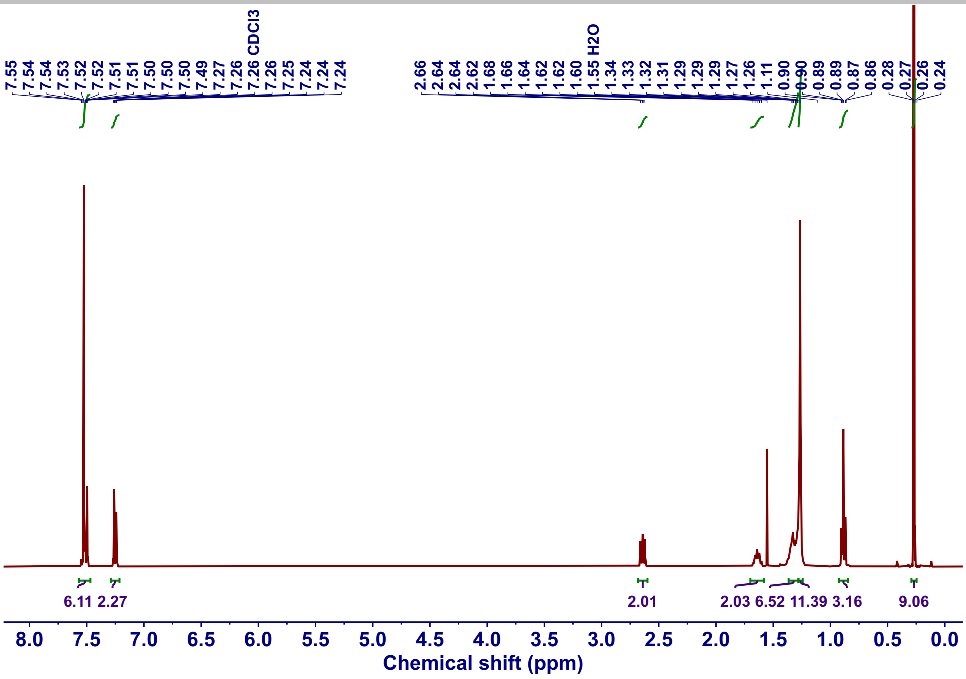

Figure S2a. ^{1}H-NMR spectrum of compound **3a**.

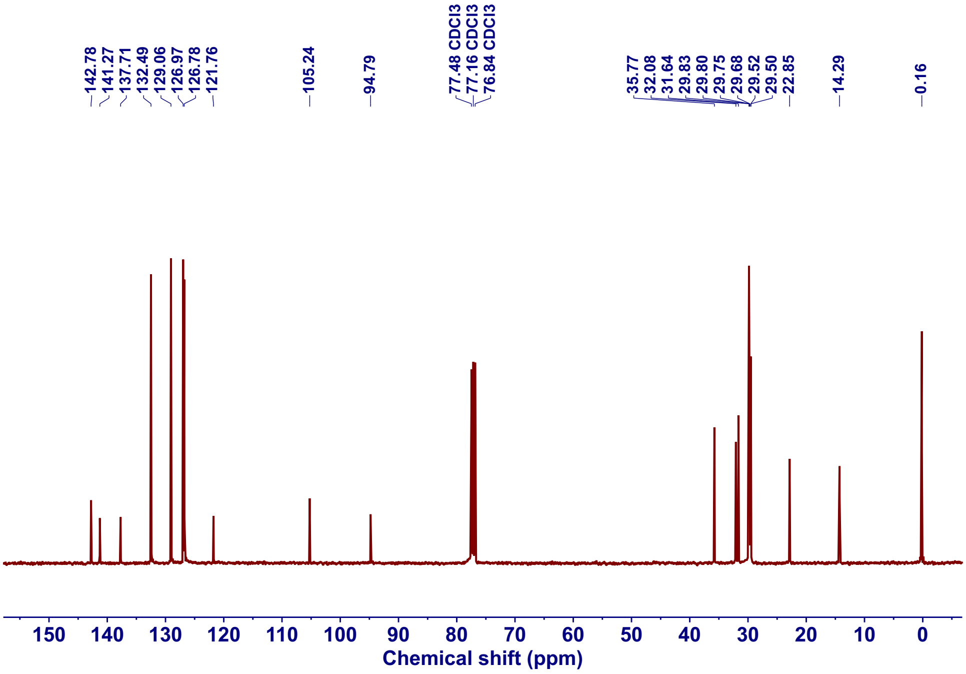

Figure S2b. ^{13}C -NMR spectrum of compound **3a**.

*S2.1.3 Step-III: Synthesis of 4-dodecyl-4'-ethynylbiphenyl (Intermediate-A, **5**):*

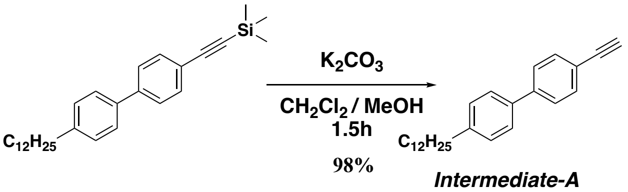

(3a)

(5)

Experimental procedure:

To a stirring solution of compound **3a** (7.58 g, 18.10 mmol) in dichloromethane (30 mL) was added K_2CO_3 (7.51 g, 54.31 mmol) and methanol (120 mL), the mixture was stirred at room temperature for 1.5 h. The reaction mixture was filtered, and filtrate was washed with water (200 mL)and extracted with dichloromethane (250 mL). The organic layer was washed further with brine (100 mL) and dried over Na_2SO_4. After solvent was removed under vacuum the crude was purified by flash chromatography to obtain 6.14 g pure Intermediate-A (**5**) with 98% yield. ^{1}H NMR (400 MHz, $CDCl_3$) δ 7.57 – 7.47 (m, 6H, ArH), 7.30 – 7.22 (m, 2H, ArH), 3.12 (s, 1H), 2.69 – 2.60 (m, 2H), 1.70 – 1.58 (m, 2H), 1.41 – 1.19 (m, 18H), 0.92 – 0.84 (m, 3H). ^{13}C NMR (101 MHz, $CDCl_3$) δ 14.16, 22.72, 29.38, 29.55, 29.62, 29.67, 29.70, 31.51, 31.95, 35.65, 76.62, 76.94, 77.25, 77.60, 83.67, 120.60, 126.81, 126.89, 128.97, 132.53, 137.53, 141.56, 142.75. GC-MS: m/z calcd. for $C_{26}H_{34}$ $[M]^+$, $[M+H]^+$ = 346.2661, 347.2694; observed = 346.2652, 347.2685.

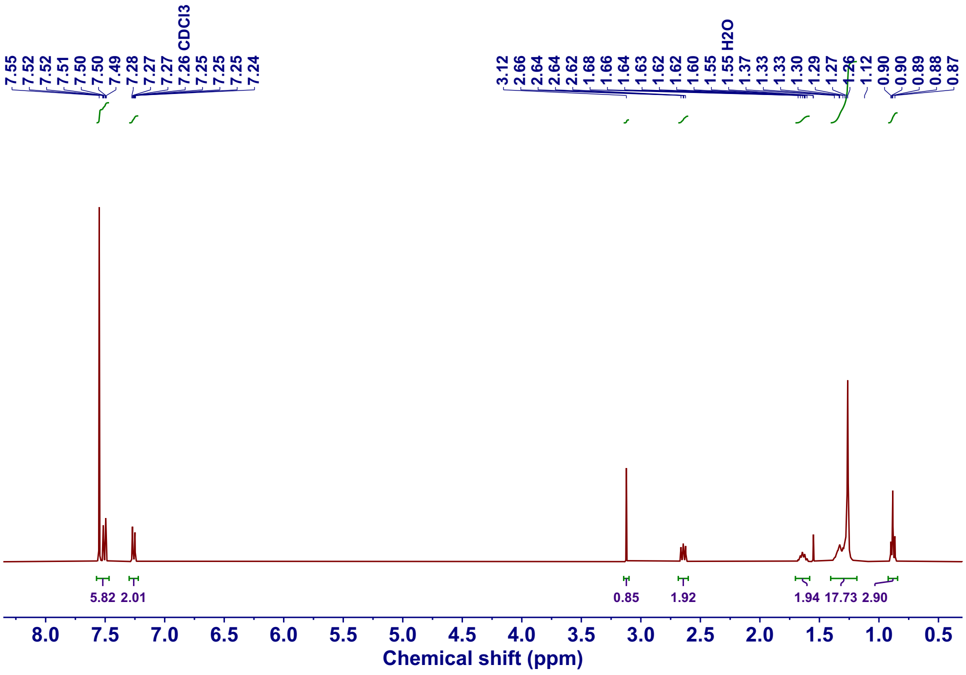

Figure S3a. ^{1}H-NMR spectrum of compound **5.**

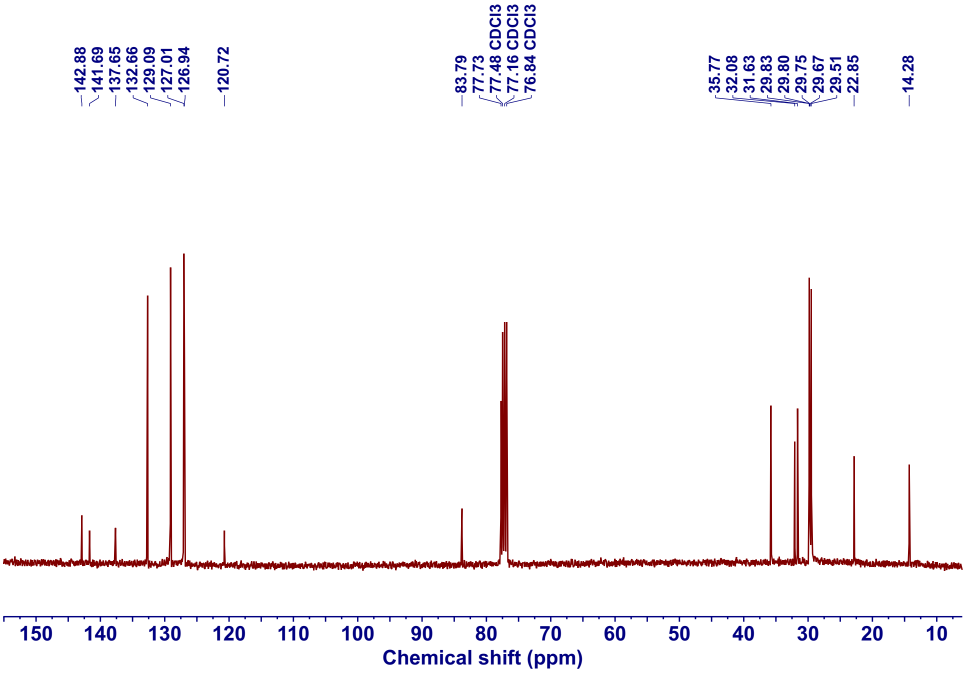

Figure S3b. ^{13}C-NMR spectrum of compound **5**

S2.2. Synthesis of room temperature discotic nematic (N_D) liquid crystal 1:

S2.2.1. Method-I: Synthesis via pentalkynyl substituted benzene[15a,37] ***(8)***:

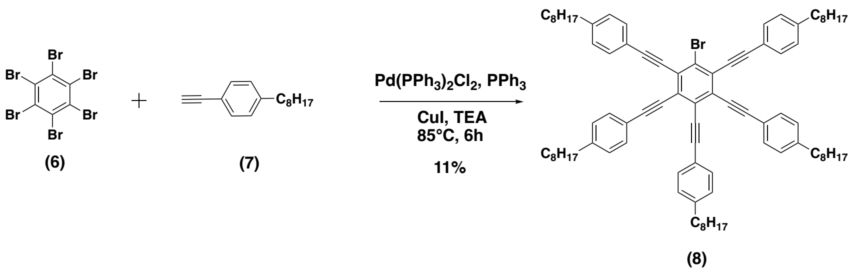

Experimental procedure:

To a mixture of *trans*-dichlorobis(triphenylphosphine)palladium(II) (63.64 mg, 0.09 mmol), copper iodide (34.53 mg, 0.18 mmol), hexabromobenzene (**6**) (500 mg, 0.91 mmol), and triethylamine (25 mL) was added 1-ethynyl-4-octylbenzene (1.19 g, 4.99 mmol) in triethylamine (15 mL) dropwise under a nitrogen atmosphere at reflux. The mixture was stirred at reflux for 15 h and then cooled. To the cooled reaction mixture was added EtOAc (100 mL). The mixture was washed twice with 1M HCl, twice with H_2O, and dried over $MgSO_4$. After removal of the solvents, the residue was purified by multiple rounds of flash column chromatography (1.0% hexane-EtOAc) to yield **8** as a yellow powder at 11% yield (120.0 mg). ^1H NMR (400 MHz, $CDCl_3$) δ 7.60 – 7.50 (m, 10H, ArH), 7.22 – 7.13 (m, 10H, ArH), 2.64 (td, J = 7.5, 1.6 Hz, 10H), 1.70 – 1.58 (m, 10H), 1.39 – 1.27 (m, 50H), 0.92 – 0.88 (m, 15H). ^{13}C NMR (101 MHz, $CDCl_3$) δ 1.17, 14.27, 22.84, 26.85, 29.44, 29.48, 29.64, 29.87, 30.32, 31.43, 32.05, 36.19, 86.64, 86.97, 87.21, 87.31, 87.55, 95.69, 98.55, 99.01, 99.63, 99.99, 100.16, 120.23, 120.32, 120.43, 120.55, 120.67, 125.34, 127.35, 127.40, 127.51, 127.59, 128.08, 128.69, 129.23, 131.85, 131.89, 131.94, 131.96, 144.09, 144.18, 144.33, 144.41. HRMS: m/z calcd. for $C_{86}H_{106}{}^{79}Br$, $C_{86}H_{106}{}^{81}Br$ $[M+H]^+$ = 1217.7472, 1219.7452; observed = 1217.7451, 1219.7457.

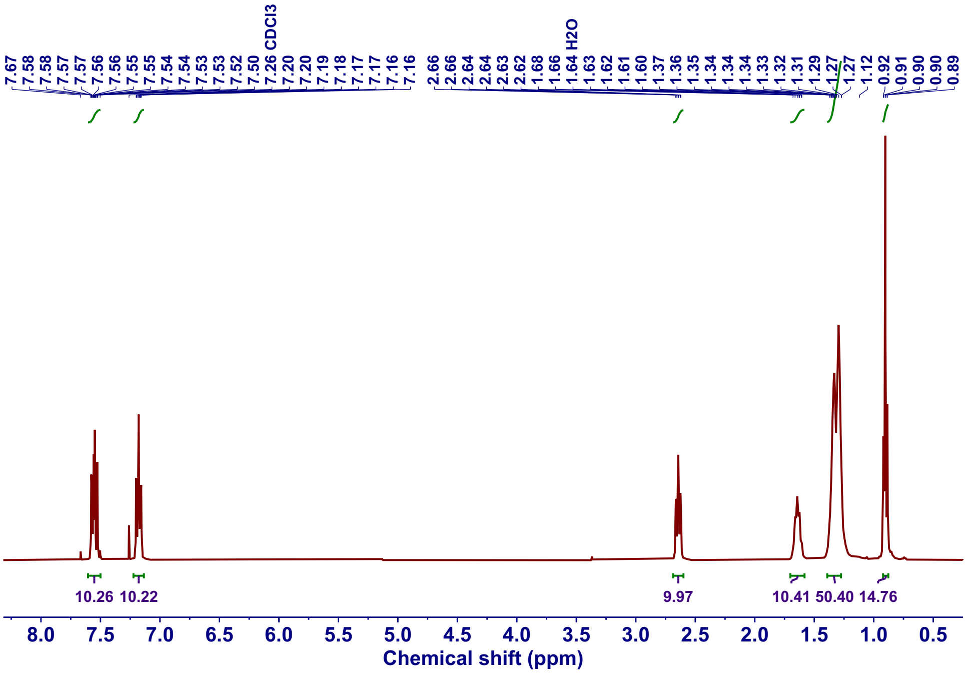

Figure S4a. ^1H-NMR spectrum of compound **8.**

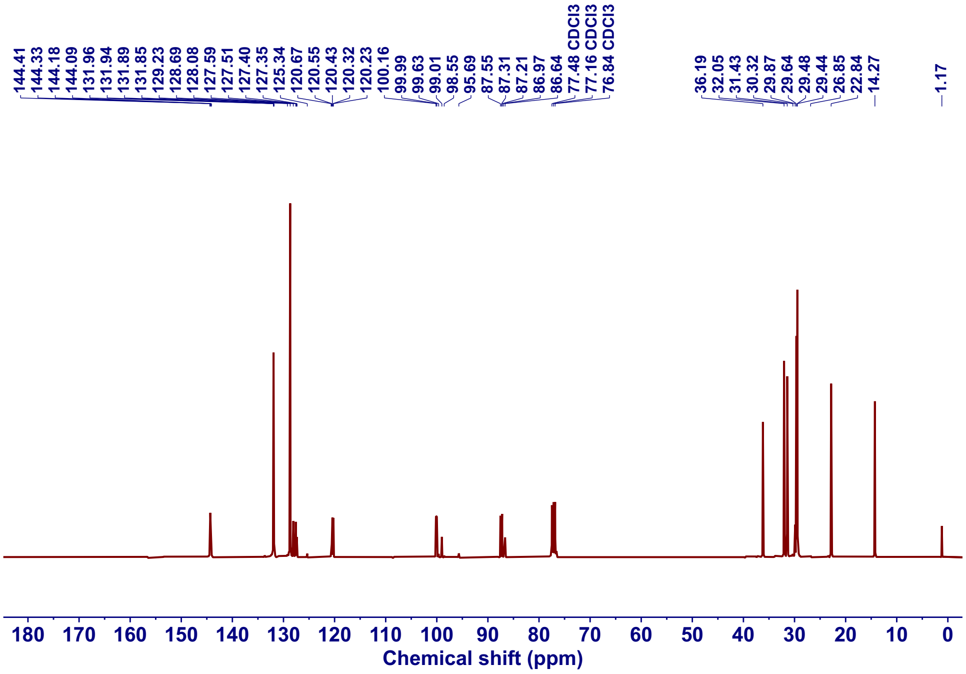

Figure S4b. ^{13}C-NMR spectrum of compound **8.**

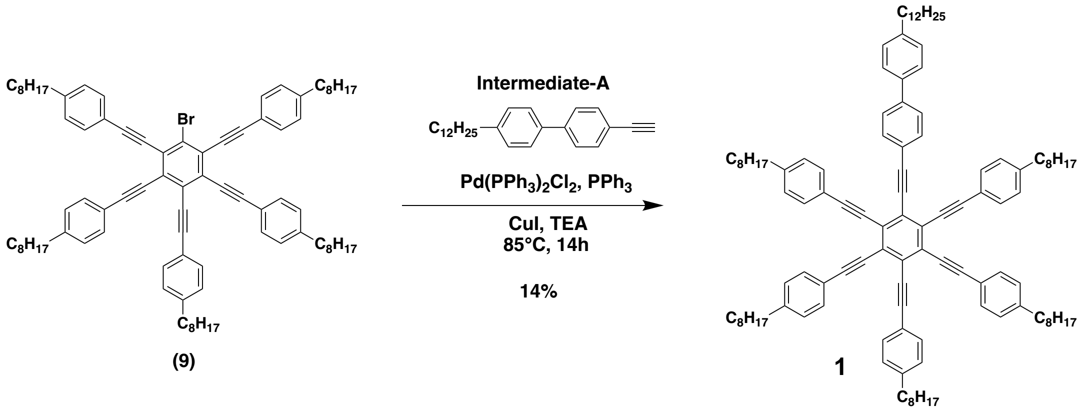

Experimental procedure:

To a mixture of *trans*-dichlorobis(triphenylphosphine)palladium(II) (20.0 mg, 0.021 mmol), copper iodide (8.0 mg, 0.04 mmol), compound **9** (170 mg, 0.14 mmol), and triethylamine (5.0 mL) was added 4-dodecyl-4'-ethynylbiphenyl (Intermediate-A, **5**) (70.0 mg, 0.21 mmol) in triethylamine (1.0 mL) dropwise under a nitrogen atmosphere at reflux. The mixture was stirred at reflux for 15 h and then cooled. To the cooled reaction mixture was added EtOAc (100 mL). The mixture was washed twice with 1M HCl, twice with H_2O, and dried over $MgSO_4$. After removal of the solvents, the residue was purified by multiple rounds of flash column chromatography (1% hexane-EtOAc) to yield 28 mg of **1** (14% yield). ^{1}H NMR (400 MHz, $CDCl_3$) δ 7.71 – 7.67 (m, 2H, Ar-Biph-H), 7.60 – 7.53 (m, 14H), 7.31 – 7.26 (m, 2H, Ar-Ph-H), 7.20 – 7.16 (m, 10H, Ar-Ph-H), 2.64 (t, *J* = 8.0 Hz, 12H), 1.65 (q, *J* = 7.4 Hz, 12H), 1.35 – 1.26 (m, 68H), 0.91 – 0.87 (m, 18H). ^{13}C NMR (101 MHz, $CDCl_3$) δ 1.18, 14.28, 22.83, 29.43, 29.48, 29.53, 29.64, 29.70, 29.77, 29.81, 29.84, 31.44, 31.66, 32.05, 32.08, 35.81, 36.20, 77.36, 87.16, 88.36, 99.20, 99.61, 99.67, 120.61, 122.04, 127.03, 127.25, 127.45, 127.50, 127.58, 128.70, 128.73, 129.10, 131.94, 132.39, 137.77, 141.54, 142.86, 144.20, 144.25. HRMS: m/z calcd. for $C_{111}{}^{13}CH_{139}$, $[M+H]^+$ = 1485.0905; observed = 1485.0900.

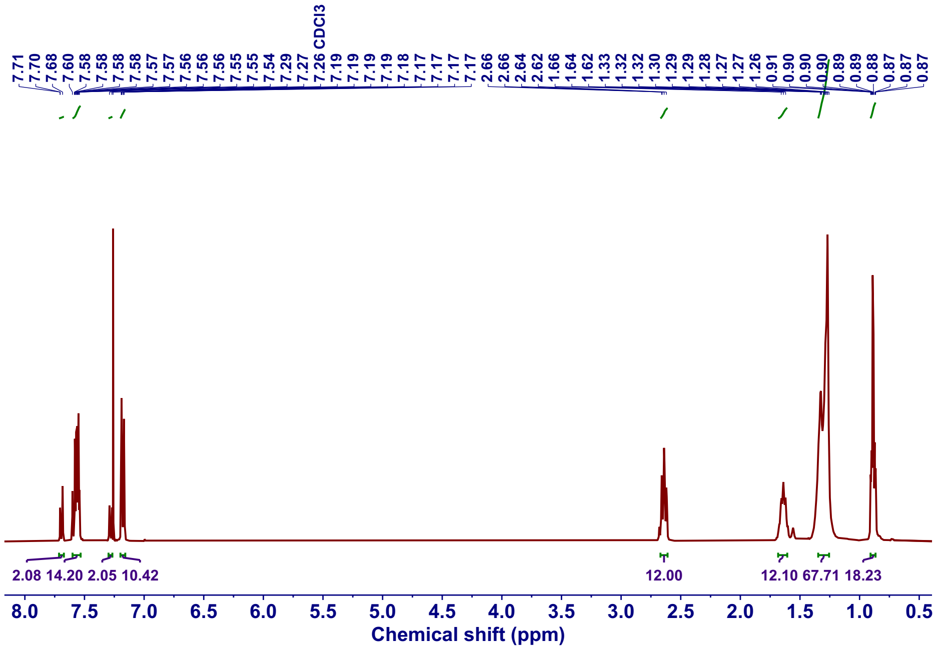

Figure S5a. ^{1}H-NMR spectrum of compound **1.**

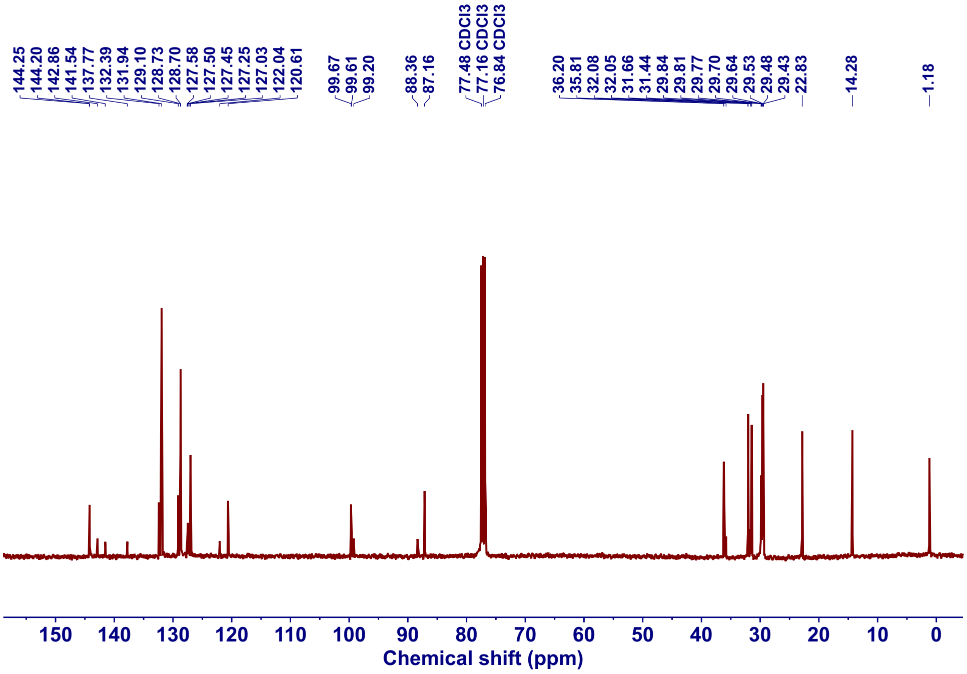

Figure S5b. ^{13}C-NMR spectrum of compound **1.**

S2.2.2. Method-II: Modified synthesis of room-temperature discotic nematic LC ***1***

S2.2.2.1: Step-I: Synthesis of monosubstituted intermediate-B

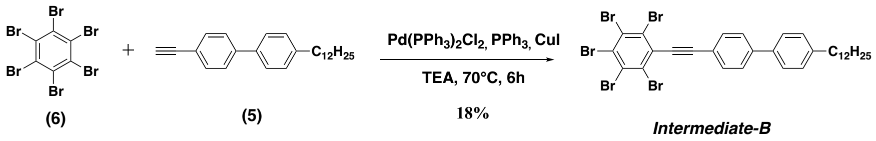

Experimental procedure:

To a mixture of *trans*-dichlorobis(triphenylphosphine)palladium(II) (36.7 mg, 0.05 mmol), triphenylphosphine (54.7 mg, 0.21 mmol) copper iodide (19.8 mg, 0.1 mmol), hexabromobenzene (**6**) (500.0 mg, 1.04 mmol), and triethylamine (15.0 mL) was added and degassed for 45 min with Ar. Followed by biphenyl scaffold (**5**) (400.0 mg, 1.16 mmol) in triethylamine (5 mL) dropwise under a nitrogen atmosphere. The reaction mixture was heated to 70°C and kept at this temperature for 5 h and then cooled. To the cooled reaction mixture was added dichloromethane (100 mL). The mixture was washed twice with 1M HCl (50 mL), twice with H_2O (200 mL), and dried over Na_2SO_4. After removal of the solvent the crude product was purified by flash chromatography and preparative TLC to obtain 140 mg of the pure product **Intermediate-B** with 18% yield. ^{1}H NMR (400 MHz, $CDCl_3$) δ 7.43 – 7.31 (m, 2H, Ar-Biph-H), 7.30 – 7.20 (m, 4H, Ar-Biph-H), 7.00 (t, *J* = 7.6 Hz, 2H, Ar-Biph-H), 2.38 (td, *J* = 7.7, 4.5 Hz, 2H), 1.38 (s, 2H), 1.01 – 0.99 (m, 14H), 0.60 (q, *J* = 8.8 Hz, 7H). ^{13}C NMR (101 MHz, $CDCl_3$) δ 14.12, 22.70, 29.37, 29.53, 29.61, 29.72, 31.47, 31.94, 35.64, 77.56, 119.98, 123.28, 126.33, 129.30, 132.31. HRMS: m/z calcd. for $C_{32}H_{33}(O_2)^{79}Br_3{}^{81}Br_2$, = 847.8359; observed = 847.8362.

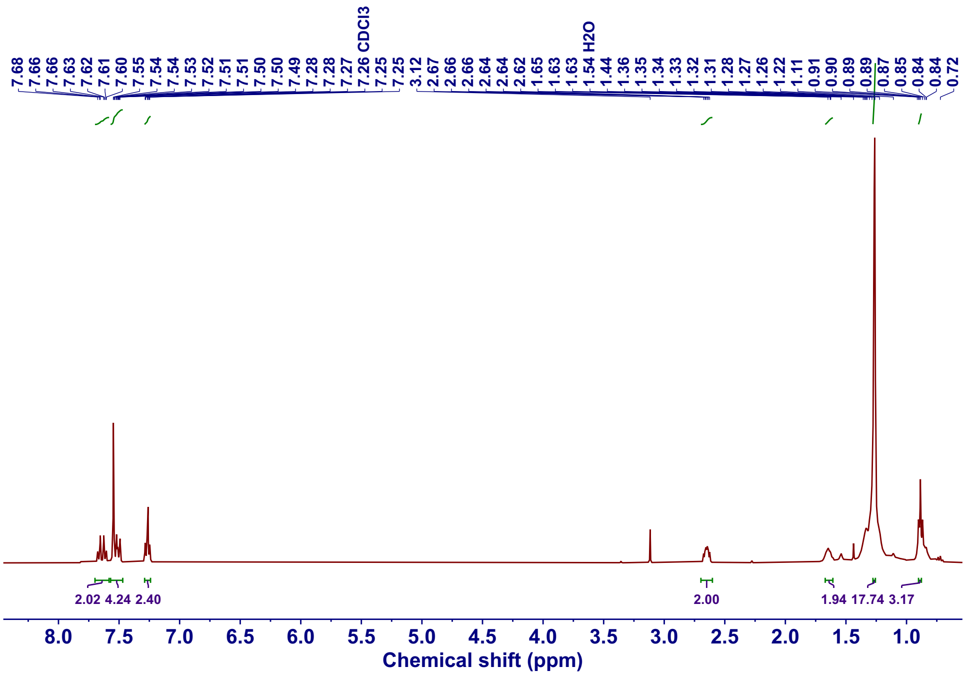

Figure S6a. ^{1}H-NMR spectrum of **Intermediate-B**.

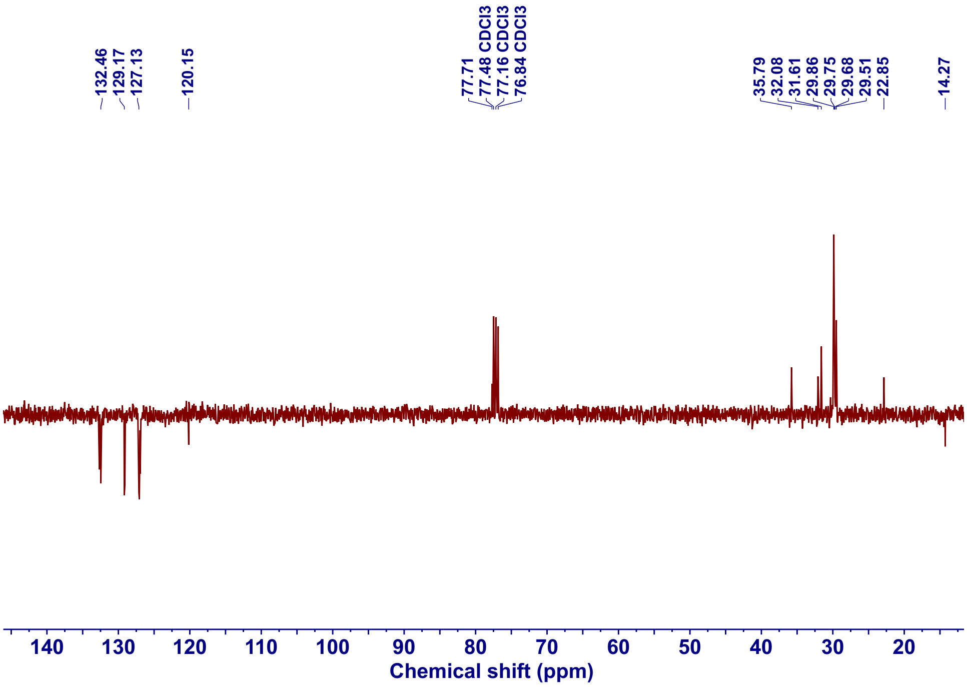

Figure S6b. ^{13}C-NMR spectrum of **Intermediate-B**.

S2.2.2.2: Step-II: Synthesis of discotic nematic LC **1**

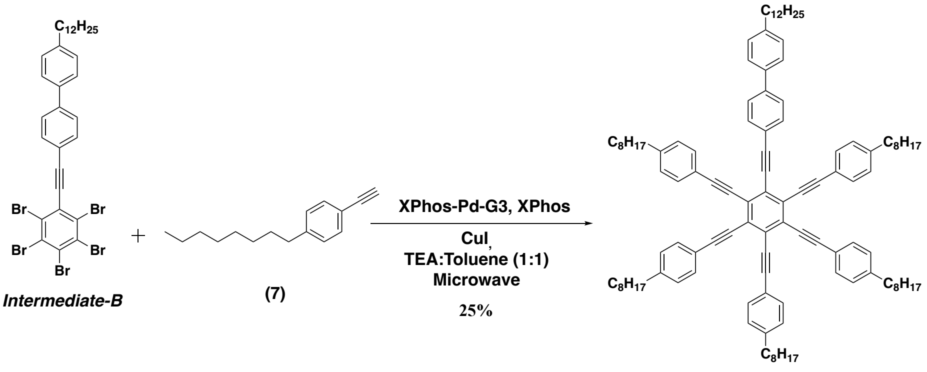

1

Experimental procedure:

To a mixture of X-PhosPd-G3 (31.1 mg, 0.04 mmol), copper iodide (13.9 mg, 0.073 mmol), XPhos (35.0 mg, 0.073 mmol) Intermediate-B (300.0 mg, 0.367 mmol), and triethylamine (3.0 mL) was added 1-ethynyl-4-octylbenzene, (**7**), (1.0 mL, 2.94 mmol) and toluene (3.0 mL) dropwise under a nitrogen atmosphere. The mixture was degassed for 1hr. then stirred at microwave synthesizer at 140 °C for 1h 40 mins. The reaction mixture was diluted with dichloromethane (10.0 ml) and filtered through celite on a fritted disk funnel. The filtrate was washed twice with 1M HCl (50.0 mL), twice with H_2O (100.0 mL), and dried over Na_2SO_4. After removal of the solvent the crude product was purified by flash chromatography and preparative TLC to obtain 135 mg of the pure product **1** with 25% yield. ^{1}H NMR (400 MHz, $CDCl_3$) δ 7.71 – 7.68 (m, 2H, Ar-BiPh-H), 7.60 – 7.55 (m, 14H), 7.29 – 7.27 (m, 2H, Ar-Ph-H), 7.19 – 7.16 (m, 10H, Ar-Ph-H), 2.64 (t, *J* = 7.2 Hz, 12H), 1.63 (d, *J* = 7.6 Hz, 12H), 1.29 (dd, *J* = 15.7, 6.5 Hz, 68H), 0.89 – 0.86 (m, 18H). ^{13}C NMR (101 MHz, $CDCl_3$) δ 1.18, 14.28, 22.83, 29.43, 29.48, 29.53, 29.64, 29.70, 29.77, 29.81, 29.84, 31.44, 31.66, 32.05, 32.08, 35.81, 36.20, 87.16, 88.36, 99.20, 99.61, 99.67, 120.61, 122.04, 127.03, 127.25, 127.45, 127.50, 127.58, 128.70, 128.73, 129.10, 131.94, 132.39, 137.77, 141.54, 142.86, 144.20, 144.25. HRMS: m/z calcd. for $C_{111}{}^{13}CH_{139}$, $[M+H]^+$ = 1485.09049; observed = 1485.0900.

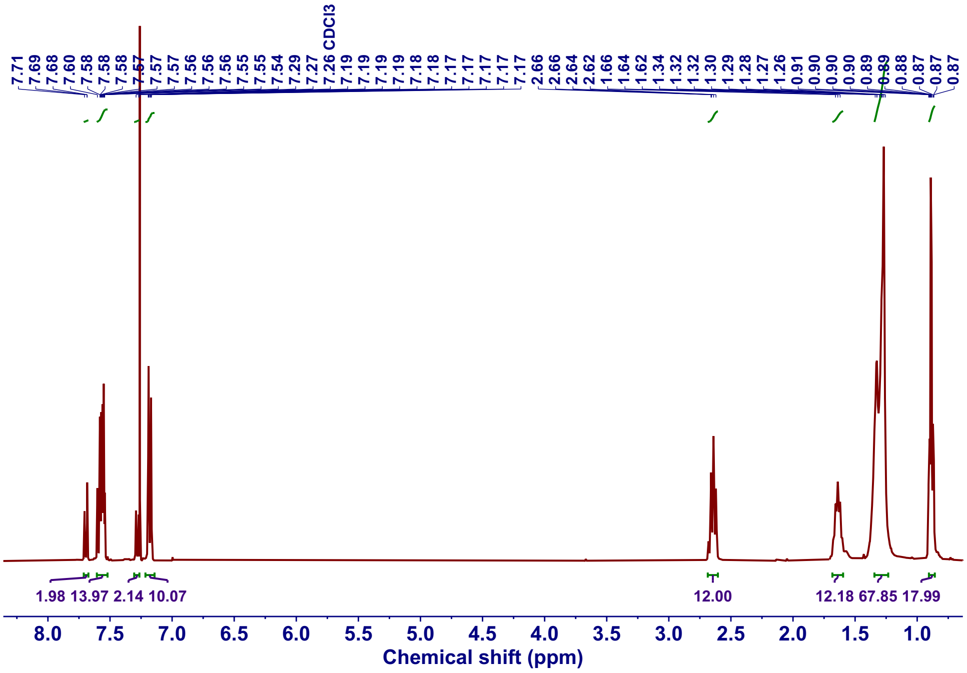

Figure S7a. ^{1}H-NMR spectrum of compound **1**.

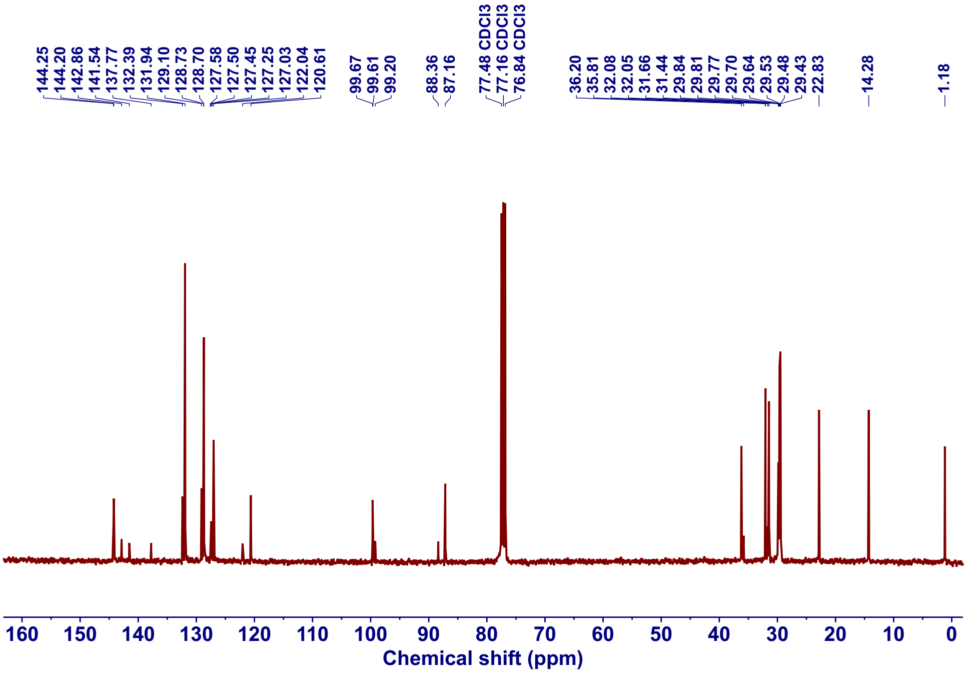

Figure S7b. ^{13}C-NMR spectrum of compound **1**.

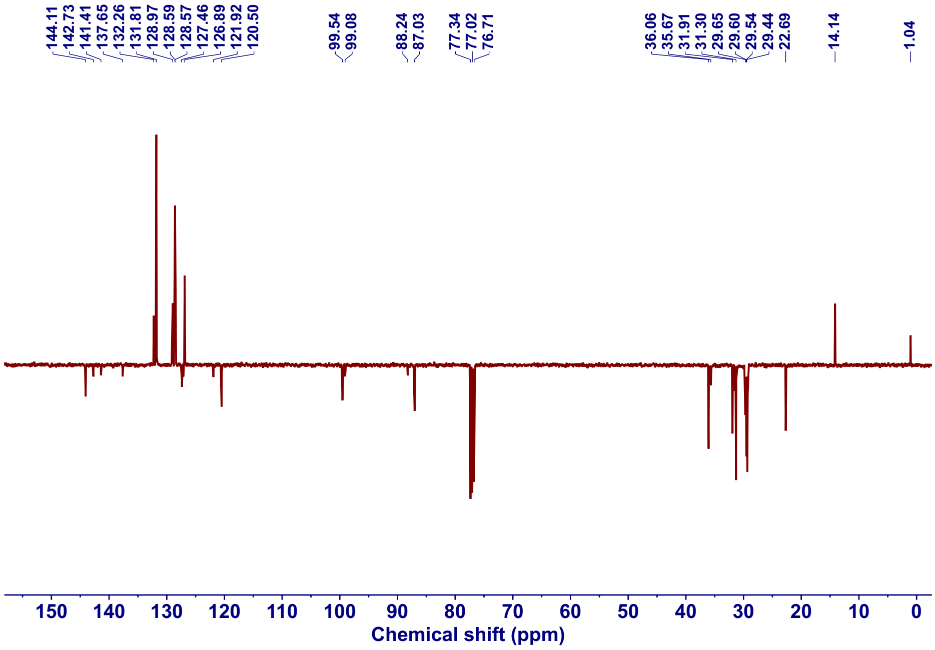

Figure S7c. ^{13}C-APT-NMR spectrum of compound **1**.

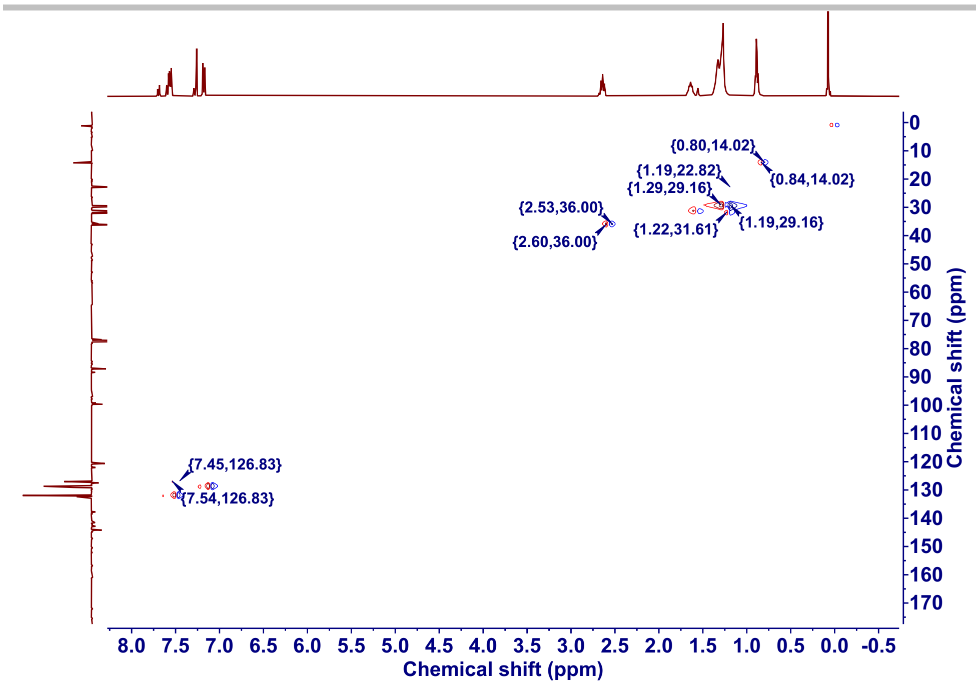

Figure S7d. {^{1}H -^{13}C}-HSQC NMR spectrum of compound **1**.

S2.3. Synthesis of (*R*)-C3/T8-SAc chiral solute

S2.3.1. Synthesis of pentabromo-C8 intermediate[38,39] ***(13)*:**

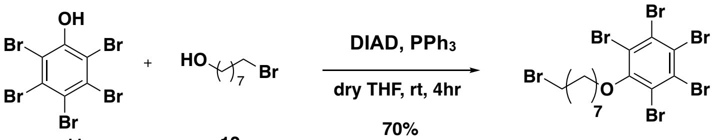

11 12 13

Experimental procedure:
To a stirring solution of pentabromophenol, **11** (2.0 g, 4.09 mmol) and 8-bromooctan-1-ol, 12 (1.03 g , 4.91 mmol) in dry THF (20.0 mL) was added DIAD (1.24 g, 6.14 mmol) dropwise and PPh_3 (1.61g, 6.14 mmol) at 0 °C. The reaction mixture was stirred at 0 °C for 4 h under Ar-atmosphere. The reaction was monitored by TLC and quenched with aqueous $NaHCO_3$ solution (100 mL) and extracted two times with EtOAc and the combined organic layer was dried with anhydrous Na_2SO_4. After removal of the solvent the crude product was purified by flash chromatography to obtain 1.84 g of the pure product (**13**) as white solid with 70% yield. ^{1}H NMR (400 MHz, $CDCl_3$) δ 3.98 (t, J = 6.5 Hz, 2H), 3.42 (t, J = 6.8 Hz, 2H), 1.93 – 1.81 (m, 4H), 1.59 – 1.29 (m, 8H). ^{13}C NMR (101 MHz, $CDCl_3$) δ 25.84, 25.99, 28.23, 28.80, 29.34, 29.95, 32.88, 34.15, 73.66, 122.01, 124.62, 128.48, 154.64.

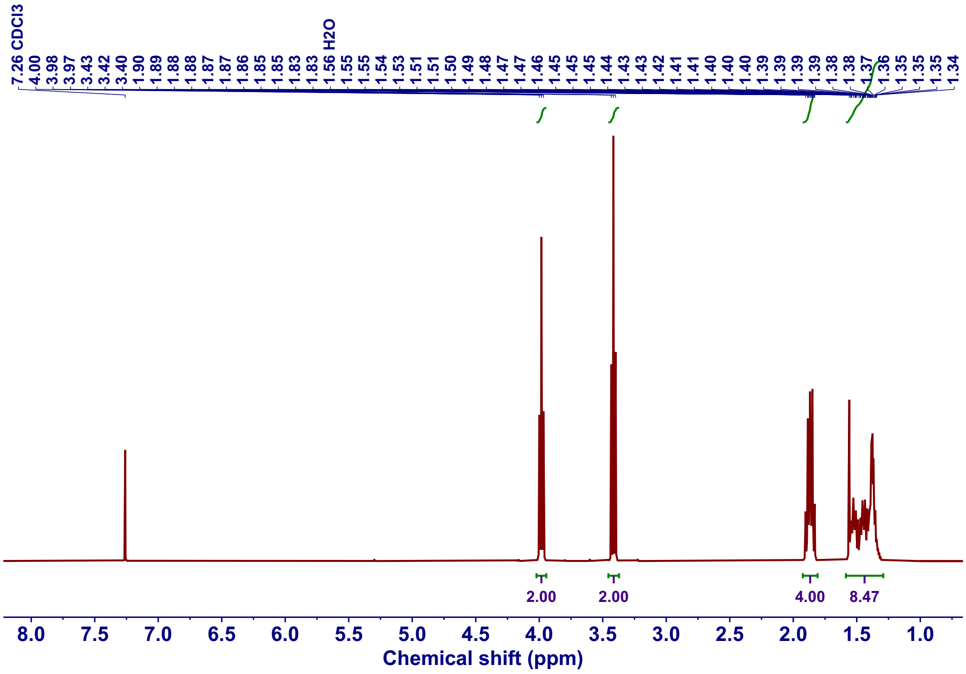

Figure S8a. ^{1}H-NMR spectrum of compound **13**.

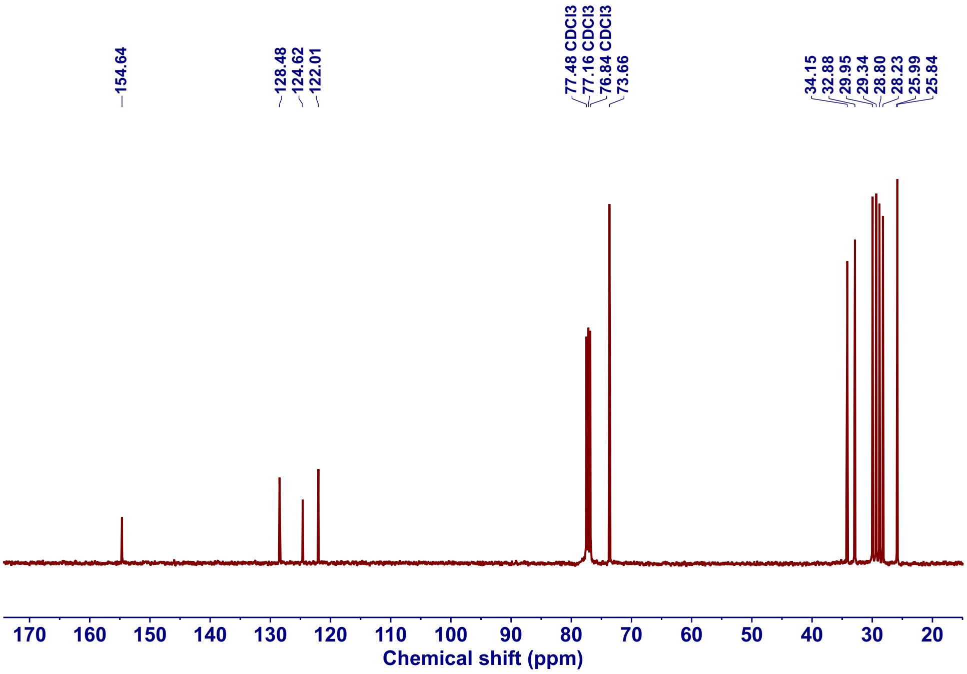

Figure S8b. ^{13}C-NMR spectrum of compound **13.**

S2.3.2. Synthesis of pentabromo-C8 thioacetate[38] *(**15**):*

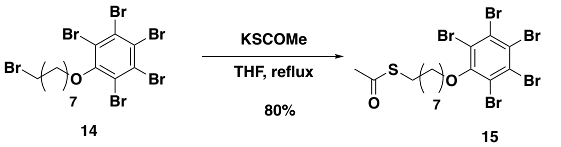

Experimental procedure:

To a stirring solution of compound **14** (1.50 g, 2.21 mmol) in dry THF (50.0 mL) was added potassium thioacetate (0.38 g, 3.31 mmol. The reaction mixture was refluxed for 16 h. The reaction was monitored by TLC and quenched with aqueous sodium bisulfite solution

(100 mL). The reaction mixture was washed additionally with water (100 mL), brine (100 mL) and extracted with EtOAc and the combined organic layer was dried with anhydrous Na_2SO_4. After removal of the solvent the crude product was purified by flash chromatography to obtain 1.2 g of the pure product (**15**) as white solid with 80% yield. ^{1}H NMR (400 MHz, $CDCl_3$) δ 4.01 (t, J = 6.5 Hz, 2H), 2.93 – 2.85 (m, 2H), 2.35 (s, 3H), 1.89 (dq, J = 8.6, 6.6 Hz, 2H), 1.61 – 1.53 (m, 4H), 1.46 – 1.31 (m, 6H). ^{13}C NMR (101 MHz, $CDCl_3$) δ 1.17, 25.86, 28.87, 29.15, 29.25, 29.37, 29.62, 29.96, 30.82, 73.72, 122.04, 124.61, 128.48, 154.69, 196.26. HRMS: m/z calcd. for $C_{16}H_{20}O_2{}^{79}Br_3{}^{81}Br_2{}^{32}S$, $C_{16}H_{20}O_2{}^{79}Br_2{}^{81}Br_3{}^{32}S$, $[M+H]^+$ = 674.70545, 676.70340; observed = 674.7043, 675.7025.

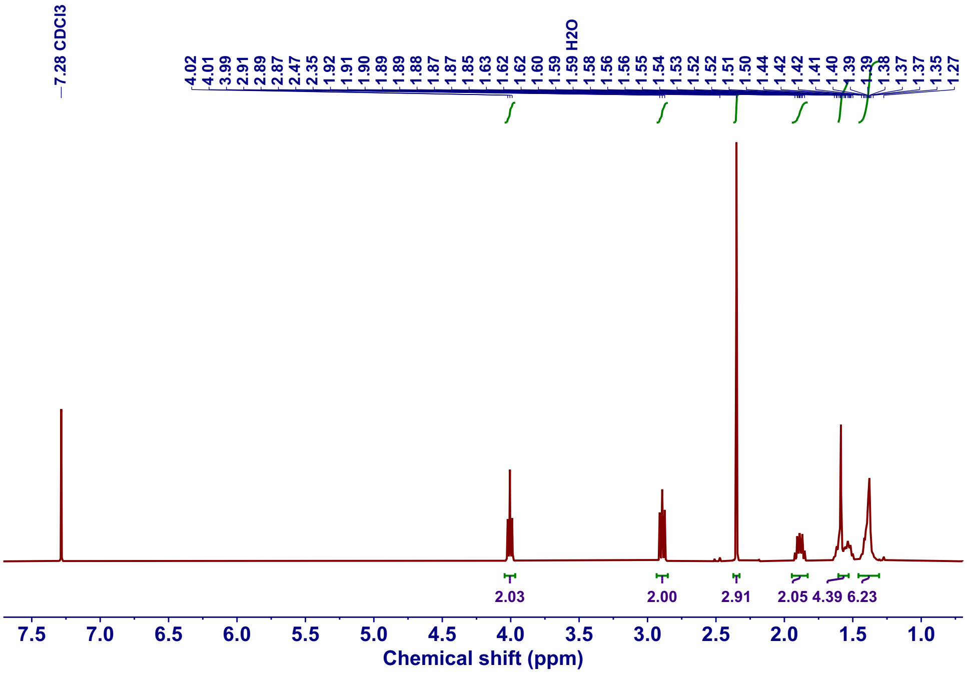

Figure S9a. ^{1}H-NMR spectrum of compound **15**.

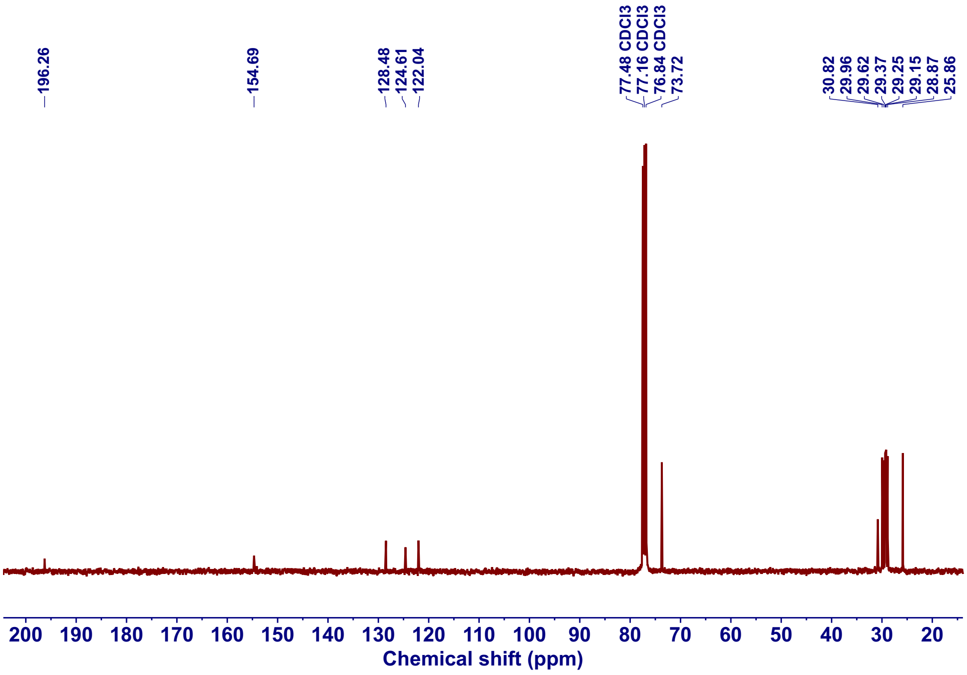

Figure S8b. ^{13}C-NMR spectrum of compound **15**.

S2.3.3. Synthesis of (R)-C3 chiral intermediate step-I:

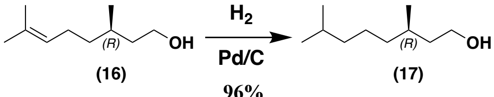

Experimental procedure:

To a stirring solution of (*R*)-(+)-*β*-citronellol (**16**) (2.0 g, 12.80 mmol) in EtOAc (50.0 mL) was added 5% Pd-C (0.2 g). The reaction mixture was stirred at r.t. for 48 h under H_2 gas. The reaction mixture was filtered off through celite and washed two times with EtOAc. The filtrate was washed with water and brine and extracted with EtOAc. The combined organic layer was dried with anhydrous Na_2SO_4. After removal of the solvent the crude product was purified by flash chromatography to obtain 1.9 g of the pure product (**17**) as colorless liquid with 96% yield. ^{1}H NMR (400 MHz, $CDCl_3$) δ 3.76 – 3.61 (m, 2H), 1.67 – 1.45 (m, 4H), 1.34 – 1.11 (m, 7H), 0.99 – 0.78 (m, 9H).

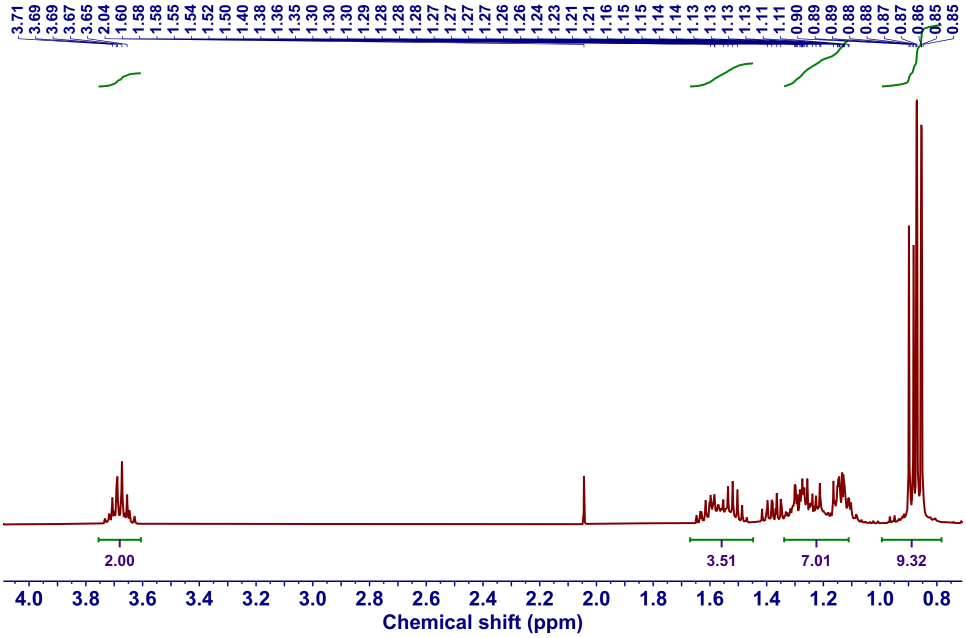

Figure S10a. ^{1}H-NMR spectrum of compound **17**.

S2.3.4. Synthesis of (R)-C3 chiral intermediate[16] step-II:

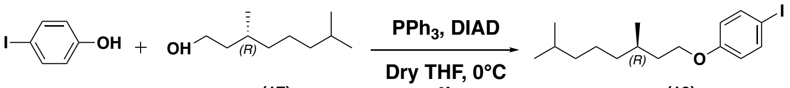

Experimental procedure:

To a stirring solution of compound **17** (1.73 g, 10.91 mmol) and 4-iodophenol, **18** (2.0 g , 9.09 mmol) in dry THF (20.0 mL) was added DIAD (2.76 g, 13.64 mmol) and PPh_3 (3.58 g, 13.64 mmol) at 0 °C. The reaction mixture was stirred at 0 °C for 4 h under an Ar-atmosphere. The reaction was monitored by TLC and quenched with aqueous $NaHCO_3$ solution (100 mL) and extracted two times with EtOAc and the combined organic layer was dried with anhydrous Na_2SO_4. After removal of the solvent the crude product was purified by flash chromatography to obtain 2.3 g of the pure product (**19**) as light-yellow colored liquid with 67% yield. ^{1}H NMR (400 MHz, $CDCl_3$) δ 7.63 – 7.50 (m, 2H, Ar-Ph-H), 6.76 – 6.62 (m, 2H, Ar-Ph-H), 4.04 – 3.90 (m, 2H), 1.83 (dtd, *J* = 13.4, 7.1, 5.1 Hz, 1H), 1.64 – 1.52 (m, 2H), 1.34 (dddd, *J* = 15.0, 7.1, 3.6, 1.9 Hz, 3H), 1.29 – 1.19 (m, 2H), 1.19 – 1.11 (m, 2H), 0.95 (d, *J* = 6.5 Hz, 3H), 0.92 – 0.81 (m, 6H).

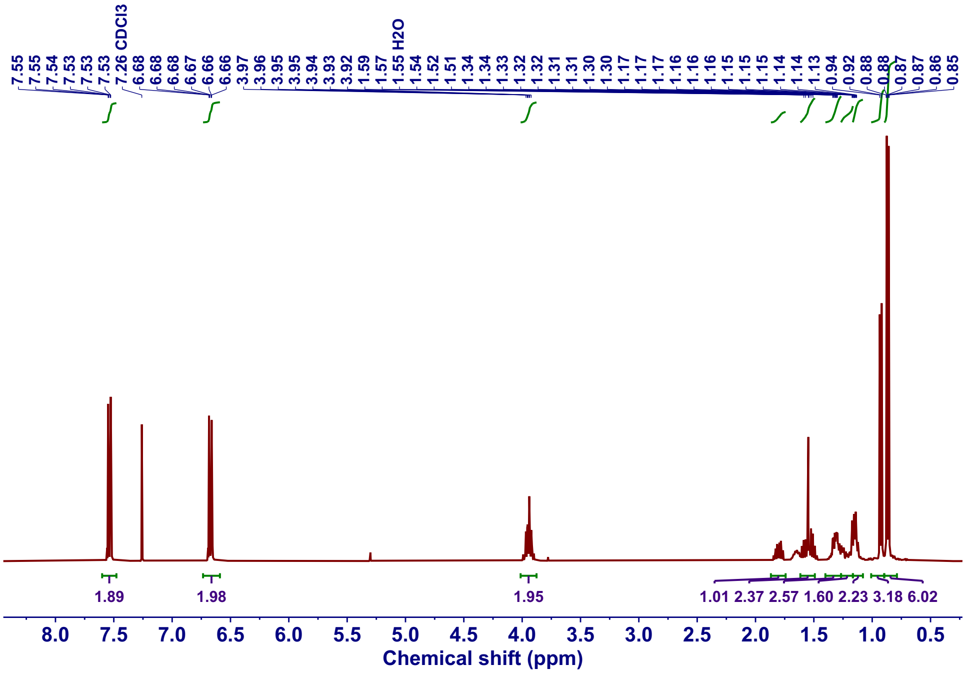

Figure S11a. ^{1}H-NMR spectrum of compound **19**.

S2.3.5. Synthesis of (R)-C3 chiral intermediate step-III:

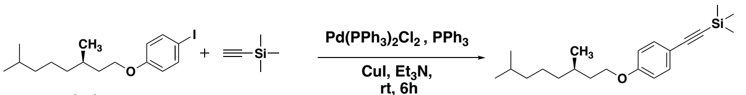

Experimental procedure:

To a stirring solution of compound **19** (2.56 g, 7.11 mmol) in triethylamine (30.0 mL) were added $Pd(PPh_3)_2Cl_2$ (150.2 mg, 0.21 mmol), CuI (80.0 mg, 0.43 mmol), and triphenylphosphine (60.4 mg, 0.43 mmol). The mixture was degassed by bubbling through Ar for 40 min and then trimethylsilylacetylene, **20** (1.32 mL, 9.24 mmol) was added slowly at room temperature. The mixture was stirred at room temperature for 6 h. The reaction mixture was diluted with dichloromethane (50 ml) and filtered through celite on a fritted disk funnel. The filtrate was washed twice with 1M HCl (50 mL), twice with H_2O (200 mL), and then dried over Na_2SO_4. The solvent was removed under vacuum, and the residue was purified by flash chromatography to obtain 2.1 g of pure compound **21** as a colorless liquid with 91% yield. ^{1}H NMR (400 MHz, $CDCl_3$) δ 7.48 – 7.35 (m, 2H), 6.89 – 6.79 (m, 2H), 4.07 – 3.93 (m, 2H), 1.84 (dtd, J = 13.3, 7.1, 5.0 Hz, 1H), 1.75 – 1.53 (m, 3H), 1.40 – 1.27 (m, 3H), 1.24 – 1.14 (m, 3H), 0.96 (d, J = 6.5 Hz, 3H), 0.89 (dd, J = 6.6, 0.5 Hz, 6H), 0.34 – 0.18 (m, 9H).

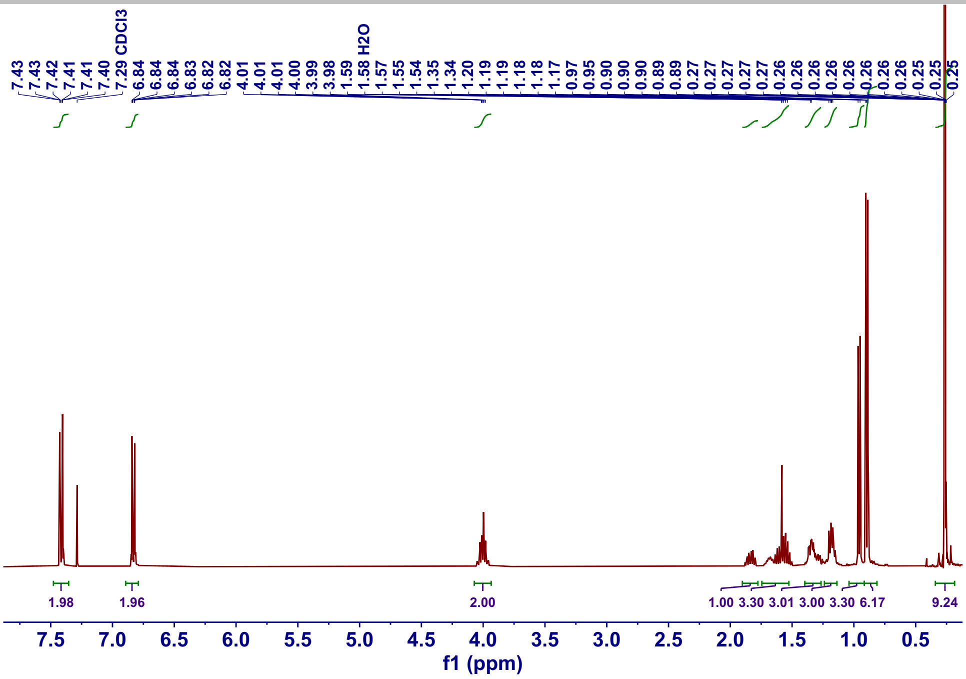

Figure S12a. ^{1}H-NMR spectrum of compound **21**.

S2.3.6. Synthesis of (R)-C3 chiral intermediate step-IV:

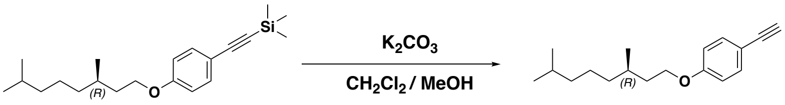

Experimental procedure:
To a stirring solution of compound **21** (2.50 g, 7.56 mmol) in dichloromethane (15.0 mL) was added K_2CO_3 (3.11 g, 22.69 mmol) and methanol (60 mL), the mixture was stirred at room temperature for 1.5 h. The reaction mixture was filtered, and filtrate was washed with water (100 mL) and extracted with dichloromethane (200 mL). The organic layer was washed further with brine (100 mL) and dried over Na_2SO_4. After solvent was removed under vacuum the crude was purified by flash chromatography to obtain 1.9 g pure (*R*)-chiral intermediate (**22**) as a colorless liquid with 98% yield. ^{1}H NMR (400 MHz, $CDCl_3$) δ 7.32 – 7.23 (m, 2H, Ar-Ph-H), 6.73 – 6.65 (m, 2H, Ar-Ph-H), 3.91 – 3.77 (m, 2H), 2.85 (s, 1H), 1.68 (dtd, J = 13.3, 7.0, 5.0 Hz, 1H), 1.59 – 1.31 (m, 3H), 1.27 – 1.11 (m, 3H), 1.11 – 1.03 (m, 1H), 1.03 – 0.95 (m, 2H), 0.80 (d, J = 6.5 Hz, 3H), 0.76 – 0.71 (m, 6H). ^{13}C NMR (101 MHz, $CDCl_3$) δ 19.77, 22.74, 22.85, 24.79, 27.34, 28.10, 29.75, 29.94, 30.10, 36.20, 37.39, 37.56, 39.35, 39.52, 66.48, 75.80, 76.56, 83.90, 113.99, 114.57, 133.67, 159.64. HRMS: m/z calcd. for $C_{18}H_{27}O$, $[M+H]^+$ = 259.2056; observed = 259.2056

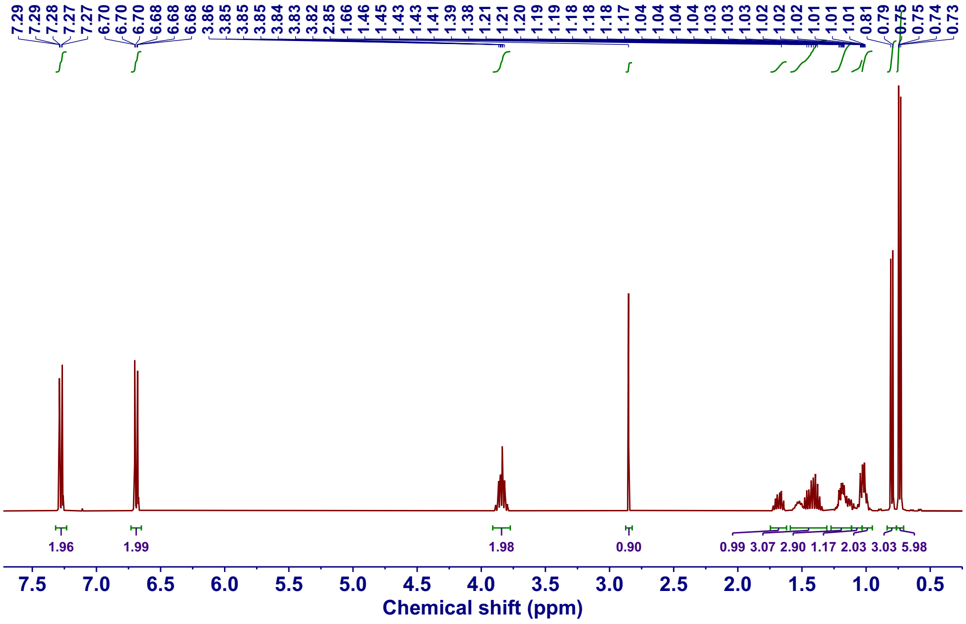

Figure S13a. ^{1}H-NMR spectrum of compound **22**.

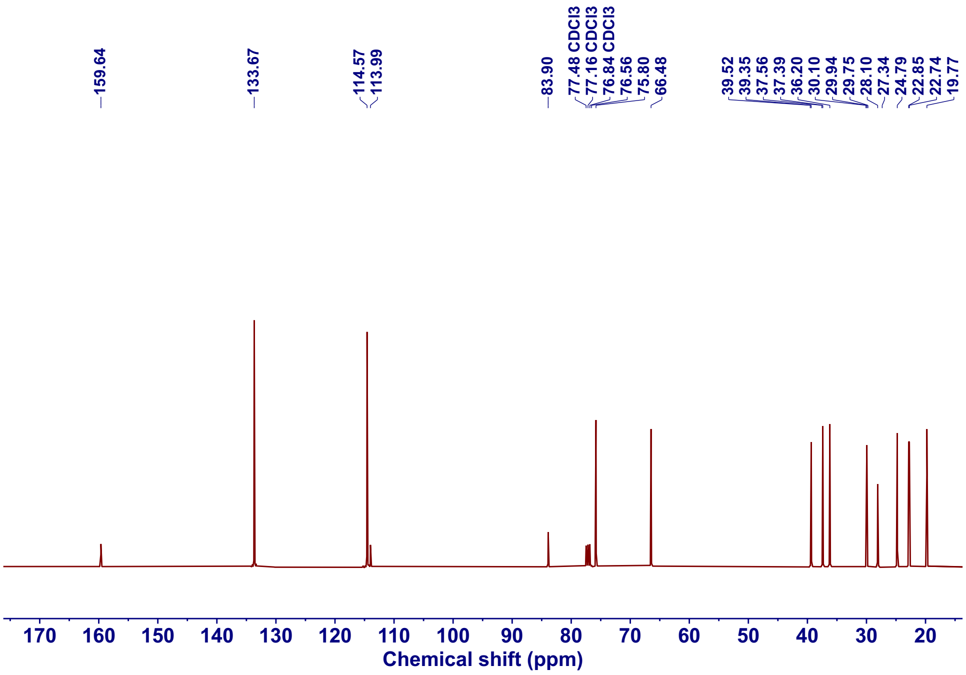

Figure S13b. ^{13}C-NMR spectrum of compound **22**.

*S2.3.7. Synthesis of (R)-C3/T8-SAc thioacetate (**23**):*

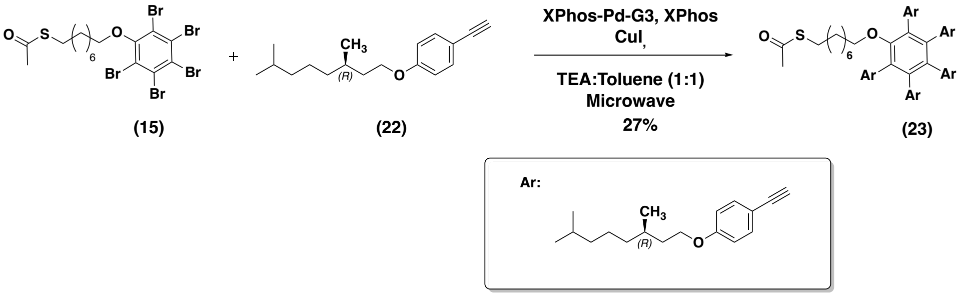

Experimental procedure:

To a mixture of X-PhosPd-G3 (13.2 mg, 0.015 mmol), copper iodide (6.0 mg, 0.029 mmol), XPhos (14.2 mg, 0.029 mmol), compound **15** (0.1 g, 0.148 mmol), and triethylamine (3.0 mL) was added (*R*)-chiral intermediate (**22**) (0.23 g, 0.89 mmol) and toluene (3.0 mL) dropwise under a nitrogen atmosphere. The mixture was degassed for 1 h, then stirred within the microwave synthesizer at 140 °C for 1 h 20 mins. The reaction mixture was diluted with dichloromethane (10 ml) and filtered through celite on a fritted disk funnel. The filtrate was washed twice with 1M HCl (50 mL), twice with H_2O (100 mL), and then dried over Na_2SO_4. After removal of the solvent the crude product was purified by flash chromatography and preparative TLC to obtain 62.1 mg of the pure product (**23**) with 27% yield. ^{1}H NMR (500 MHz, $CDCl_3$) δ 7.57 – 7.48 (m, 10H), 6.92 – 6.84 (m, 10H), 4.34 (t, *J* = 6.3 Hz, 2H), 4.02 (dddd, *J* = 10.4, 9.1, 3.6, 2.2 Hz, 10H), 2.83 (t, *J* = 7.3 Hz, 2H), 2.31 (s, 3H), 1.92 – 1.82 (m, 6H), 1.72 – 1.65 (m, 5H), 1.65 – 1.49 (m, 17H), 1.39 – 1.28 (m, 21H), 1.22 – 1.14 (m, 15H), 0.96 (dd, *J* = 6.7, 1.2 Hz, 15H), 0.88 (d, *J* = 6.6 Hz, 30H). ^{13}C NMR (126 MHz, $CDCl_3$) δ 1.17, 14.27, 19.81, 22.76, 22.86, 24.35, 24.82, 25.94, 26.42, 28.13, 28.95, 29.26, 29.28, 29.58, 29.64, 29.86, 30.01, 30.67, 30.78, 32.08, 36.29, 37.44, 39.39, 66.60, 74.71, 83.68, 86.24, 86.77, 97.16, 99.26, 99.41, 114.75, 114.77, 115.44, 115.48, 115.69, 120.00, 124.02, 128.55, 133.24, 133.28, 133.43, 159.56, 159.68, 159.76, 159.97, 196.13. HRMS: m/z calcd. for $C_{106}H_{146}O_7S$, $[M\text{-}2H+H]^+$ = 1563.0739; observed = 1563.0745.

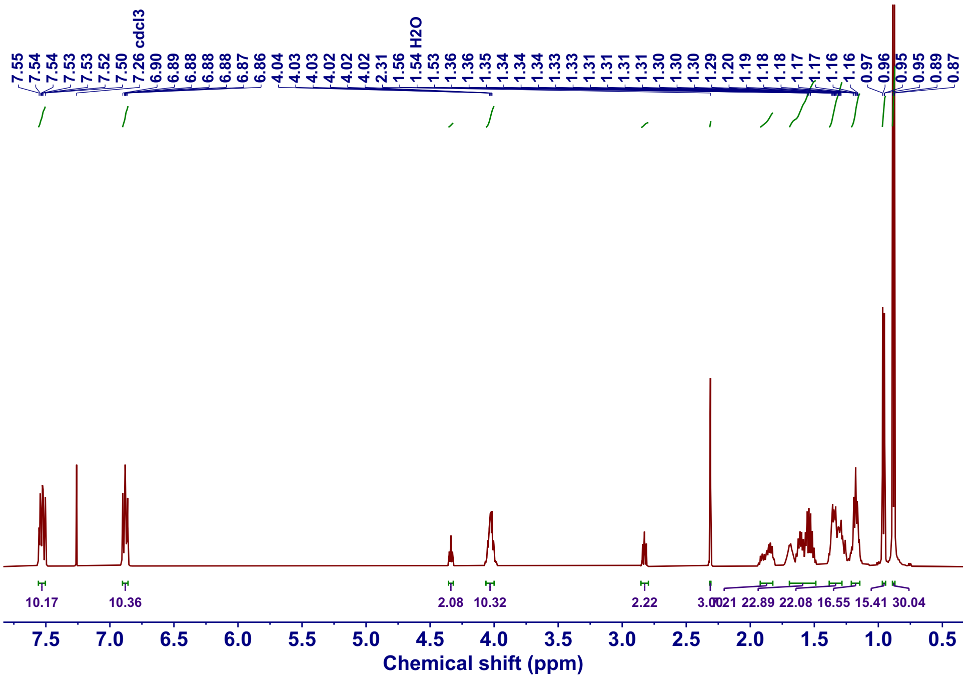

Figure S14a. ^{1}H-NMR spectrum of compound **23**.

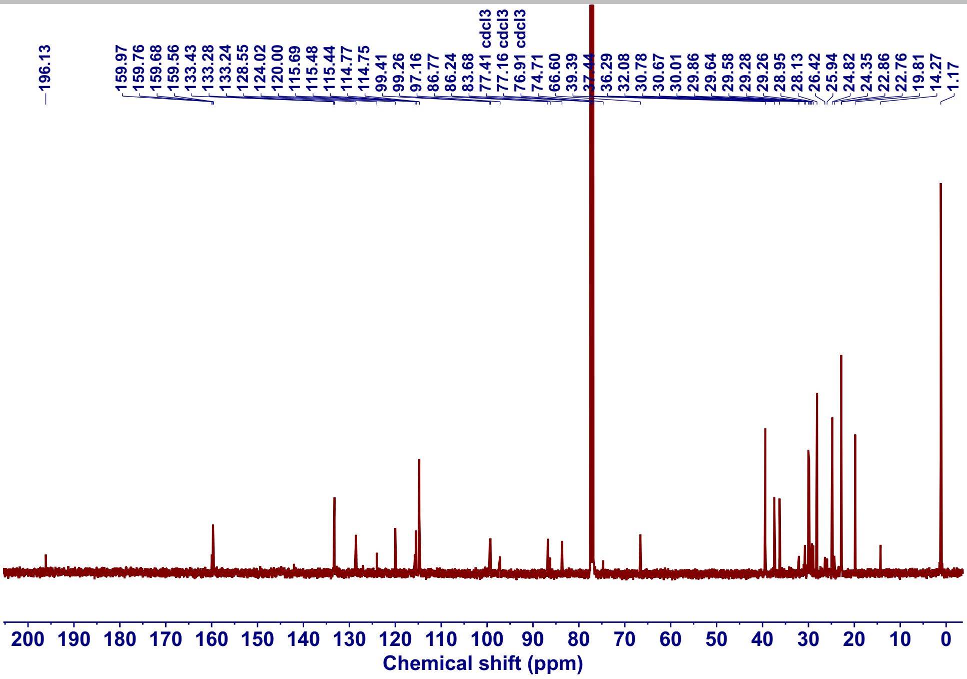

Figure S14b. ^{13}C-NMR spectrum of compound **23**.

S2.4. Synthesis of (*S*)-C1/T8-SAc chiral solute

S2.4.1. Synthesis of (S)-C1 chiral intermediate step-I:

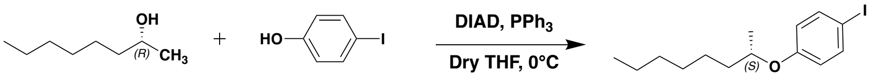

Experimental procedure:

To a stirring solution of compound **24** (1.42 g, 10.91 mmol) and 4-iodophenol, **18** (2.0 g, 9.09 mmol) in dry THF (20.0 mL) was added DIAD (2.76 g, 13.64 mmol) and PPh_3 (3.58 g, 13.64 mmol) at 0 °C. The reaction mixture was stirred at 0 °C for 4 h under an Ar-atmosphere. The reaction was monitored by TLC and quenched with aqueous $NaHCO_3$ solution (100 mL) and extracted two times with EtOAc and the combined organic layer was dried with anhydrous Na_2SO_4. After removal of the solvent the crude product was purified by flash chromatography to obtain 2.06 g of the pure product (26) as colorless liquid with 68% yield. ^1H NMR (400 MHz, $CDCl_3$) δ 7.58 – 7.48 (m, 2H, Ar-Ph-H), 6.71 – 6.61 (m, 2H, Ar-Ph-H), 4.30 (h, *J* = 6.1 Hz, 1H), 1.75 – 1.66 (m, 1H), 1.60 – 1.50 (m, 1H), 1.44 – 1.29 (m, 5H), 1.29 – 1.21 (m, 6H), 0.95 – 0.84 (m, 3H). ^{13}C NMR (101 MHz, $CDCl_3$) δ 14.22, 19.73, 22.73, 25.59, 29.38, 31.74, 31.91, 36.47, 74.23, 82.37, 117.69, 117.99, 118.35, 119.25, 138.04, 138.34, 138.63, 158.24.

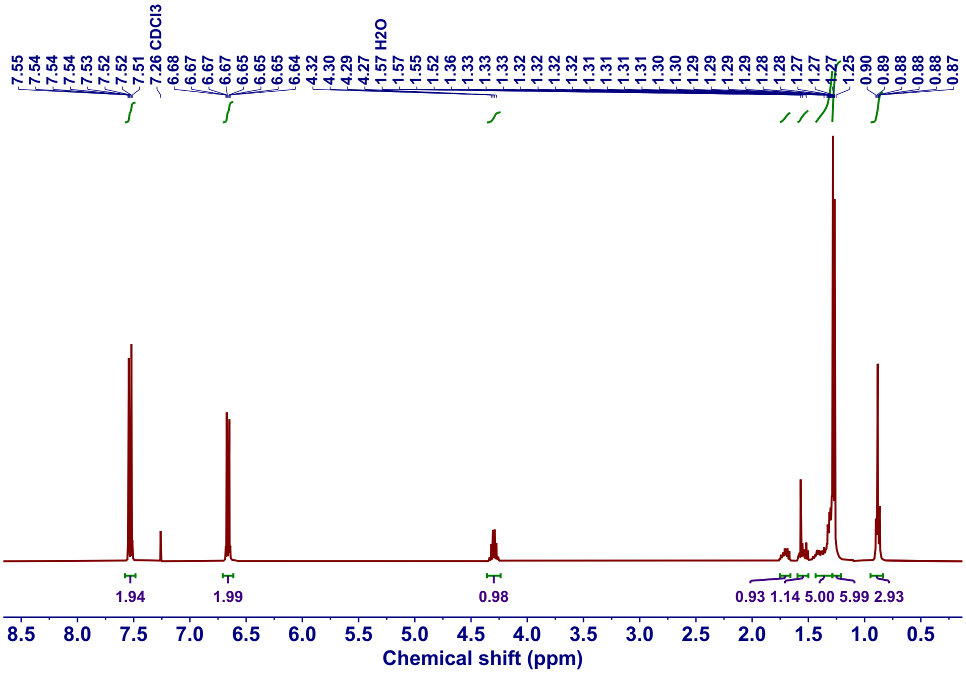

Figure S15a. ^{1}H-NMR spectrum of compound **26**.

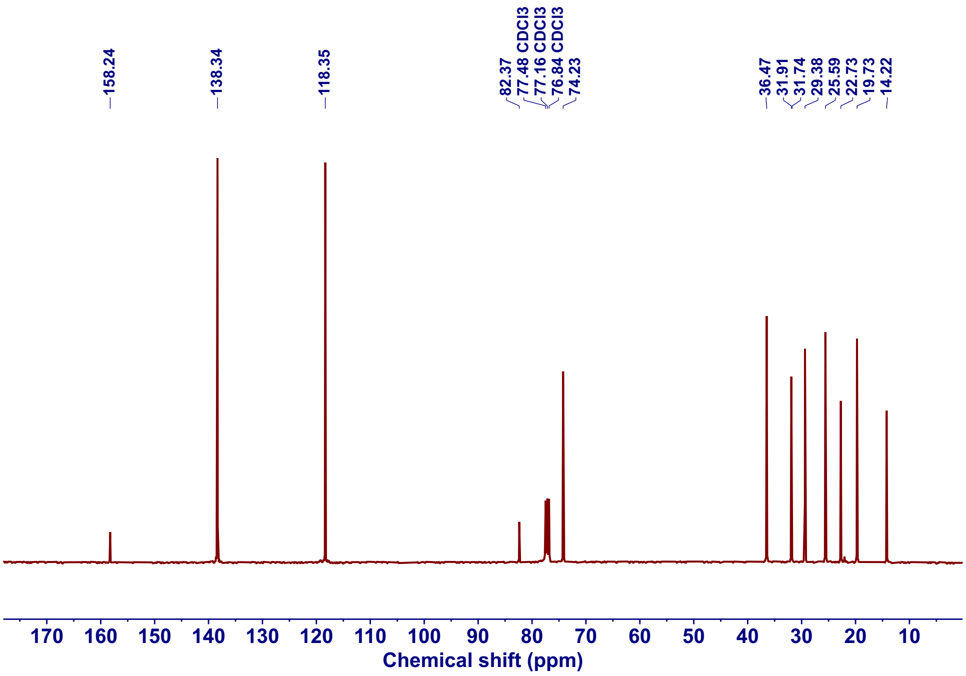

Figure S15b. ^{13}C-NMR spectrum of compound **26**.

S2.4.2. Synthesis of (S)-C1 chiral intermediate[14] *step-II*

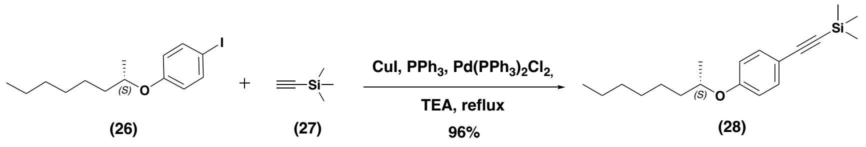

(26) **(27)** **(28)**

Experimental procedure:

To a stirring solution of compound **26** (2.15 g, 6.47 mmol) in triethylamine (30 mL) were added $Pd(PPh_3)_2Cl_2$ (150.2 mg, 0.21 mmol), CuI (80.0 mg, 0.43 mmol), and triphenylphosphine (60.4 mg, 0.43 mmol). The mixture was degassed by bubbling through Ar for 40 min and then trimethylsilylacetylene, **27** (1.32 mL, 9.24 mmol) was added slowly at room temperature. The mixture was stirred at room temperature for 6 h. The reaction mixture was diluted with dichloromethane (50 ml) and filtered through celite on a fritted disk funnel. The filtrate was washed twice with 1M HCl (50 mL), twice with H_2O (200 mL), and dried over Na_2SO_4. The solvent was removed under vacuum, and the residue was purified by flash chromatography to obtain 1.85 g of pure compound **28** as a colorless liquid with 91% yield. ^1H NMR (400 MHz, $CDCl_3$) δ 7.42 – 7.34 (m, 2H, Ar-Ph-H), 6.82 – 6.74 (m, 2H, Ar-Ph-H), 4.35 (h, J = 6.1 Hz, 1H), 1.71 (dddd, J = 13.5, 9.9, 6.4, 5.1 Hz, 1H), 1.61 – 1.48 (m, 2H), 1.37 – 1.29 (m, 3H), 1.29 – 1.25 (m, 7H), 0.92 – 0.83 (m, 3H), 0.23 (s, 9H). ^{13}C NMR (101 MHz, $CDCl_3$) δ 14.23, 19.78, 22.74, 25.58, 29.39, 31.92, 36.50, 74.05, 92.35, 105.48, 114.92, 115.68, 133.61, 158.61

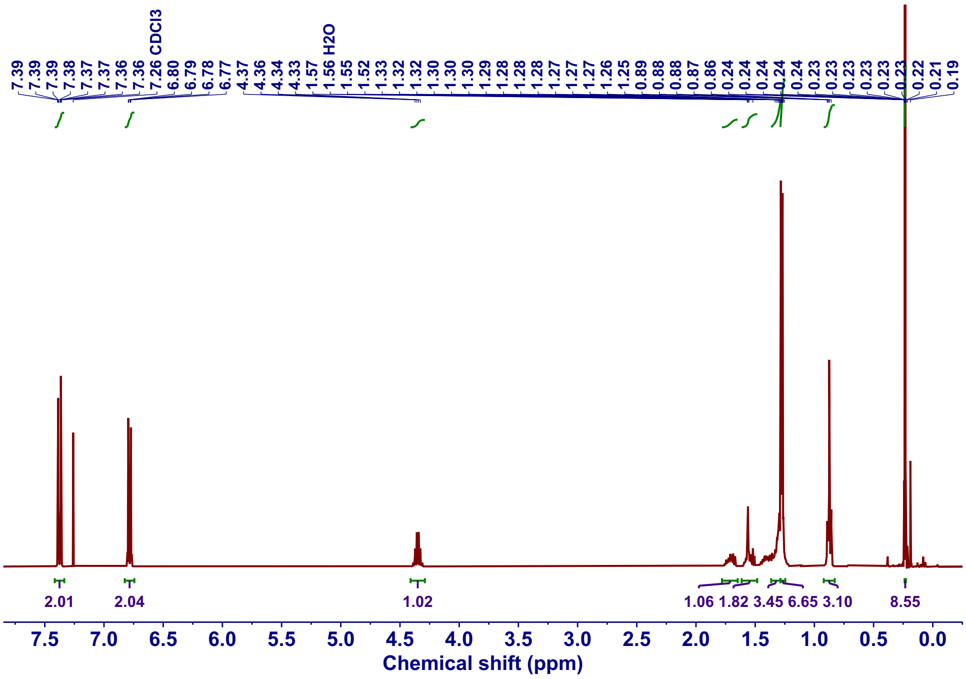

Figure S16a. ^1H-NMR spectrum of compound **28**.

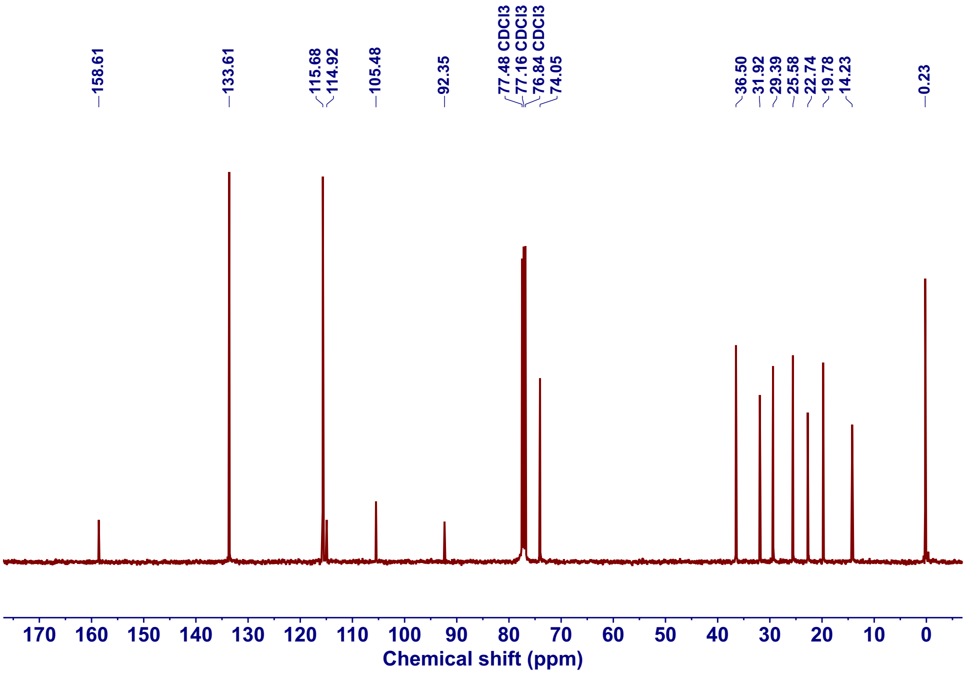

Figure S16b. ^{13}C-NMR spectrum of compound **28**.

S2.4.3. Synthesis of (S)-C1 chiral intermediate step-III

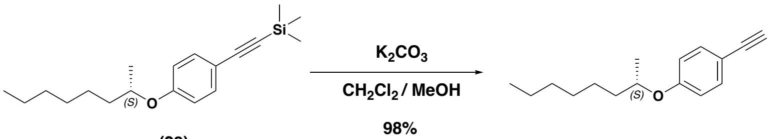

(28) (29)

Experimental procedure:

To a stirring solution of compound **28** (2.0 g, 6.61 mmol) in dichloromethane (15.0 mL) was added K_2CO_3 (9.14 g, 66.11mmol) and methanol (60.0 mL), the mixture was stirred at room temperature for 1.5 h. The reaction mixture was filtered, and the filtrate was washed with water (100 mL) and extracted with dichloromethane (200 mL). The organic layer was washed further with brine (100 mL) and dried over Na_2SO_4. After solvent was removed under vacuum the crude was purified by flash chromatography to obtain 1.5 g pure (*S*)-chiral intermediate (**29**) as a colorless liquid with 98% yield. ^{1}H NMR (400 MHz, $CDCl_3$) δ 7.45 – 7.37 (m, 2H, Ar-Ph-H), 6.85 – 6.77 (m, 2H, Ar-Ph-H), 4.36 (h, *J* = 6.1 Hz, 1H), 2.99 (s, 1H), 1.73 (dddd, *J* = 13.4, 9.9, 6.5, 5.0 Hz, 1H), 1.63 – 1.49 (m, 1H), 1.49 – 1.33 (m, 2H), 1.33 – 1.21 (m, 9H), 0.93 – 0.84 (m, 3H). ^{13}C NMR (101 MHz, $CDCl_3$) δ 1.05, 14.10, 19.66, 22.62, 25.48, 29.26, 31.80, 36.38, 73.93, 75.59, 76.35, 83.82, 113.67, 115.60, 133.62, 158.69. GC-MS: m/z calcd. for $C_{16}H_{22}O$, $[M]^+$, $[M+H]^+$ = 230.1671, 231.1704; observed = 230.1663, 231.1699.

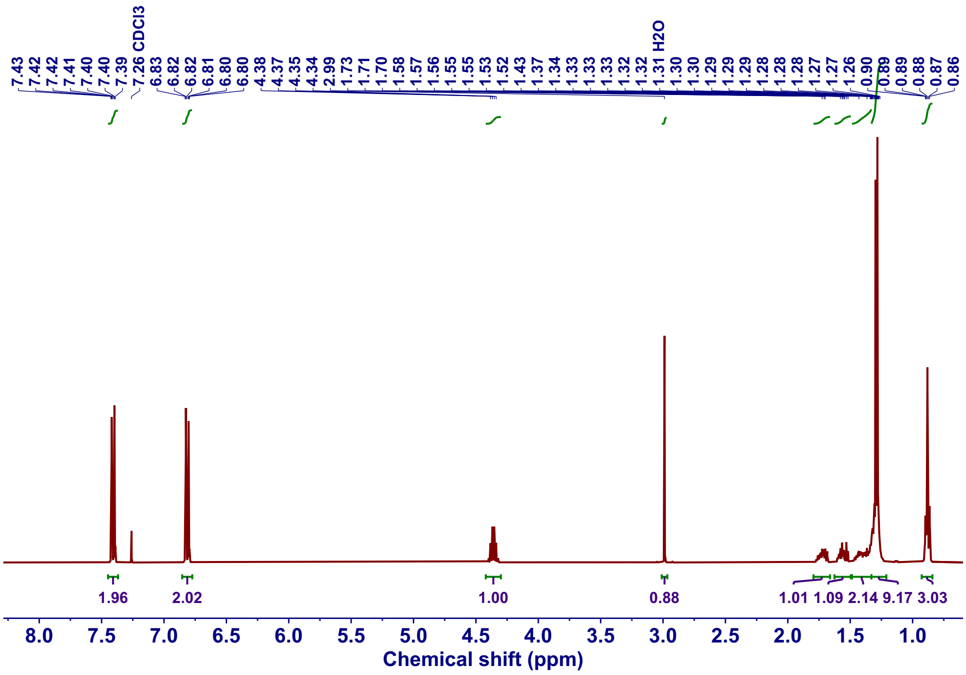

Figure S17a. ^{1}H-NMR spectrum of compound **29**.

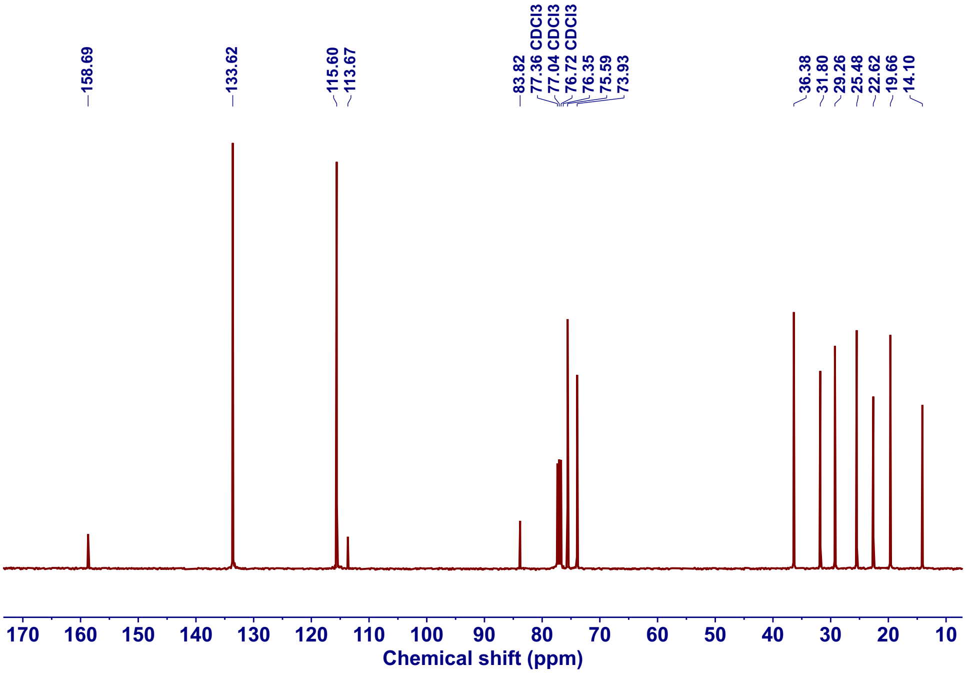

Figure S17b. ^{13}C-NMR spectrum of compound **29**.

S2.4.4. Synthesis of (S)-C1/T8-SAc thioacetate

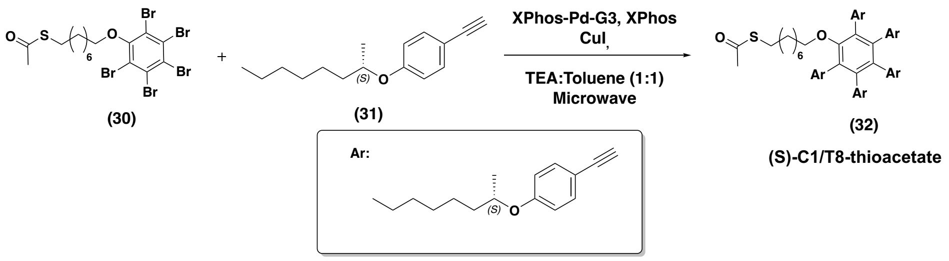

Experimental procedure:
To a mixture of X-PhosPd-G3 (13.2 mg, 0.015 mmol), copper iodide (6.0 mg, 0.029 mmol), XPhos (14.2 mg, 0.029 mmol), compound **30** (0.1 g, 0.148 mmol), and triethylamine (3.0 mL) was added (*S*)-chiral intermediate (**31**) (0.18 g, 0.77 mmol) and toluene (3.0 mL) dropwise under a nitrogen atmosphere. The mixture was degassed for 1 h and then stirred within the microwave synthesizer at 140 °C for 1h 20 mins. The reaction mixture was diluted with dichloromethane (10 ml) and filtered through celite on a fritted disk funnel. The filtrate was washed twice with 1M HCl (50 mL), twice with H_2O (100 mL), and dried over Na_2SO_4. After removal of the solvent the crude product was purified by flash chromatography and preparative TLC to obtain 70 mg (yield = 33%) of the pure product (**32**). ^{1}H NMR (400 MHz, $CDCl_3$) δ 7.59 – 7.47 (m, 10H), 6.91 – 6.81 (m, 10H), 4.40 (ddt, *J* = 9.0, 6.1, 2.9 Hz, 5H), 4.33 (t, *J* = 6.3 Hz, 2H), 2.88 – 2.79 (m, 2H), 2.31 (s, 3H), 1.91 – 1.82 (m, 2H), 1.75 (dddd, *J* = 13.3, 10.0, 6.3, 1.5 Hz, 6H), 1.64 – 1.39 (m, 21H), 1.34 – 1.28 (m, 48H), 0.90 – 0.86 (m, 15H). ^{13}C NMR (101 MHz, $CDCl_3$) δ 1.17, 14.24, 19.84, 22.75, 22.96, 24.23, 25.64, 26.02, 26.10, 26.42, 26.86, 28.94, 29.25, 29.28, 29.42, 29.58, 29.62, 29.85, 30.68, 30.78, 30.94, 31.94, 36.58, 74.11, 74.68, 83.61, 86.19, 86.72, 97.19, 99.27, 99.44, 115.26, 115.30, 115.53, 115.88, 119.99, 120.39, 124.03, 128.53, 133.29, 133.34, 133.49, 158.68, 158.80, 158.88, 159.92, 196.17. HRMS: m/z calcd. for $C_{95}{}^{13}CH_{125}O_7{}^{32}S$, $[M+H]^+$ = 1422.9141; observed = 1422.9135.

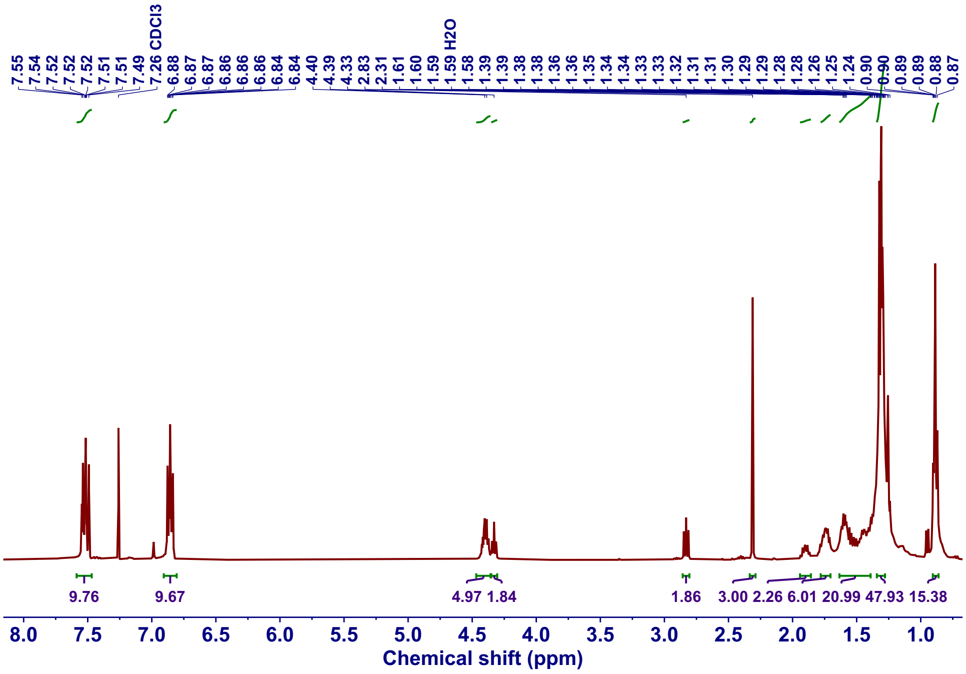

Figure S18a. ^{1}H-NMR spectrum of compound **32**.

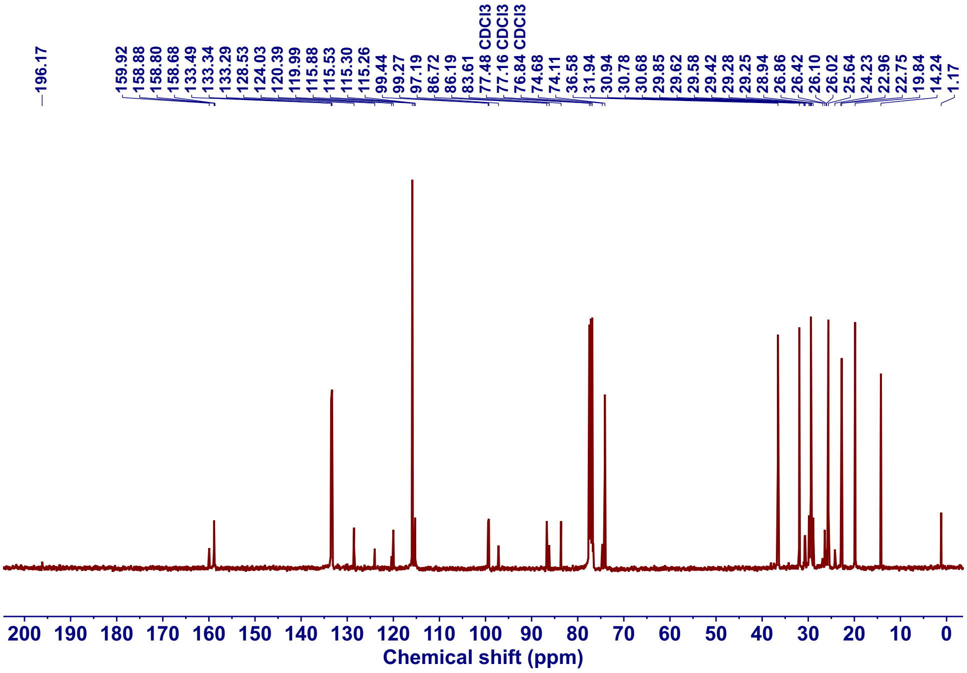

Figure S18b. ^{13}C-NMR spectrum of compound **32**.

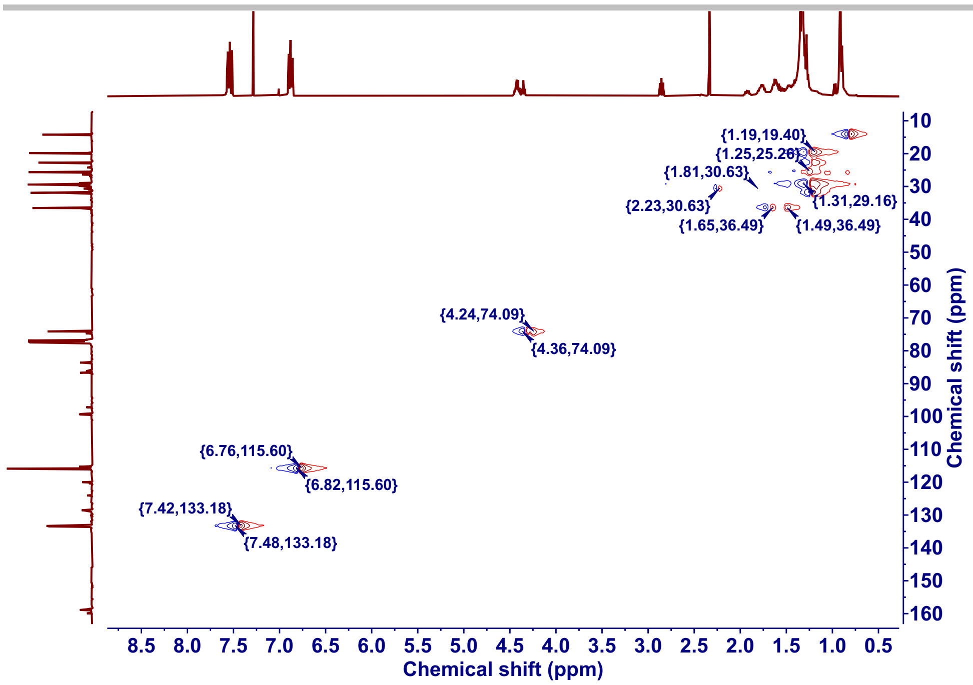

Figure S18c. {^{1}H -^{13}C} -HSQC NMR spectrum of compound **32**.

S2.5. Synthesis of (*S*)-C1/T12-SAc chiral solute and (*S*)-C1/T12-SH ligand

*S2.5.1. Synthesis of pentabromo-C12 intermediate (**35**):*

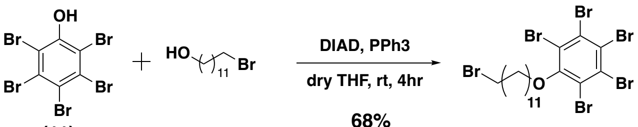

Experimental procedure:

To a stirring solution of pentabromophenol, **11** (3.00 g, 6.14 mmol) and compound **34** (1.95 g , 7.37 mmol) in dry THF (20.0 mL) was added DIAD (1.61 g, 7.98 mmol) and PPh_3 (2.09 g, 7.98 mmol) at 0 °C. The reaction mixture was stirred at 0 °C for 4 h under an Ar-atmosphere. The reaction was monitored by TLC and quenched with aqueous $NaHCO_3$ solution (100 mL) and extracted two times with EtOAc and the combined organic layer was dried with anhydrous Na_2SO_4. After removal of the solvent the crude product was purified by flash chromatography to obtain 5.17 g of the pure product (**35**) as white solid with 68% yield. ^{1}H NMR (400 MHz, $CDCl_3$) δ 3.99 (t, J = 6.6 Hz, 2H), 3.41 (t, J = 6.9 Hz, 2H), 1.93 – 1.80 (m, 4H), 1.50 (dt, J = 7.4, 5.5 Hz, 2H), 1.46 – 1.31 (m, 6H), 1.31 – 1.26 (m, 8H). ^{13}C NMR (101 MHz, $CDCl_3$) δ 25.93, 28.32, 28.91, 29.54, 29.57, 29.65, 29.67, 29.68, 30.00, 32.98, 34.26, 73.80, 122.05, 124.60, 128.48, 154.71.

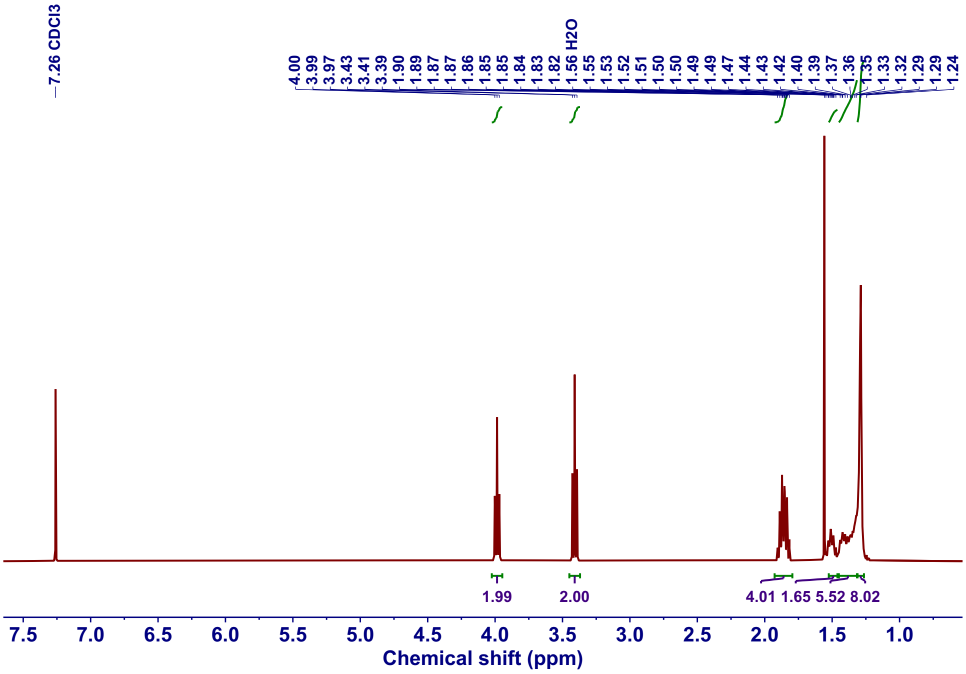

Figure S19a. ^{1}H-NMR spectrum of compound **35**.

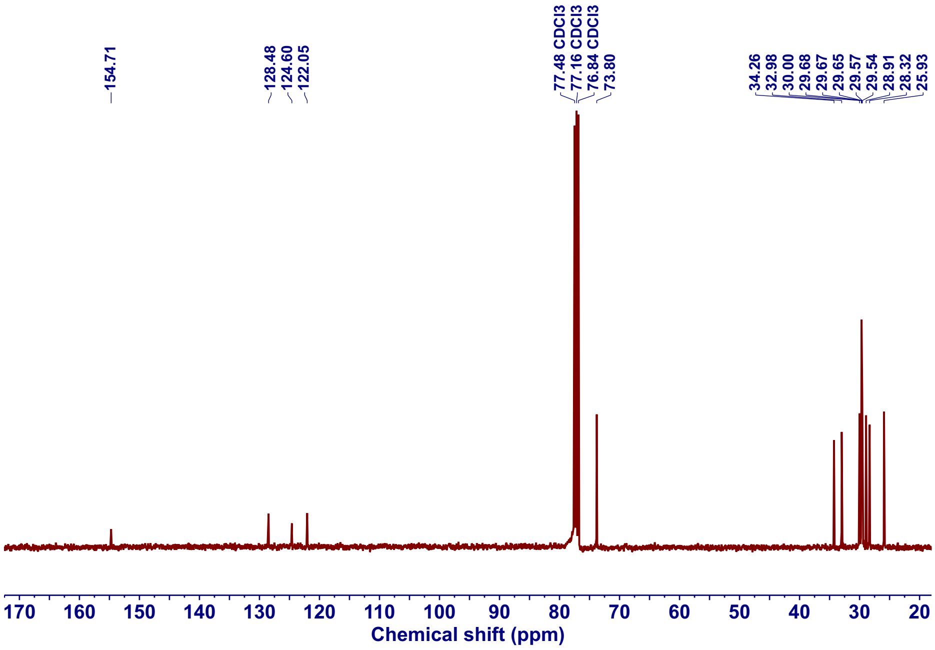

Figure S19b. ^{13}C-NMR spectrum of compound **35**.

*S2.5.2. Synthesis of pentabromo-C12 thioacetate (**36**):*

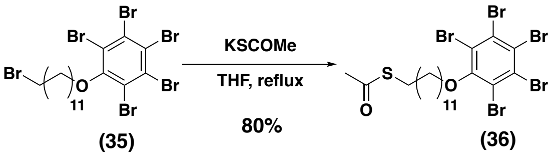

Experimental procedure:

To a stirring solution of compound **35** (3.0 g, 5.05 mmol) in dry THF (50.0 mL) was added potassium thioacetate (0.86 g, 7.57 mmol). The reaction mixture was refluxed for 16 h. The reaction was monitored by TLC and quenched with aqueous sodium bisulfite solution (100 mL). The reaction mixture was washed additionally with water (100 mL), brine (100 mL) and extracted with EtOAc, and the combined organic layer was dried with anhydrous Na_2SO_4. After removal of the solvent the crude product was purified by flash chromatography to obtain 2.4 g of the pure product (**36**) as white solid with 80% yield. ^{1}H NMR (400 MHz, $CDCl_3$) δ 3.98 (t, J = 6.6 Hz, 2H), 2.86 (t, J = 7.4 Hz, 2H), 2.32 (s, 3H), 1.87 (dt, J = 14.7, 6.7 Hz, 2H), 1.54 (tdd, J = 15.7, 8.2, 5.8 Hz, 5H), 1.37 – 1.24 (m, 13H). ^{13}C NMR (101 MHz, $CDCl_3$) δ 25.92, 28.97, 29.26, 29.30, 29.54, 29.60, 29.64, 29.67, 29.69, 30.00, 30.82, 73.80, 122.05, 124.58, 128.47, 154.71, 196.28. HRMS: m/z calcd. for $C_{20}H_{28}{}^{79}Br_3{}^{81}Br_2O_2{}^{32}S$, $C_{20}H_{28}{}^{79}Br_2{}^{81}Br_3O_2{}^{32}S$, $[M+H]^+$ = 730.76805, 732.76600; observed = 730.7669, 732.7647.

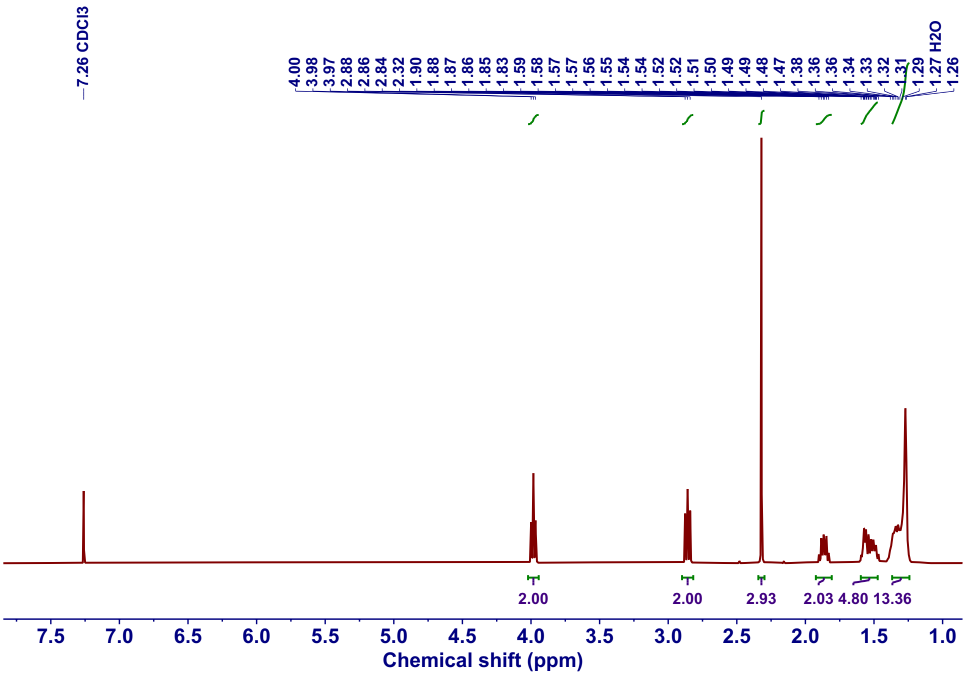

Figure S20a. ^{1}H-NMR spectrum of compound **36**.

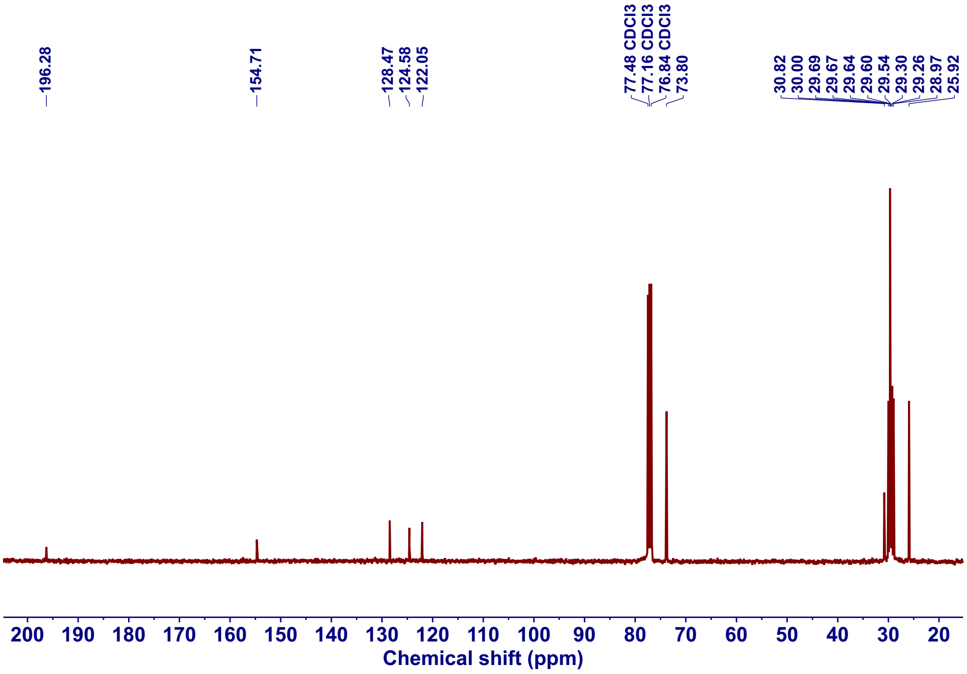

Figure S20b. ^{13}C-NMR spectrum of compound **36**.

*S2.5.3. Synthesis of (S)-C1/T12-SAc thioacetate (**37**):*

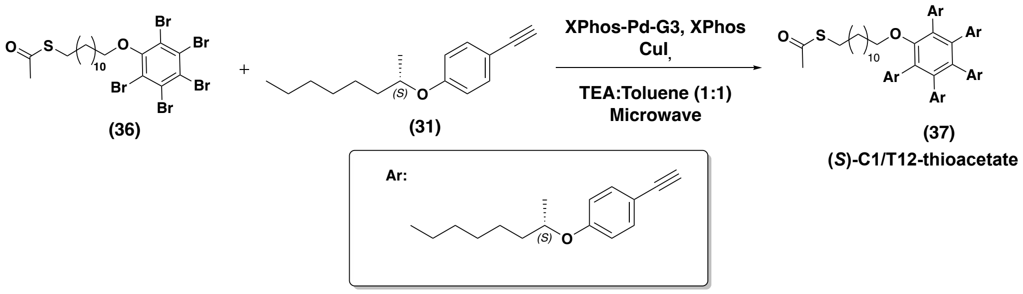

Experimental procedure:

To a mixture of X-Phos-Pd-G3 (116.0 mg, 0.137 mmol), Xphos (0.065 g, 0.1368 mmol), copper iodide (0.052 mg, 0.2736 mmol), compound **36** (1.0g, 1.368 mmol), and triethylamine (3.0 mL) was added compound **31** (1.64g, 7.11 mmol) in toluene (3.0 mL) dropwise under a nitrogen atmosphere. The mixture was stirred at microwave at 140 °C for 1 h 40 min and then cooled. To the cooled reaction mixture was added dichloromethane (100 mL). The mixture was washed twice with aqueous NH_4Cl (200 mL), twice with H_2O (200 mL), and dried over $MgSO_4$. After removal of the solvents, the residue was purified by column chromatography (*n*-hexane/dichloromethane 5:1) to yield 300 mg of **37** (yield = 15%) as an orange liquid. ^{1}H NMR (400 MHz, $CDCl_3$) δ 7.56 – 7.49 (m, 10H, Ar-Ph-H), 6.88 – 6.82 (m, 10H, Ar-Ph-H), 4.40 (p, J = 6.1 Hz, 5H), 4.33 (t, J = 6.4 Hz, 2H), 2.88 – 2.83 (m, 2H), 2.31 (s, 3H), 1.94 – 1.87 (m, 2H), 1.80 – 1.70 (m, 5H), 1.61 – 1.52 (m, 11H), 1.35 – 1.23 (m, 64H), 0.93 – 0.81 (m, 18H). ^{13}C NMR (101 MHz, $CDCl_3$) δ 1.17, 14.24, 19.84, 22.75, 25.64, 26.52, 28.99, 29.30, 29.42, 29.65, 29.74, 29.78, 30.74, 30.79, 31.94, 36.57, 74.11, 74.77, 76.57, 77.36, 83.62, 86.19, 86.72, 97.16, 99.26, 99.41, 115.27, 115.32, 115.53, 115.87, 120.01, 124.01, 128.50, 133.30, 133.34, 133.48, 158.67, 158.78, 158.88, 159.96, 196.23. HRMS: m/z calcd. for $C_{100}H_{132}O_7{}^{32}S^{23}Na$, $C_{99}{}^{13}CH_{132}O_7{}^{32}S^{23}Na$, $[M+Na]^+$ = 1499.95860, 1500.96195; observed = 1499.9545, 1500.9574.

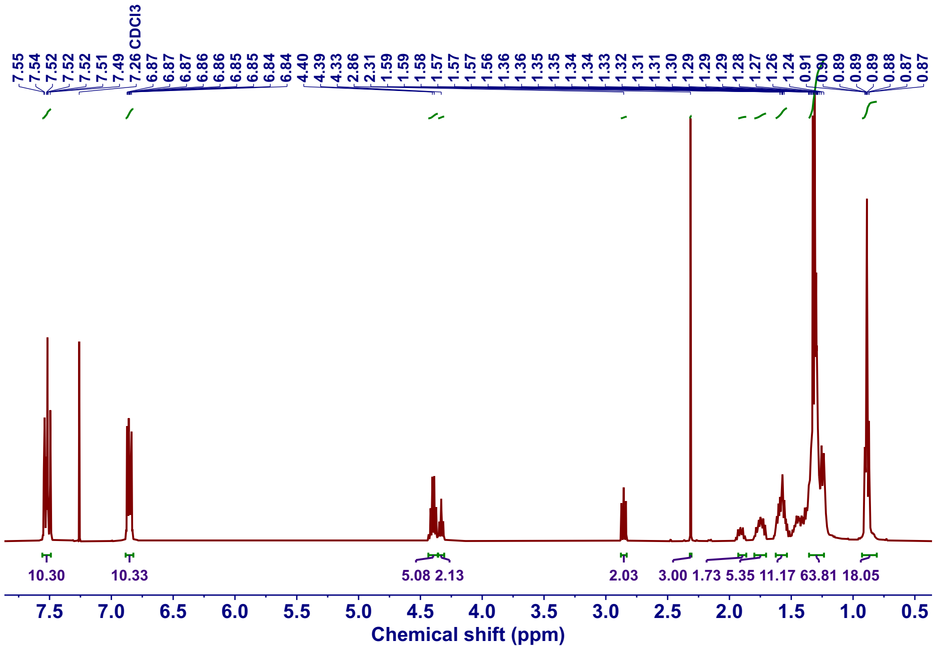

Figure S21a. ^{1}H-NMR spectrum of compound **37**.

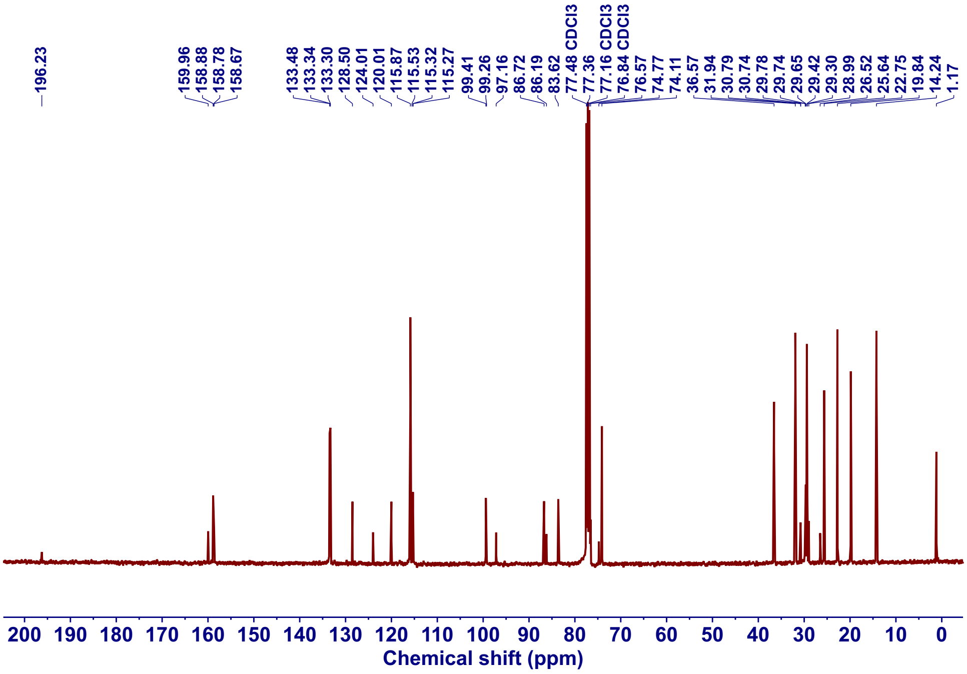

Figure S21b. ^{13}C-NMR spectrum of compound **37**.

*S2.5.3. Synthesis of (S)-C1/T12-SH thiol (**38**):*

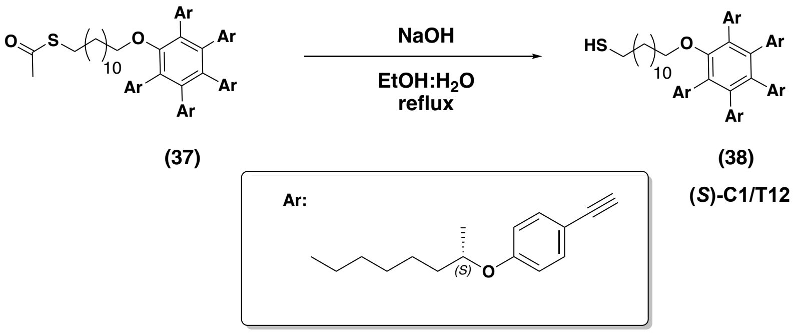

Experimental procedure:

To a mixture of compound **32** (0.25 g, 0.169 mmol) in ethanol (10.0 mL) was added aqueous NaOH (7.0M, 5.0 mL) dropwise. The mixture was refluxed for 4 h and then cooled. To the cooled reaction mixture was added dichloromethane (50 mL). The mixture was washed twice with H_2O (50 mL) and dried over $MgSO_4$. After removal of the solvents the residue was purified by column chromatography using neutral alumina as a stationary phase, followed by preparative TLC (*n*-hexane/dichloromethane 5:1) to yield 90 mg of **38** (yield = 37%) as an orange liquid. ^1H NMR (400 MHz, $CDCl_3$) δ 7.57 – 7.46 (m, 10H, Ar-Ph-H), 6.90 – 6.80 (m, 10H, Ar-Ph-H), 4.40 (p, J = 6.1 Hz, 5H), 4.32 (t, J = 6.3 Hz, 2H), 2.69 – 2.64 (m, 2H), 1.93 – 1.88 (m, 2H), 1.76 – 1.73 (m, 2H), 1.64 – 1.58 (m, 5H), 1.45 – 1.36 (m, 10H), 1.36 – 1.24 (m, 64H), 0.92 – 0.84 (m, 18H). ^{13}C NMR (101 MHz, $CDCl_3$) δ 1.17, 14.25, 19.84, 22.76, 25.64, 26.55, 28.75, 29.42, 29.47, 29.74, 29.82, 29.85, 30.76, 31.94, 36.57, 39.25, 74.10, 74.77, 83.62, 86.20, 86.72, 97.16, 99.26, 99.41, 115.27, 115.31, 115.53, 115.87, 120.01, 124.02, 128.50, 133.30, 133.34, 133.48, 158.66, 158.78, 158.87, 159.96.

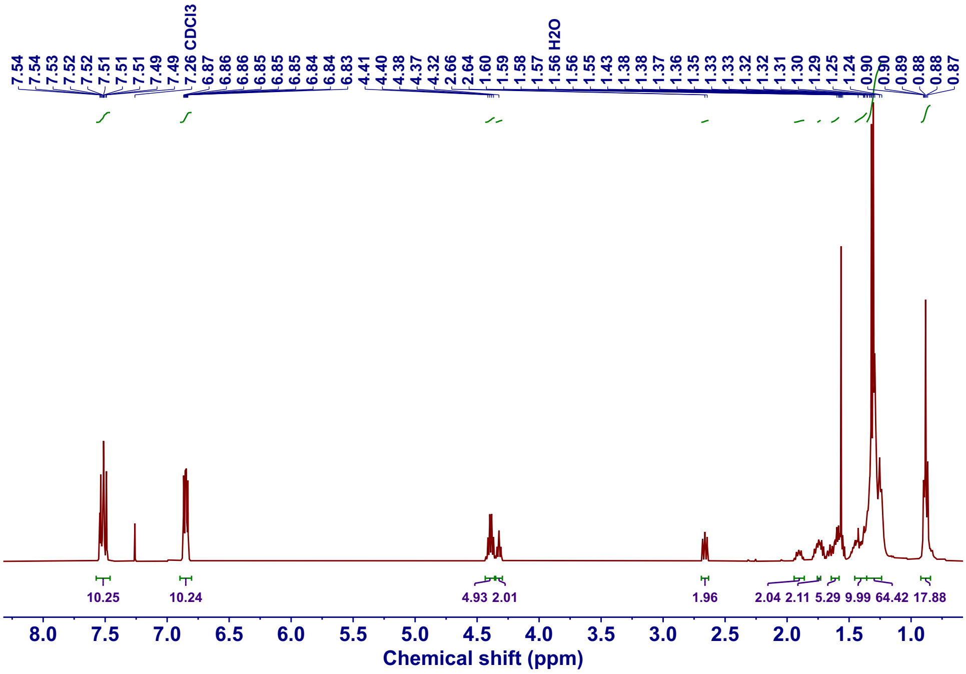

Figure S22a. ^1H-NMR spectrum of compound **38**.

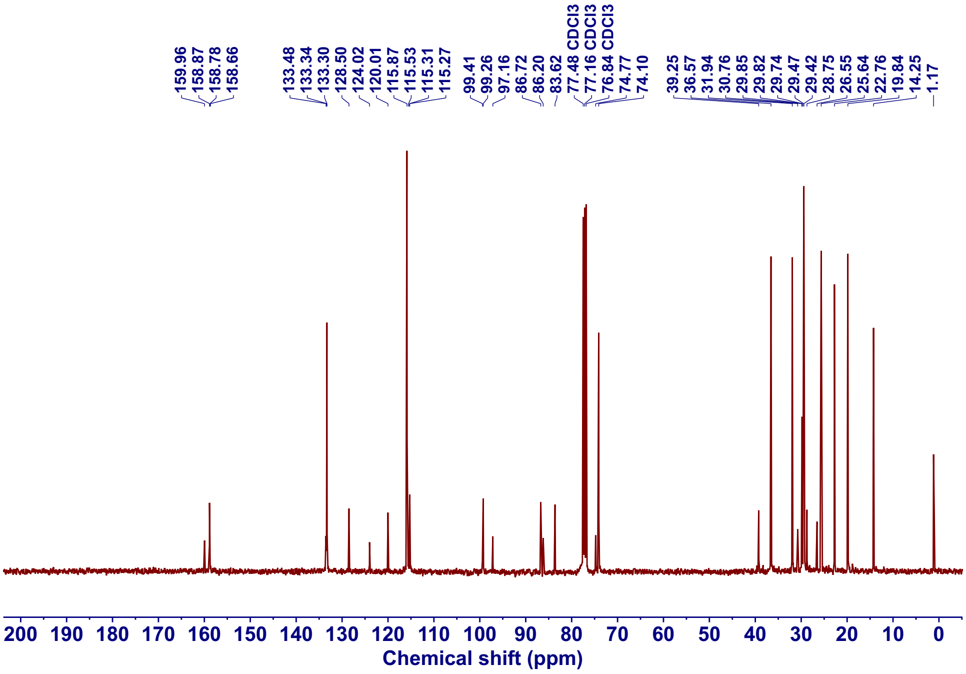

Figure S22b. ^{13}C-NMR spectrum of compound **38**.

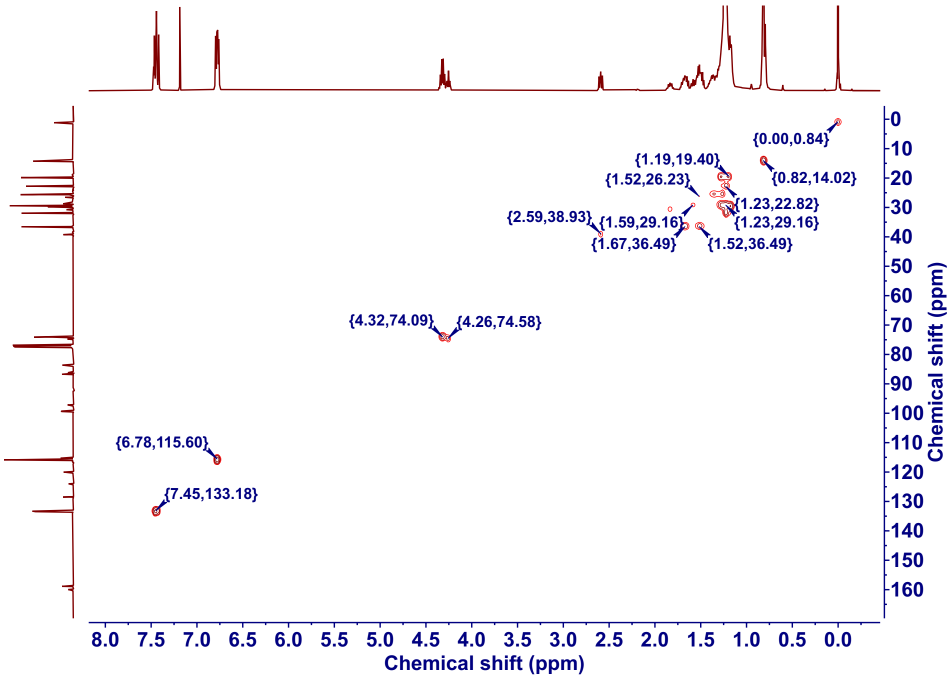

Figure S22c. {^{1}H -^{13}C}-HSQC spectrum of compound **38**.

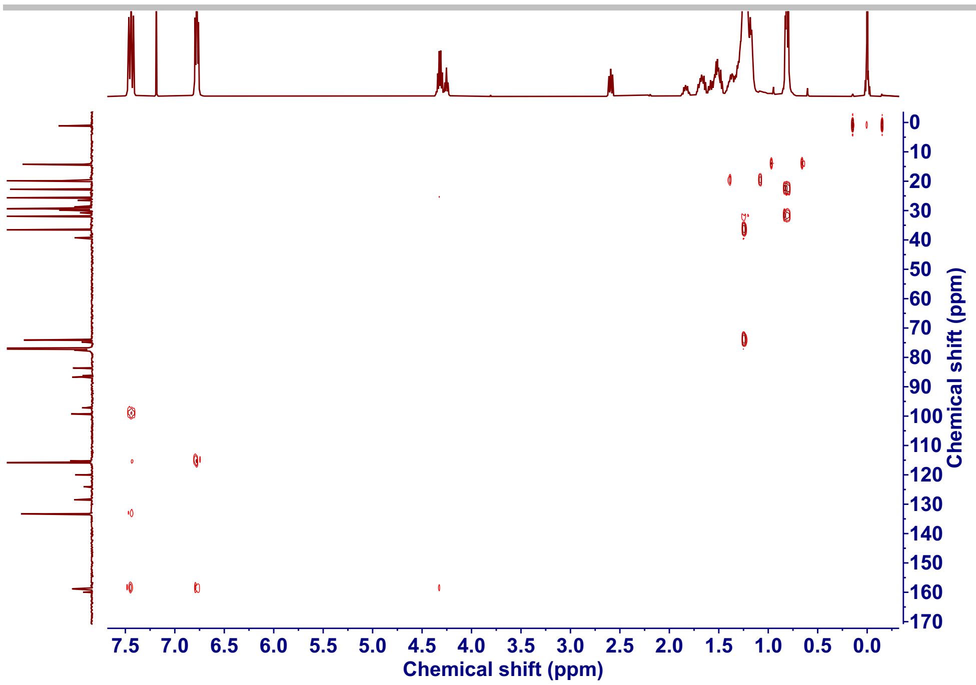

Figure S22d. {^{1}H -^{13}C}-HMBC spectrum of compound **38**.

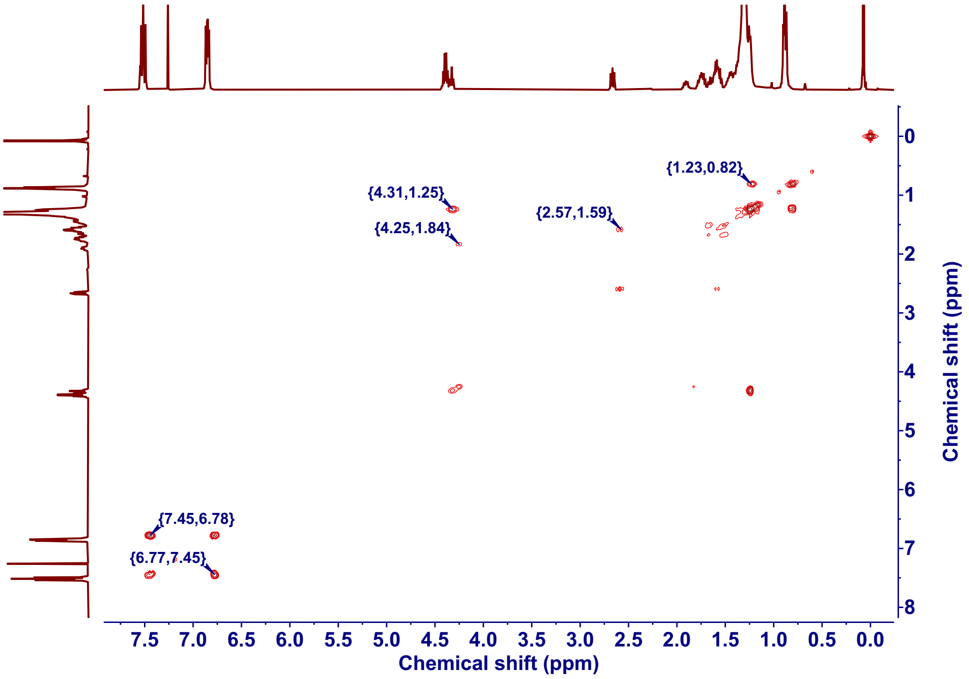

Figure S22e. {^{1}H -^{1}H}-COSY spectrum of compound **38**.

S2.6. Synthesis of (*R*)-C1/T12-SAc chiral solute

S2.6.1. Synthesis of (R)-C1 chiral intermediate step-I:

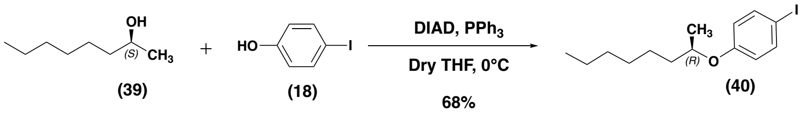

Experimental procedure:

To a stirring solution of compound **39** (2.13g, 16.36 mmol) and 4-iodophenol, **18** (3.0 g , 13.64 mmol) in dry THF (100.0 mL) was added DIAD (4.14 g, 20.45 mmol) and PPh_3 (5.36 g, 20.45 mmol) at 0 °C. The reaction mixture was stirred at 0 °C for 4 h under Ar-atmosphere. The reaction was monitored by TLC and quenched with aqueous $NaHCO_3$ solution (100.0 mL) and extracted two times with EtOAc and the combined organic layer was dried with anhydrous Na_2SO_4. After removal of the solvent the crude product was purified by flash chromatography to obtain 3.06 g of the pure product **40** as colorless liquid with 68% yield. ^{1}H NMR (500 MHz, $CDCl_3$) δ 7.56 – 7.49 (m, 2H, Ar-Ph-H), 6.69 – 6.62 (m, 2H, Ar-Ph-H), 4.29 (h, J = 6.1 Hz, 1H), 1.76 – 1.65 (m, 1H), 1.54 (ddt, J = 13.6, 10.9, 5.3 Hz, 1H), 1.47 – 1.32 (m, 2H), 1.28 (dd, J = 12.4, 5.6 Hz, 9H), 0.91 – 0.83 (m, 3H). ^{13}C NMR (101 MHz, $CDCl_3$) δ 14.13, 19.63, 22.63, 25.49, 29.28, 30.35, 31.81, 36.37, 74.12, 82.27, 118.24, 125.56, 138.23, 158.13.

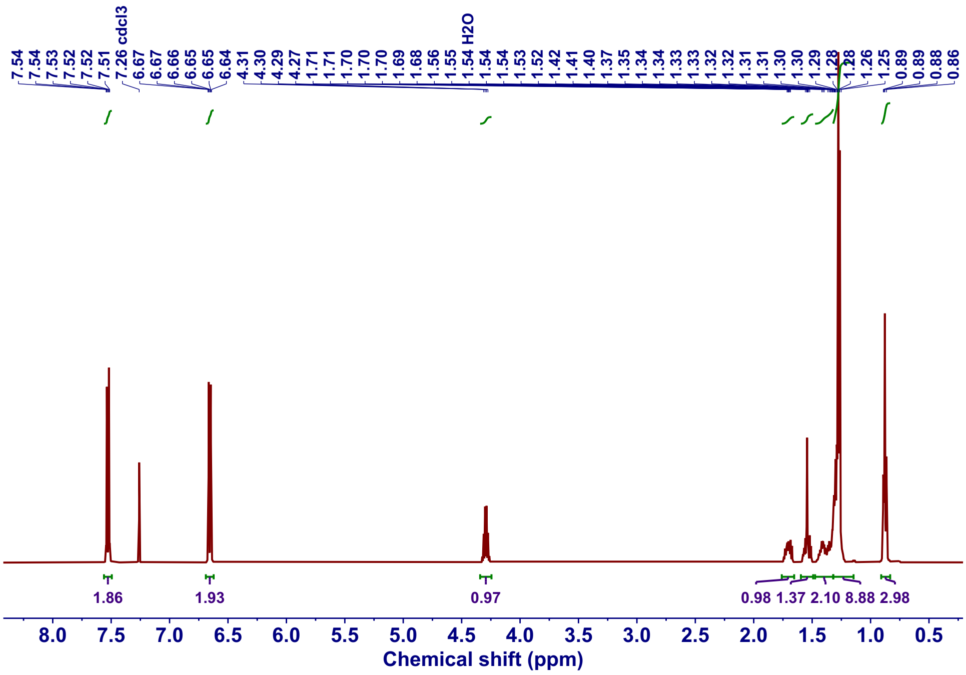

Figure S23a. ^{1}H-NMR spectrum of compound **40**.

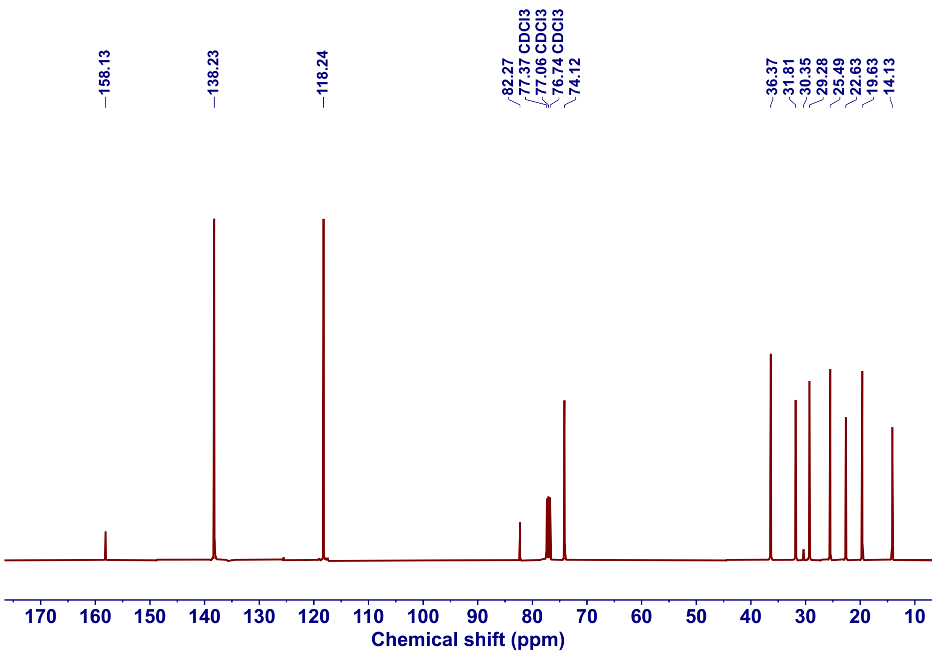

Figure S23b. ^{13}C-NMR spectrum of compound **40**.

S2.6.2. Synthesis of (R)-C1 chiral intermediate step-II

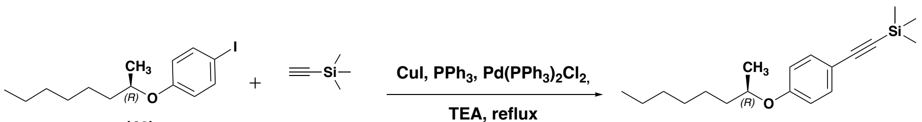

Experimental procedure:

To a stirring solution of compound **40** (2.7 g, 8.13 mmol) in triethylamine (30.0 mL) were added Pd(PPh$_3$)$_2$Cl$_2$ (150.2 mg, 0.21 mmol), CuI (80.0 mg, 0.43 mmol), and triphenylphosphine (60.4 mg, 0.43 mmol). The mixture was degassed by bubbling through Ar for 40 min and then trimethylsilylacetylene, **27** (1.32 mL, 9.24 mmol) was added slowly at room temperature. The mixture was stirred at room temperature for 6 h. The reaction mixture was diluted with dichloromethane (50 ml) and filtered through celite on a fritted disk funnel. The filtrate was washed twice with 1M HCl (50 mL), twice with H_2O (200 mL), and then dried over Na_2SO_4. The solvent was removed under vacuum, and the residue was purified by flash chromatography to obtain 2.25 g of pure compound **43** as a colorless liquid with 92% yield. ^{1}H NMR (400 MHz, CDCl$_3$) δ 7.42 – 7.34 (m, 2H, Ar-Ph-H), 6.83 – 6.74 (m, 2H, Ar-Ph-H), 4.35 (h, J = 6.1 Hz, 1H), 1.78 – 1.65 (m, 1H), 1.61 – 1.47 (m, 1H), 1.45 – 1.31 (m, 3H), 1.30 – 1.25 (m, 8H), 0.92 – 0.83 (m, 3H), 0.23 (d, J = 0.3 Hz, 9H). ^{13}C NMR (101 MHz, CDCl$_3$) δ 1.05, 14.10, 19.65, 22.61, 25.45, 29.26, 31.79, 36.37, 73.91, 92.21, 105.35, 114.79, 115.54, 133.48, 158.48.

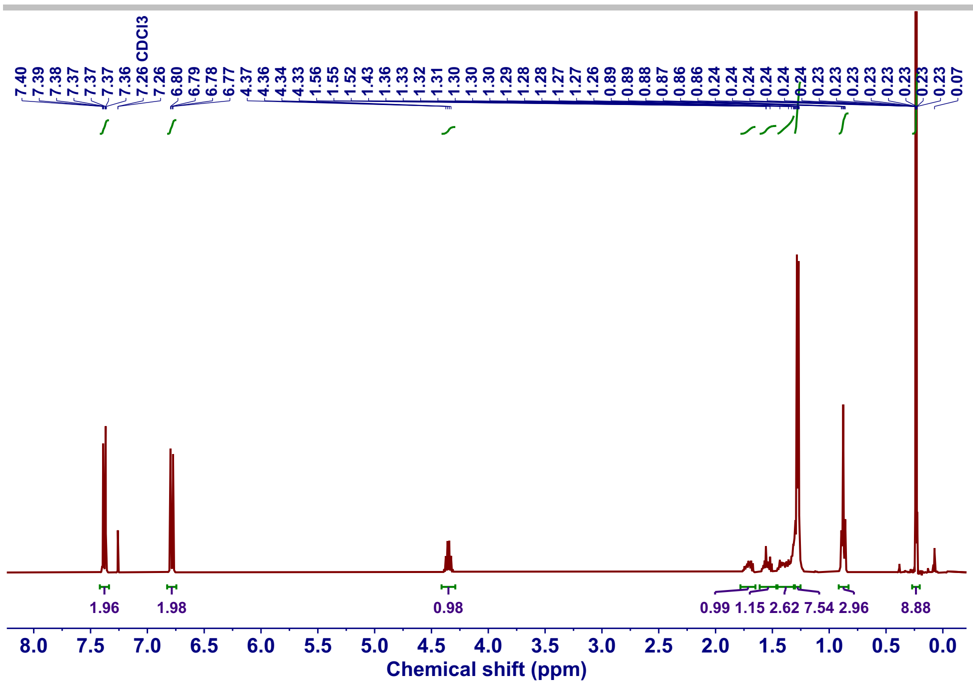

Figure S24a. ^{1}H-NMR spectrum of compound **43**.

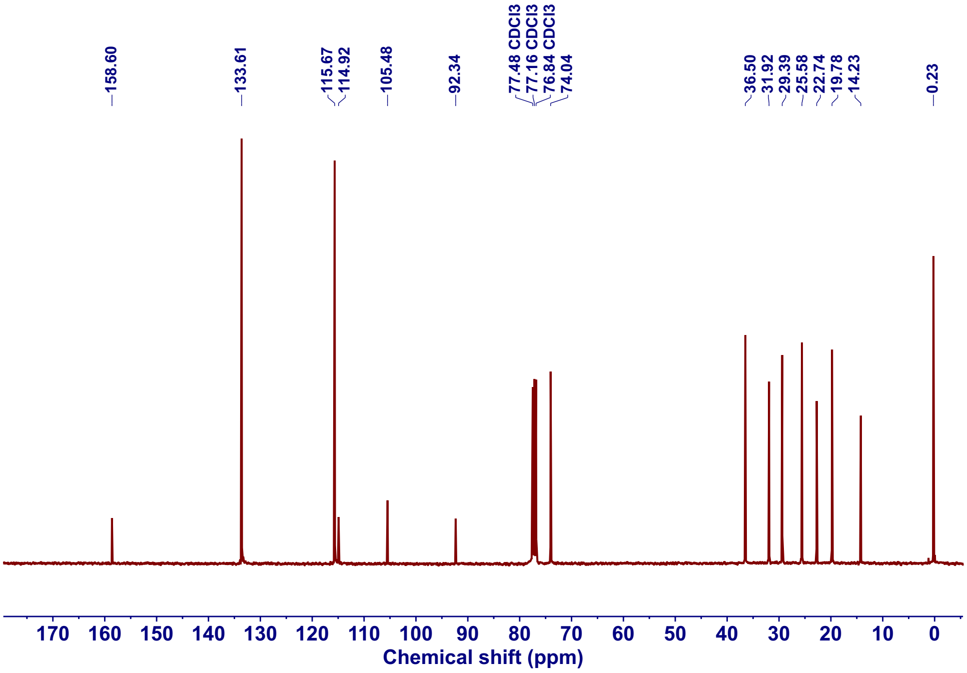

Figure S24b. ^{13}C-NMR spectrum of compound **43**.

S2.6.3. Synthesis of (R)-C1 chiral intermediate step-III

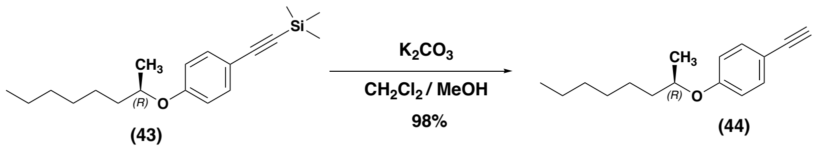

Experimental procedure:

To a stirring solution of compound **43** (2.40 g, 7.93 mmol) in dichloromethane (15.0 mL) was added K_2CO_3 (10.96 g, 79.33 mmol) and methanol (60.0 mL), the mixture was stirred at room temperature for 1.5 h. The reaction mixture was filtered, and the filtrate was washed with water (100 mL) and extracted with dichloromethane (200 mL). The organic layer was washed further with brine (100 mL) and dried over Na_2SO_4. After solvent was removed under vacuum the crude was purified by flash chromatography to obtain 1.8 g pure (*R*)-chiral intermediate **44** as a colorless liquid with 98% yield. ^{1}H NMR (400 MHz, $CDCl_3$) δ 7.45 – 7.37 (m, 2H, Ar-Ph-H), 6.85 – 6.77 (m, 2H, Ar-Ph-H), 4.36 (h, *J* = 6.1 Hz, 1H), 2.99 (s, 1H), 1.73 (dddd, *J* = 13.4, 9.9, 6.5, 5.0 Hz, 1H), 1.63 – 1.49 (m, 1H), 1.49 – 1.33 (m, 2H), 1.33 – 1.21 (m, 9H), 0.93 – 0.84 (m, 3H). ^{13}C NMR (101 MHz, $CDCl_3$) δ 1.05, 14.10, 19.66, 22.62, 25.48, 29.26, 31.80, 36.38, 73.93, 75.59, 76.35, 83.82, 113.67, 115.60, 133.62, 158.69.

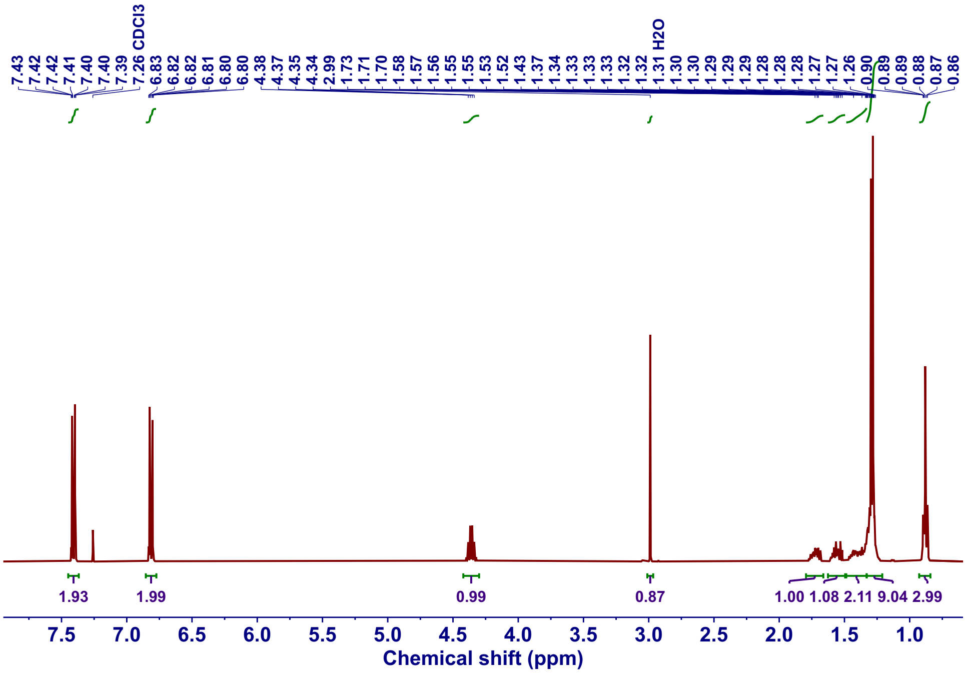

Figure S25a. ^{1}H-NMR spectrum of compound **44**.

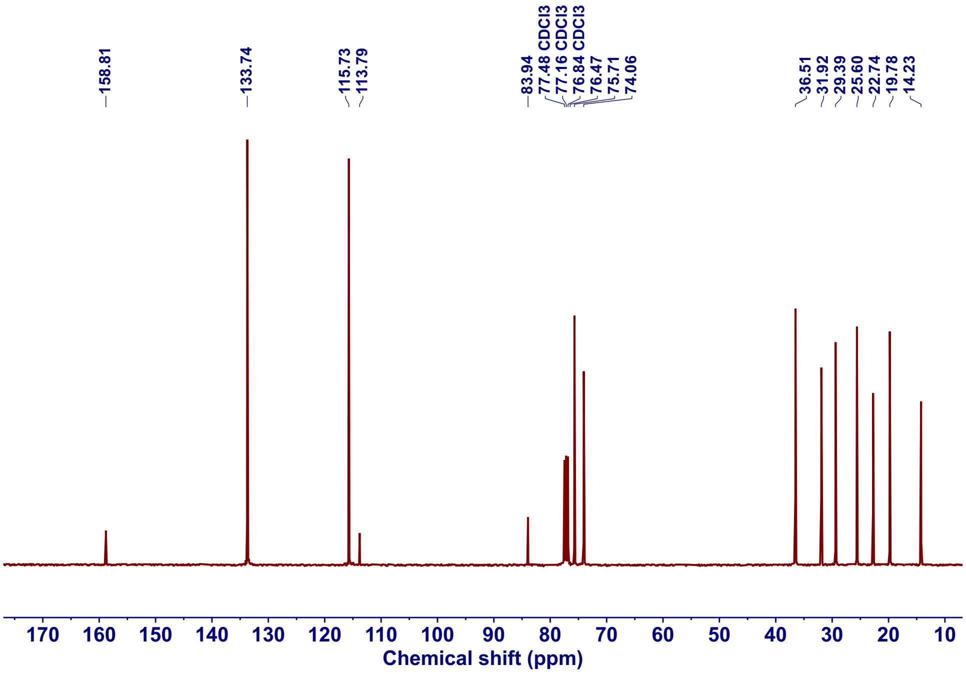

Figure S25b. ^{13}C-NMR spectrum of compound **44**.

*S2.6.4. Synthesis of (R)-C1/T12-SAc thioacetate (**45**):*

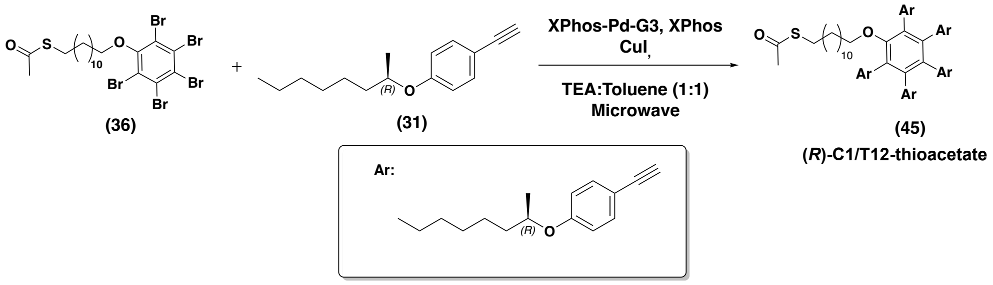

Experimental procedure:

To a mixture of X-Phos-Pd-G3 (16.0 mg, 0.014 mmol), Xphos (7.0 mg, 0.0137 mmol), copper iodide (5.2 mg, 0.02736 mmol), compound **36** (0.1 g, 0.137 mmol), and triethylamine (3.0 mL) was added compound **31** (160 mg, 0.711 mmol) in toluene (3.0 mL) dropwise under a nitrogen atmosphere. The mixture was stirred at microwave at 140 °C for 1 h 40 min and then cooled. To the cooled reaction mixture was added dichloromethane (100 mL). The mixture was washed twice with aqueous NH_4Cl (200 mL), twice with H_2O (200 mL), and dried over $MgSO_4$. After removal of the solvents, the residue was purified by column chromatography (*n*-hexane/dichloromethane 5:1) to yield 70 mg of **45** (yield = 35%) as an orange liquid. ^1H NMR (500 MHz, $CDCl_3$) δ 7.57 – 7.47 (m, 10H, Ar-Ph-H), 6.89 – 6.82 (m, 10H, Ar-Ph-H), 4.40 (p, *J* = 6.1 Hz, 5H), 4.33 (t, *J* = 6.4 Hz, 2H), 2.86 (t, *J* = 7.4 Hz, 2H), 2.31 (s, 3H), 1.94 – 1.87 (m, 2H), 1.75 (ddddd, *J* = 13.4, 10.2, 6.5, 5.1, 1.4 Hz, 5H), 1.63 – 1.54 (m, 11H), 1.36 – 1.23 (m, 64H), 0.93 – 0.83 (m, 18H). ^{13}C NMR (126 MHz, $CDCl_3$) δ 1.17, 14.23, 14.27, 19.84, 22.75, 25.64, 26.52, 28.99, 29.29, 29.31, 29.42, 29.65, 29.73, 29.77, 29.80, 29.85, 30.75, 30.78, 31.94, 36.58, 74.12, 74.13, 74.77, 83.64, 86.21, 86.74, 97.17, 99.27, 99.42, 115.29, 115.34, 115.55, 115.88, 115.89, 120.01, 124.02, 128.52, 133.30, 133.34, 133.49, 158.68, 158.80, 158.89, 159.97, 196.19. HRMS: m/z calcd. for $C_{100}H_{132}O_7{}^{32}S^{23}Na$, $C_{99}{}^{13}CH_{132}O_7{}^{32}S^{23}Na$, $[M+Na]^+$ = 1499.95860, 1500.96195; observed = 1499.9548, 1500.9583.

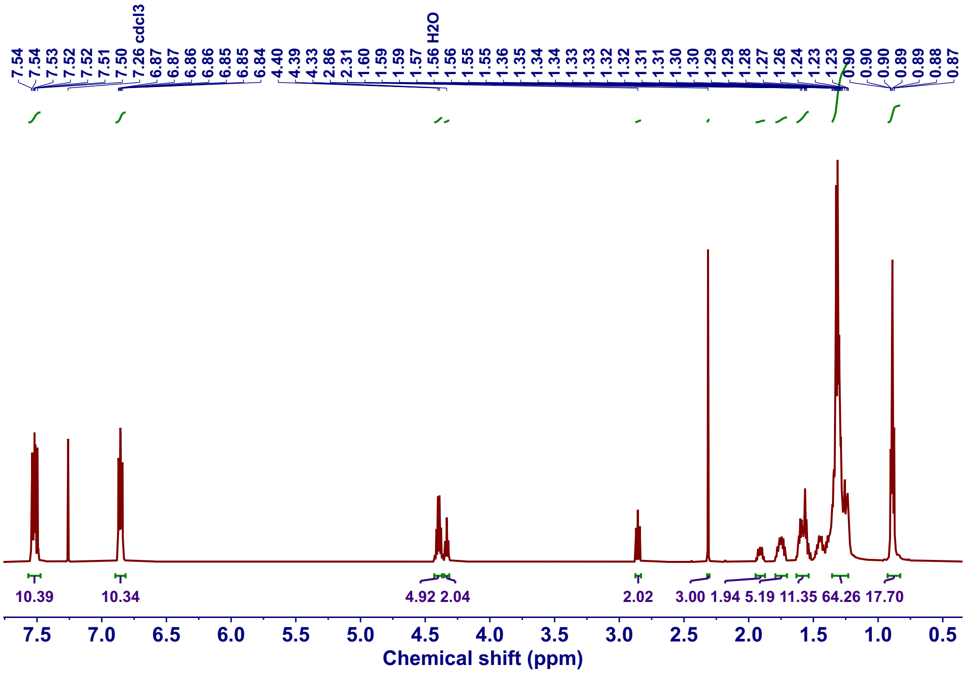

Figure S26a. ^1H-NMR spectrum of compound **45**.

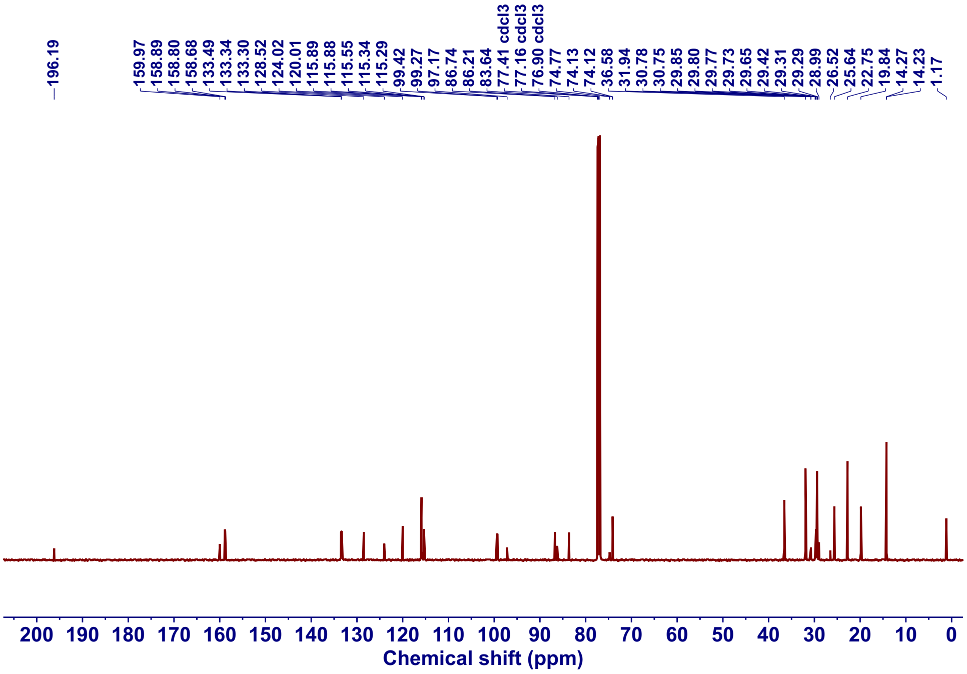

Figure S26b. ^{13}C-NMR spectrum of compound **45**.

*S2.6.5. Synthesis of (R)-C1/T12-SH thiol (**46**):*

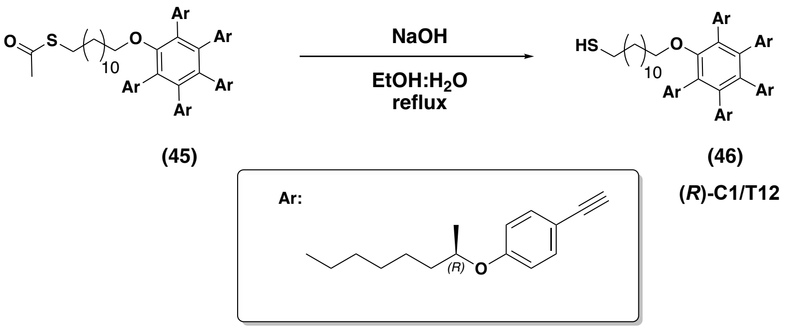

Experimental procedure:

To a mixture of compound **45** (0.15 g, 0.096 mmol) in ethanol (10.0 mL) was added aqueous NaOH (7.0M, 5.0 mL) dropwise. The mixture was refluxed for 4 h and then cooled. To the cooled reaction mixture was added dichloromethane (50 mL). The mixture was washed twice with H_2O (50 mL) and dried over $MgSO_4$. After removal of the solvents the residue was purified by column chromatography using neutral alumina as a stationary phase, followed by preparative TLC (*n*-hexane/dichloromethane 5:1) to yield 20 mg of **46** (yield = 41%) as an orange liquid. ^{1}H NMR (400 MHz, $CDCl_3$) δ 7.57 – 7.46 (m, 10H, Ar-Ph-H), 6.90 – 6.80 (m, 10H, Ar-Ph-H), 4.40 (p, *J* = 6.1 Hz, 5H), 4.33 (t, *J* = 6.3 Hz, 2H), 2.69 – 2.63 (m, 2H), 1.91 (p, *J* = 6.5 Hz, 2H), 1.75 (ddd, *J* = 6.5, 3.4, 1.4 Hz, 2H), 1.60 – 1.56 (m, 5H), 1.52 – 1.38 (m, 10H), 1.35 – 1.21 (m, 64H), 0.94 – 0.83 (m, 18H). ^{13}C NMR (101 MHz, $CDCl_3$) δ 1.17, 14.25, 19.84, 22.76, 25.64, 26.55, 28.75, 29.42, 29.47, 29.74, 29.82, 29.85, 30.76, 31.94, 36.57, 39.25, 74.10, 74.77, 83.62, 86.20, 86.72, 97.16, 99.26, 99.41, 115.27, 115.31, 115.53, 115.87, 120.01, 124.02, 128.50, 133.30, 133.34, 133.48, 158.66, 158.78, 158.87, 159.96.

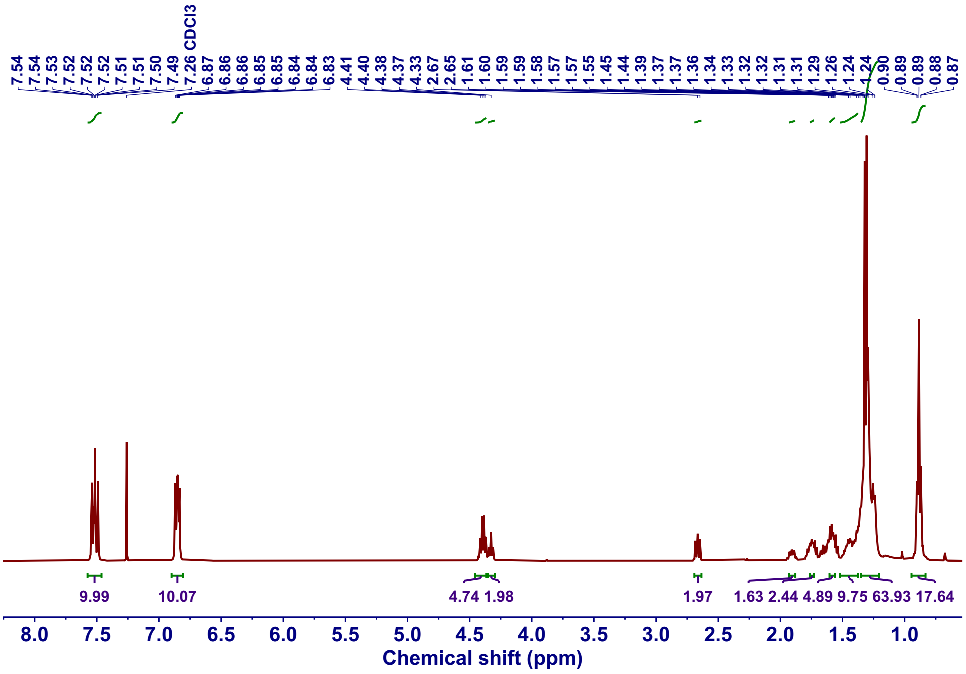

Figure S27a. ^{1}H-NMR spectrum of compound **46**.

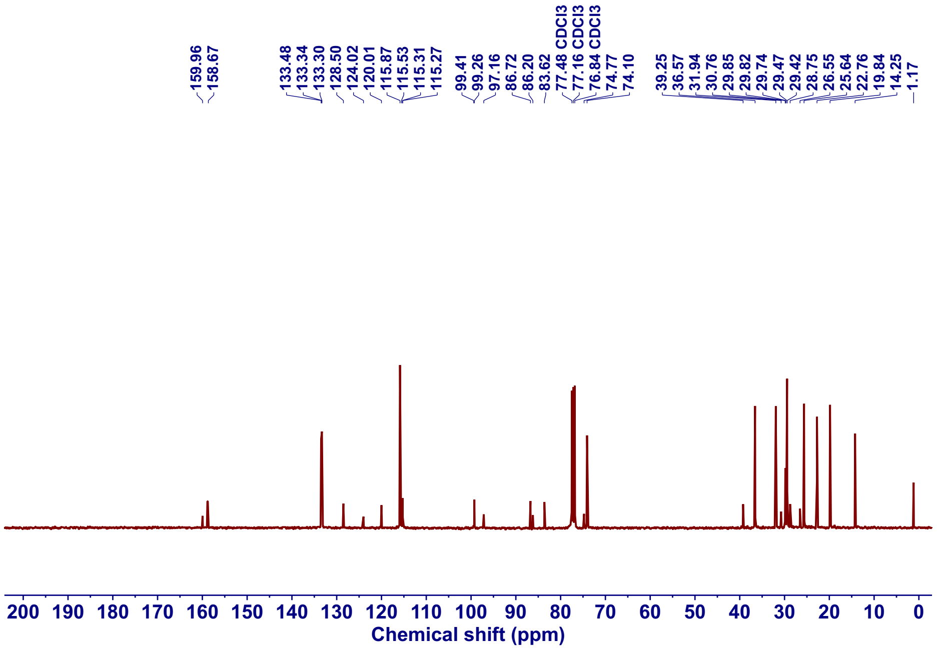

Figure S27b. ^{13}C-NMR spectrum of compound **46**.

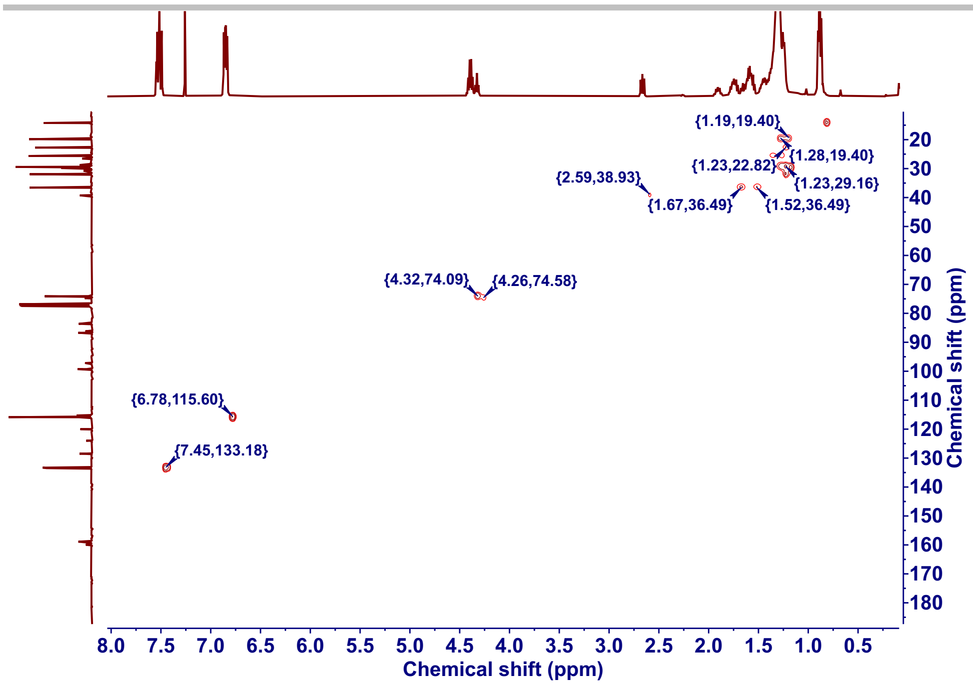

Figure S27c. {^{1}H -^{13}C}-HSQC spectrum of compound **46**.

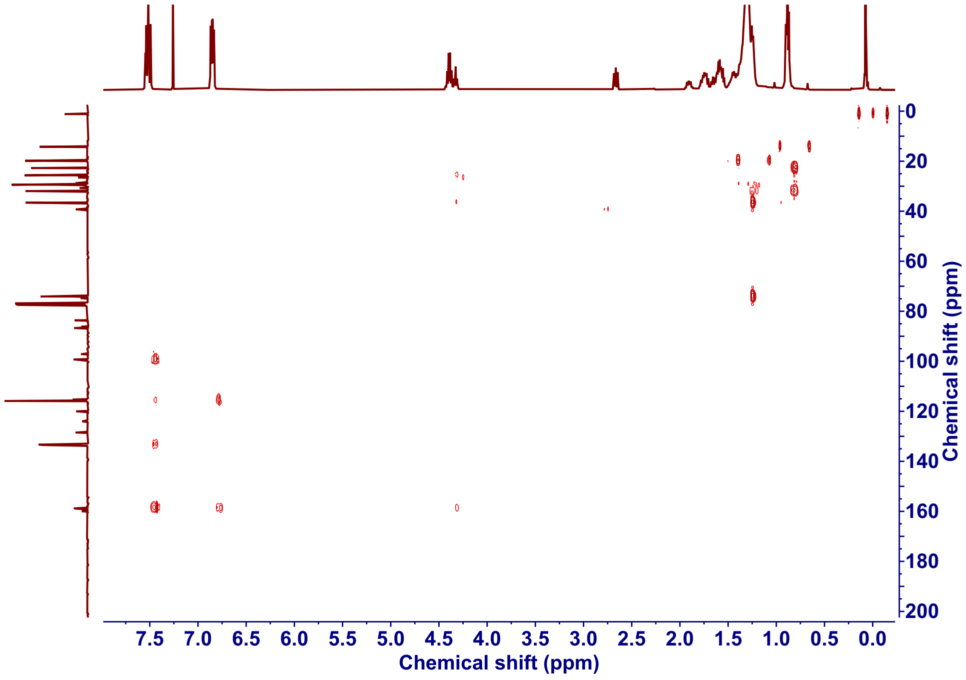

Figure S27d. {^{1}H -^{13}C}-HMBC spectrum of compound **46**.

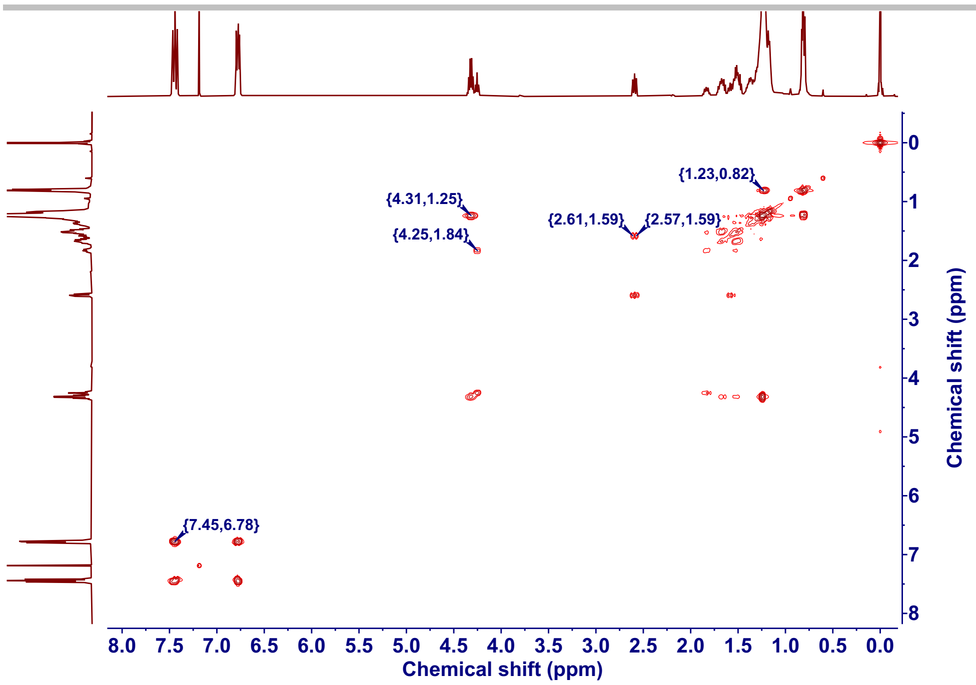

Figure S27e. {^{1}H -^{1}H}-COSY spectrum of compound **46**.

S3. X-ray diffraction (XRD) data of N_D phase

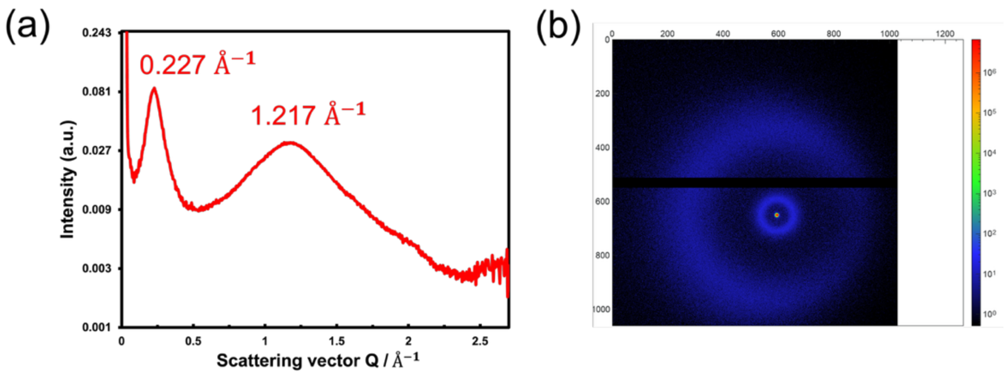

Figure S28. XRD analysis data showing: (a) the azimuthally averaged intensity (a.u.) vs. wave vector q (Å^{-1}) of the medium angle region after slow cooling from the isotropic liquid phase taken at T = 40 °C, and (b) the corresponding 2-D XRD pattern (q-values correspond to 27.7 Å for the inter-disc-distance along the disc plane and 5.16 Å originating from the liquid-like aliphatic side chains and inter-disc distances along the disc-normal).

S4. Calculations of IPR_{2D} using molecular envelope for 1 and the chiral organic solutes

Precise values for IPR_{2D} were calculated from the energy-minimized molecular structure of **1** assuming standard van der Waals atomic radii to build a molecular envelope representation, which is then projected on the principal XY-planes of the tensor of inertia Eigenframe, of interest for stacking and calculating the convex hull perimeter and enclosed area. The results obtained for **1** as well as the chiral organic solutes (*R*)-C3/T8-SAc, (*S*)-C1/T8-SAc, and (*R*)- or (*S*)-C1/T12-SAc, using a simple Python code (available from the authors), are shown in Figure S29 below.

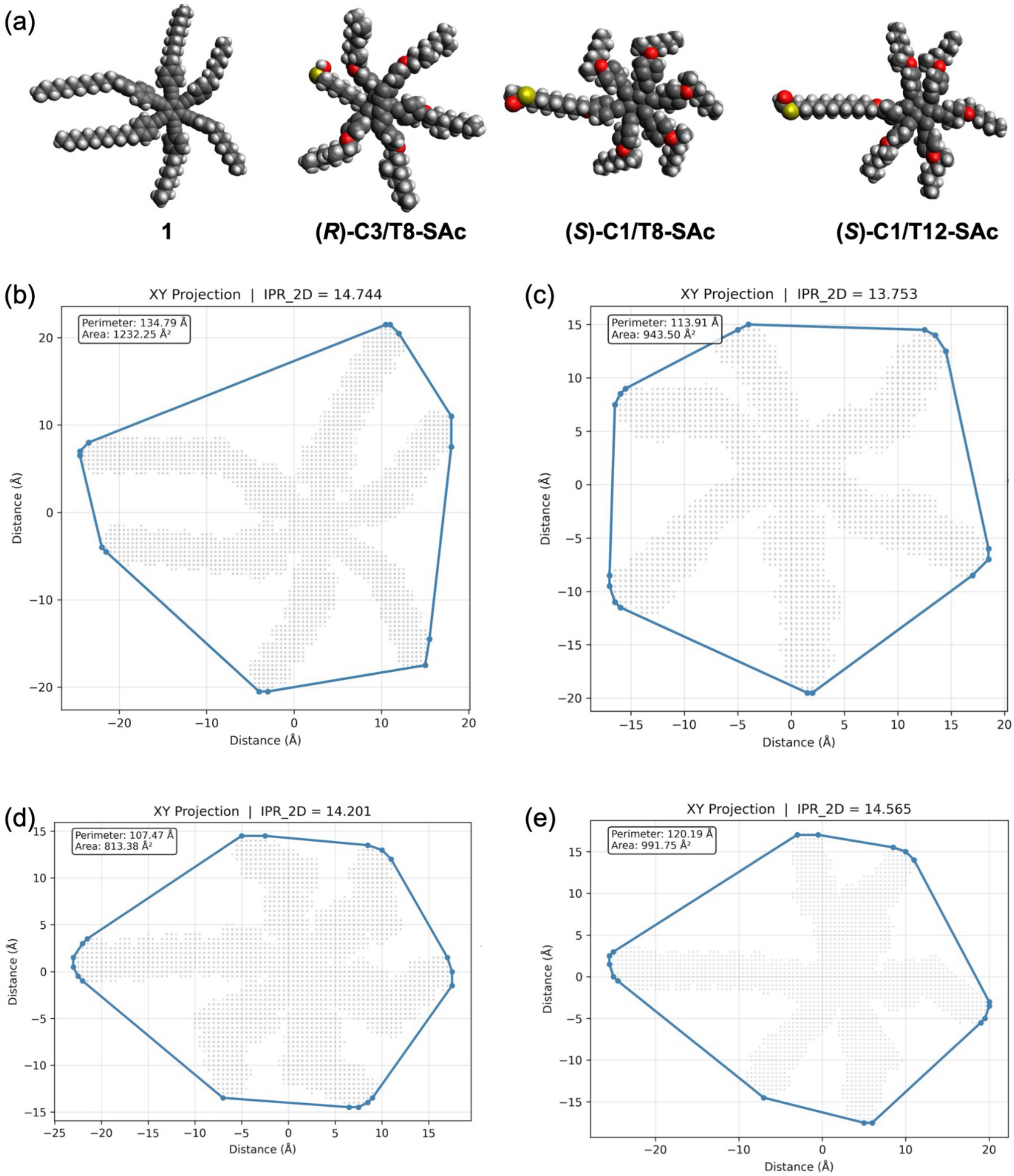

Figure S29. Calculations of IPR_{2D} using molecular envelope: (a) energy-minimized molecular structures used to calculate IPR_{2D} based on a molecular envelope for (b) **1**, (c) (*R*)-C3/T8-SAc, (d) (*S*)-C1/T8-SAc, and (e) (*S*)- or (*R*)-C1/T12-SAc.

S5. Synthesis of gold nanoshapes

S5.1. Synthesis of gold nanorods (GNRs)[40]

Preparation of seed solution:
5.0 mL of 0.5 mM $HAuCl_4$ was mixed with 5 mL of 0.2 M CTAB solution in a 20.0 mL scintillation vial. To this, 0.6 mL of fresh 0.01 M $NaBH_4$, diluted to 1.0 mL with water, was added under vigorous stirring at 1,200 rpm. The solution color was observed to change from yellow to brownish yellow. Stirring was stopped after 2 min. The seed solution was aged at room temperature for 30 min before use.

Preparation of growth solution:
2.8 g of CTAB and 0.4936 g NaOL were dissolved in 100.0 mL of warm water (~50 °C) in a 250 mL Erlenmeyer flask. The solution was allowed to cool down to 30 °C. 9.6 mL 4 mM $AgNO_3$ solution was added and the solution was stirred at 400 rpm at 30 °C for 15 minutes followed by 100 mL of 1 mM $HAuCl_4$ solution were added. The solution became colorless after 90 min of stirring at 700 rpm. The pH was adjusted by adding 0.84 mL of HCl (37% in H_2O, 12.1 M). After 15 min of slow stirring at 400 rpm, 0.5 mL of 0.064 M ascorbic acid was added, and the mixture was stirred vigorously for 30 s. Finally, 0.32 mL of seed solution was injected into the growth solution. The solution was stirred for 30 s and then left undisturbed at 30 °C for 12 h for NR growth.

Purification of GNRs:
The resulting dispersion was evenly divided into 50 mL centrifuge tubes and subjected to centrifugation at 12,000 rpm for 10 min. This washing step was repeated three times, and the supernatant was discarded after each cycle. The purified GNRs were redispersed in deionized water for further use.

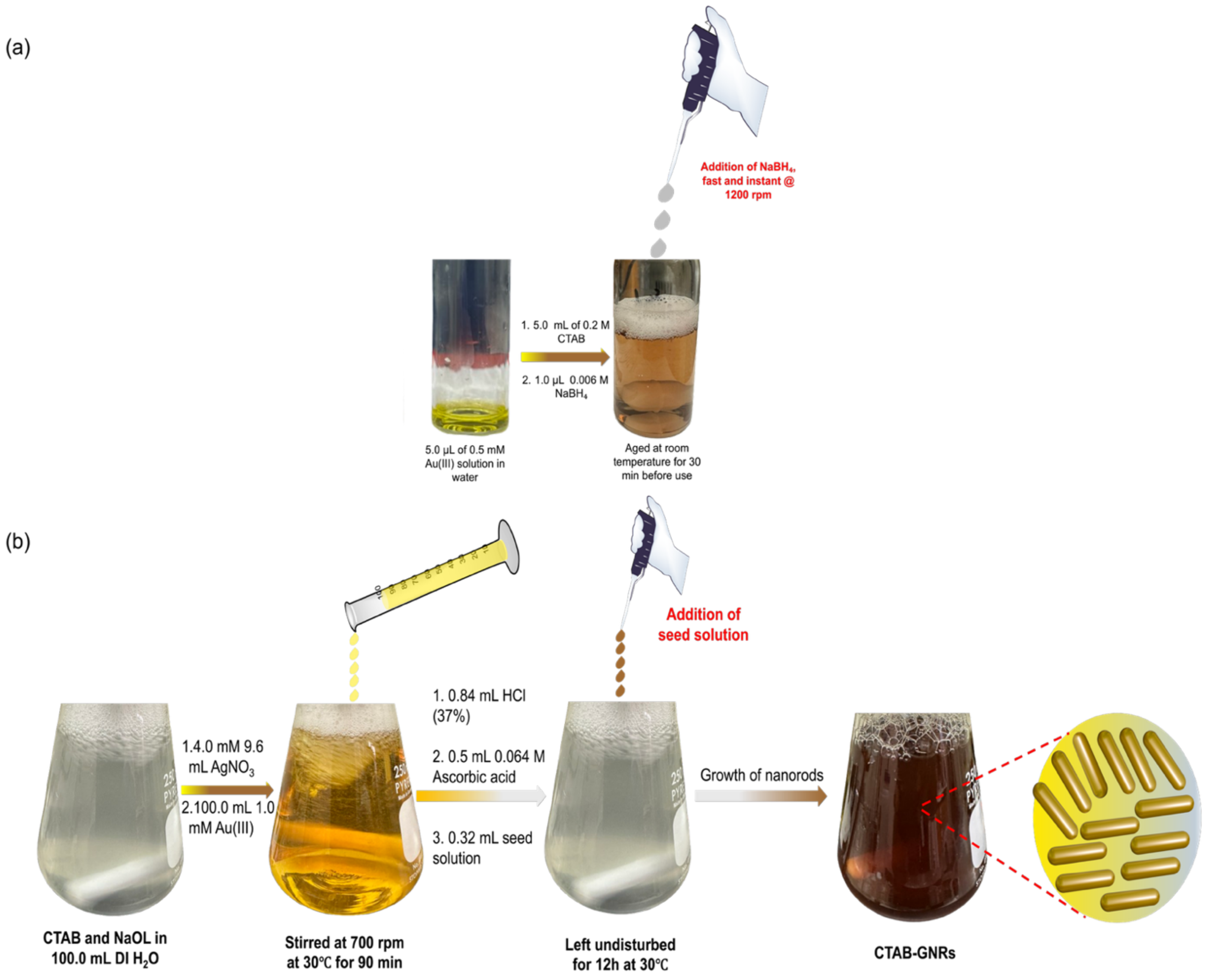

Figure S30. Schematic representation for the synthesis of the GNRs; (a) preparation of gold nanoparticle seeds and (b) preparation of growth solution.

S.4.1.1. TEM images of GNRs

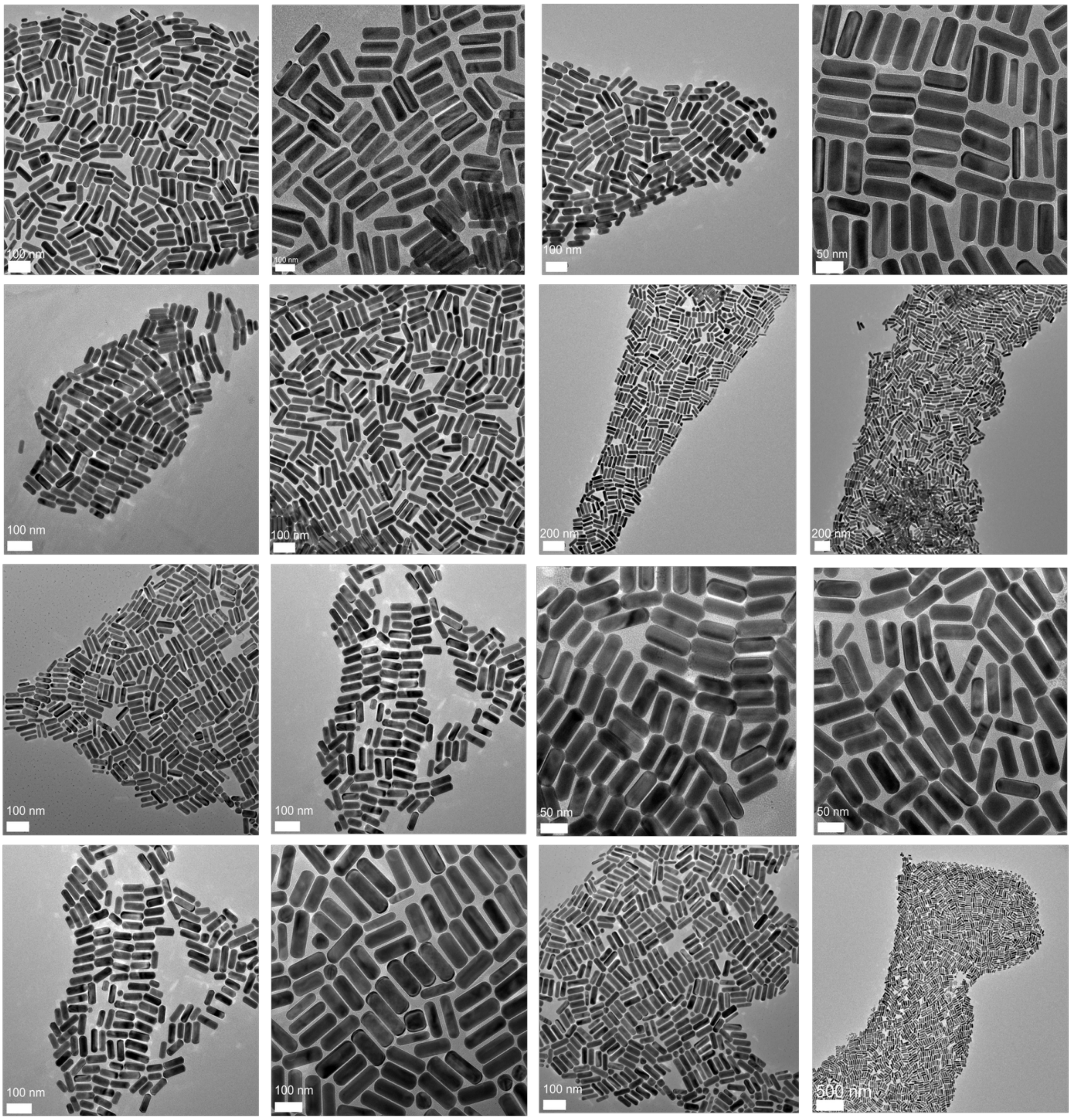

Figure S31. Transmission electron microscopy (TEM) images of the CTAB-coated GNRs with average total length l_{total} = 95.9 nm and diameter d = 29.4 nm.

S.5.2. Synthesis of gold nanoprisms (GNPRs)[41]

Preparation of seed solution:

A 20 mL disposable glass vial equipped with a magnetic stir bar was placed in a sand bath maintained at 30 °C and stirred at 300 rpm. Once the temperature was stabilized, 4.7 mL of 0.1 M CTAC (13.23 mL of stock 25% CTAC in 86.77 mL DI H_2O) solution was added, followed by 25.0 µL of 0.05 M $HAuCl_4 \cdot 3H_2O$ (84.9 mg in 5.0 mL DI H_2O). After stirring for 3 min, 300.0 µL of freshly prepared, ice-cold 0.01 M $NaBH_4$ (7.6 mg in 20 mL DI H_2O) was rapidly injected under vigorous stirring at 1,200 rpm. The seed dispersion was stirred at

250 rpm for 2 h at 25 °C to consume residual borohydride while maintaining gentle stirring to minimize foam formation. Freshly prepared, chilled $NaBH_4$ solution was critical to ensure reproducible nucleation.

Preparation of first growth solution:
A 50 mL Erlenmeyer flask was placed on a hotplate maintained at 25 °C. To this, 1.8 mL of 0.1 M CTAC solution was added, followed by 8.0 mL of deionized water to reduce the surfactant concentration. The mixture was stirred for 5 min to obtain a homogeneous solution. Subsequently, 40.0 μL of 0.05 M $HAuCl_4 \cdot 3H_2O$ solution and 15.0 μL of 0.01 M NaI (15 mg in 10 mL DI H_2O) were added sequentially under gentle stirring.

Preparation of second growth solution:
A 100 mL Erlenmeyer flask was placed on a hotplate maintained at 25 °C. To this, 40.0 mL of 0.05 M CTAC solution (prepared in step 2 and diluted to half of its original concentration) was added and stirred for 5 min to obtain a homogeneous solution. Subsequently, 500.0 μL of 0.05 M $HAuCl_4 \cdot 3H_2O$ and 300.0 μL of 0.01 M NaI solutions were sequentially introduced under gentle stirring.

Synthesis of gold nanoprism solution:
Prior to the main growth step, the seed solution was diluted tenfold with deionized water. An aqueous 0.1 M ascorbic acid solution (176.0 mg in 10.0 mL DI H_2O) was freshly prepared and added in portions of 40.0 μL and 400.0 μL to the first and second growth solutions. Upon gentle mixing, the initially colorless dispersions gradually turned light brown to yellow, indicating the reduction of Au(III) to Au(I). Subsequently, 200.0 μL of the diluted seed solution was added to the first growth solution, and 3.2 mL of this mixture was immediately transferred into the second growth solution using a 5.0 mL plastic syringe under gentle agitation.

Purification and storage of gold nanoprism solution:
After 5 h of reaction, 11.0 mL of the CTAC stock (25% v/v CTAC) solution was added, and the mixture was gently shaken. The resulting dispersion was transferred to a 100.0 mL glass cylinder and allowed to stand undisturbed for 15 h. A greenish-blue precipitate formed along the inner walls of the cylinder, corresponding to the gold nanoprisms. The adherent nanoprisms were rinsed with 10.0 mL of deionized water, and mild shaking facilitated their redispersion into a colloidal aqueous suspension. The obtained dispersion was transferred into 50 mL centrifuge tubes and centrifuged at 12,000 rpm for 10 min. The supernatant was carefully decanted, and the purified nanoprisms were washed twice with deionized water and finally redispersed for further use.

S5.2.1. TEM images of GNPRs

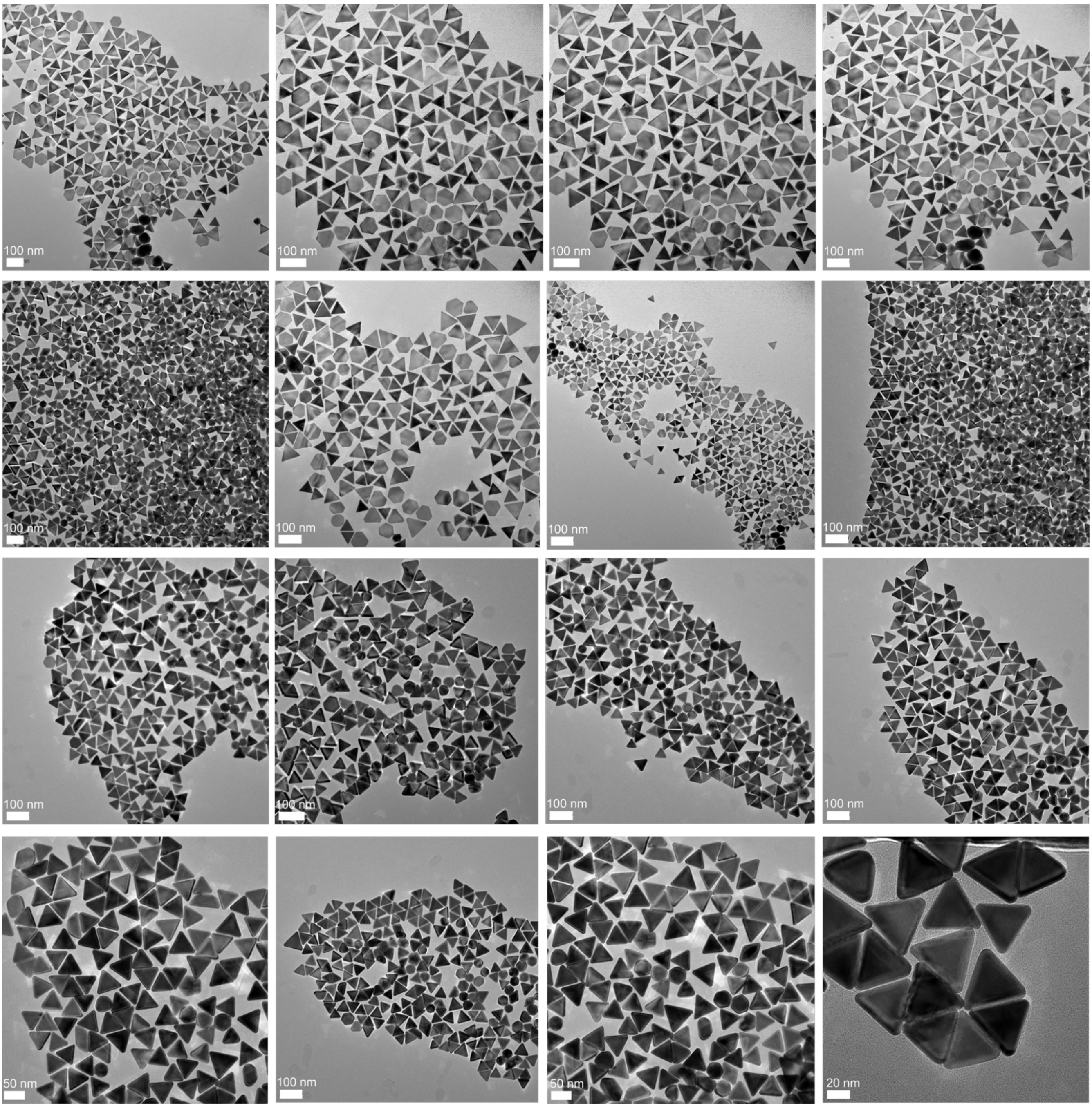

Figure S32. Transmission electron microscopy (TEM) images of the CTAC-coated triangular GNPRs with average length of the sides l_s = 50.2 nm and average height H = 26.5 nm.

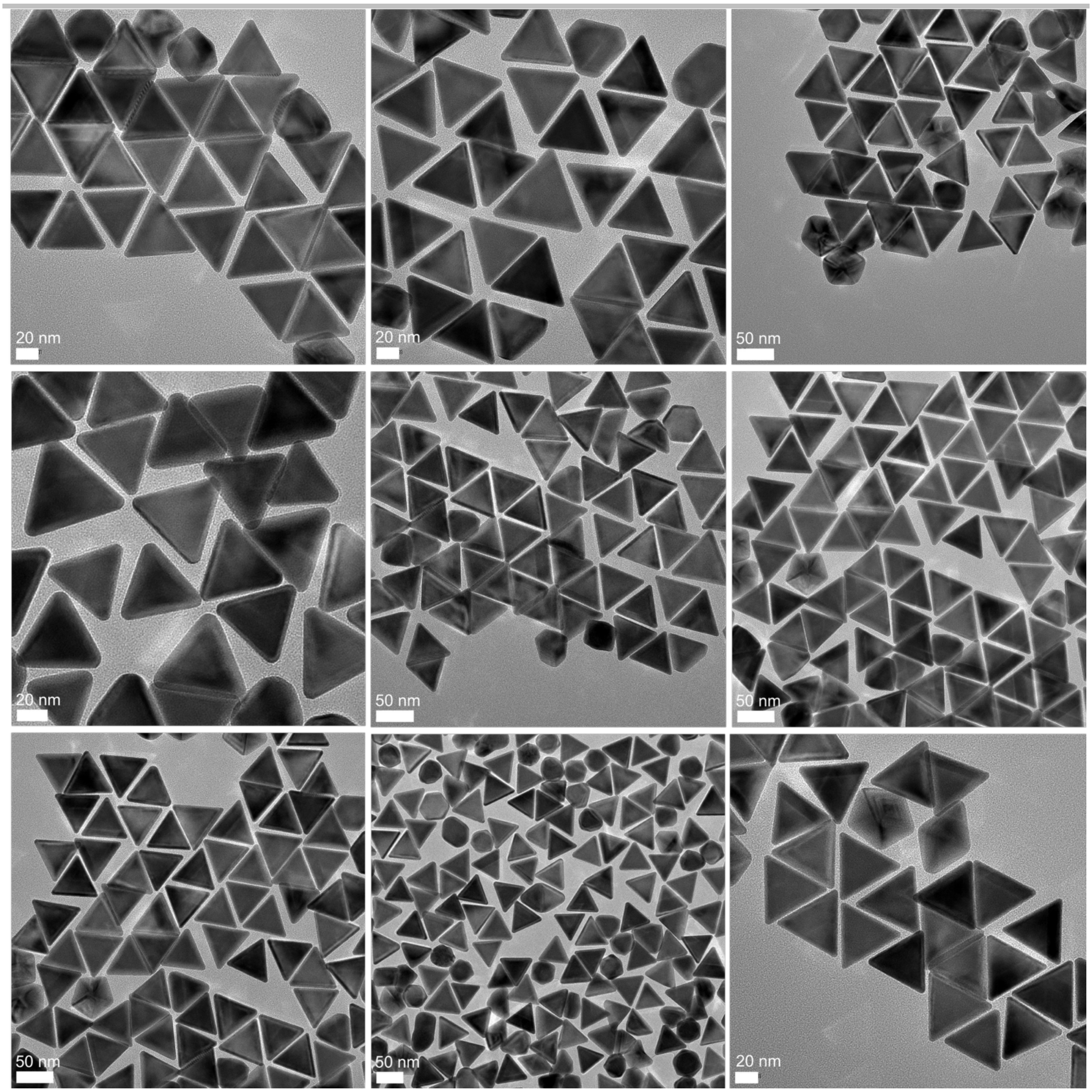

Figure S32 continued. Transmission electron microscopy (TEM) images of the CTAC-coated triangular GNPRs with average length of the sides l_s = 50.2 nm and average height H = 26.5 nm.

S5.3. Synthesis of gold nanodiscs (GNDs)[18c]

To synthesize GNDs, triangular gold nanoprisms were first prepared and subsequently subjected to controlled oxidative etching. The anisotropic oxidation of the as-grown hexagonal GNPRs was carried out by adding 200.0 µL of 1.0 M HCl and 300.0 µL of 6.0 wt.% H_2O_2 to 50.0 mL of the GNPR dispersion. The progress of the oxidation was monitored by recording the extinction spectra at regular time intervals. The gradual disappearance of the in-plane quadrupolar plasmon resonance band indicated the transformation of GNPRs into GNDs. Circular GNDs with an average diameter of approximately 80.0 nm were obtained following oxidation, centrifugation, and redispersion of the precipitated products in deionized water. By precisely tuning the oxidation duration, GNDs with nearly constant lateral dimensions but variable thicknesses were obtained.

S5.3.1. TEM images of GNDs

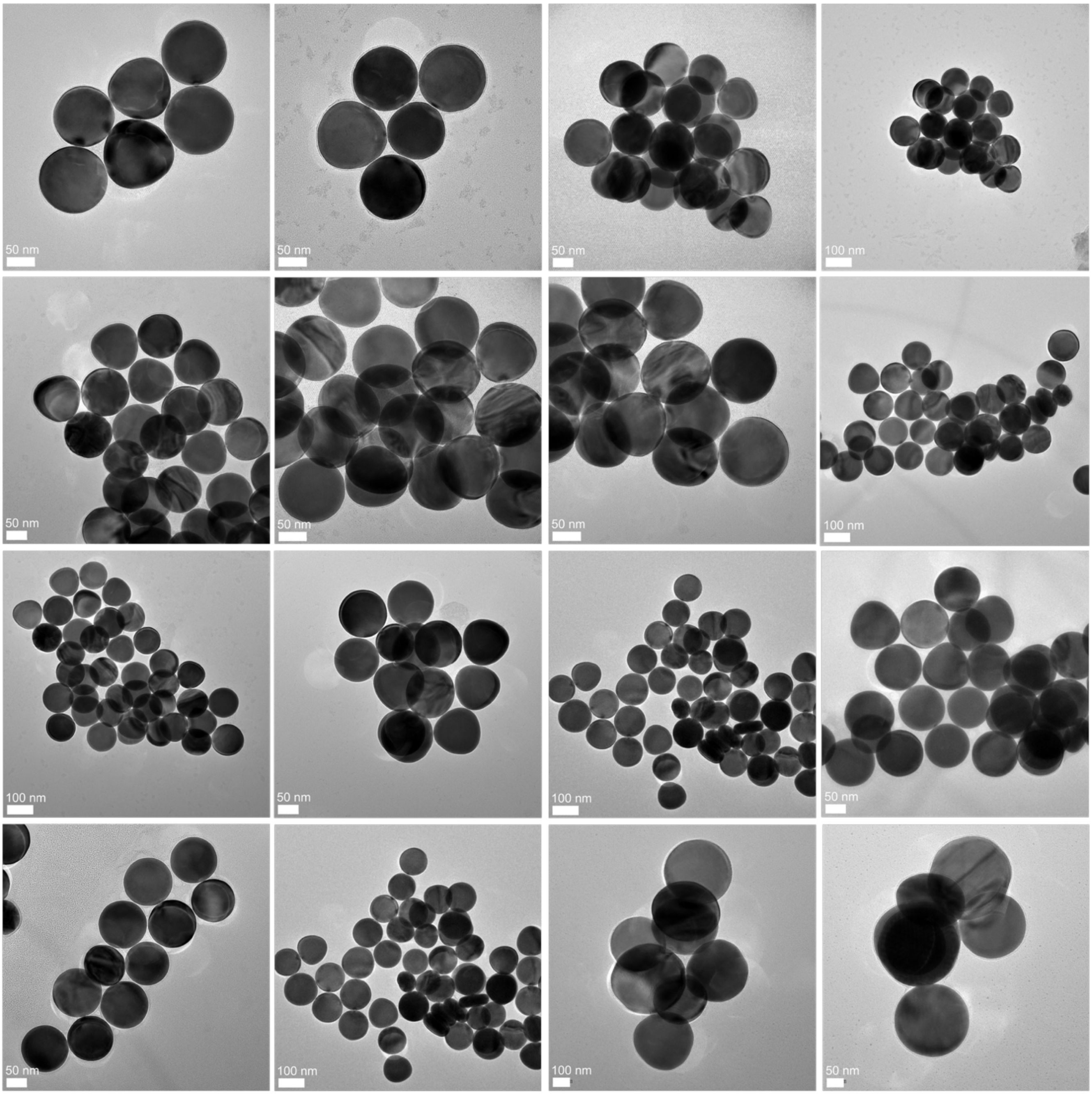

Figure S33. Transmission electron microscopy (TEM) images of the CTAB-coated GNDs with average diameter d = 79.6 nm and a height H = 29.9 nm.

S6. Surface functionalization of gold nanoshapes

S6.1 UV-vis and TEM

The CTAB/CTAC-coated gold nanoshape solution was centrifuged multiple times to eliminate excess surfactants and other solution components, then redispersed in 2.0 mL of DI water. This aqueous gold nanoshape solution was added dropwise to a stirred solution of 100 mg of (*S*)-C1/T12-SH in 50.0 mL (final ligand concentration 2.0 mg/mL and absorbance of gold nanoshape solution was also set at 2.0) of dry THF under nitrogen protection.[42] The reaction mixture color change was observed depending on the gold nanoshapes (brown for GNRs, bluish green for GNPRs and green for GNDs). Stirring continued at room temperature for 24 h. The mixture was then evaporated to dryness under reduced pressure. Chloroform was added to redisperse the functionalized nanoshapes, followed by multiple centrifugations (at 12,000 rpm for 16 minutes) to remove excess organic thiol ligands. This process was repeated three times to ensure thorough encapsulation of the gold nanoshapes with thiol molecules through strong covalent Au-S bonds. Finally, surface modified gold nanoshapes were redispersed in chloroform.

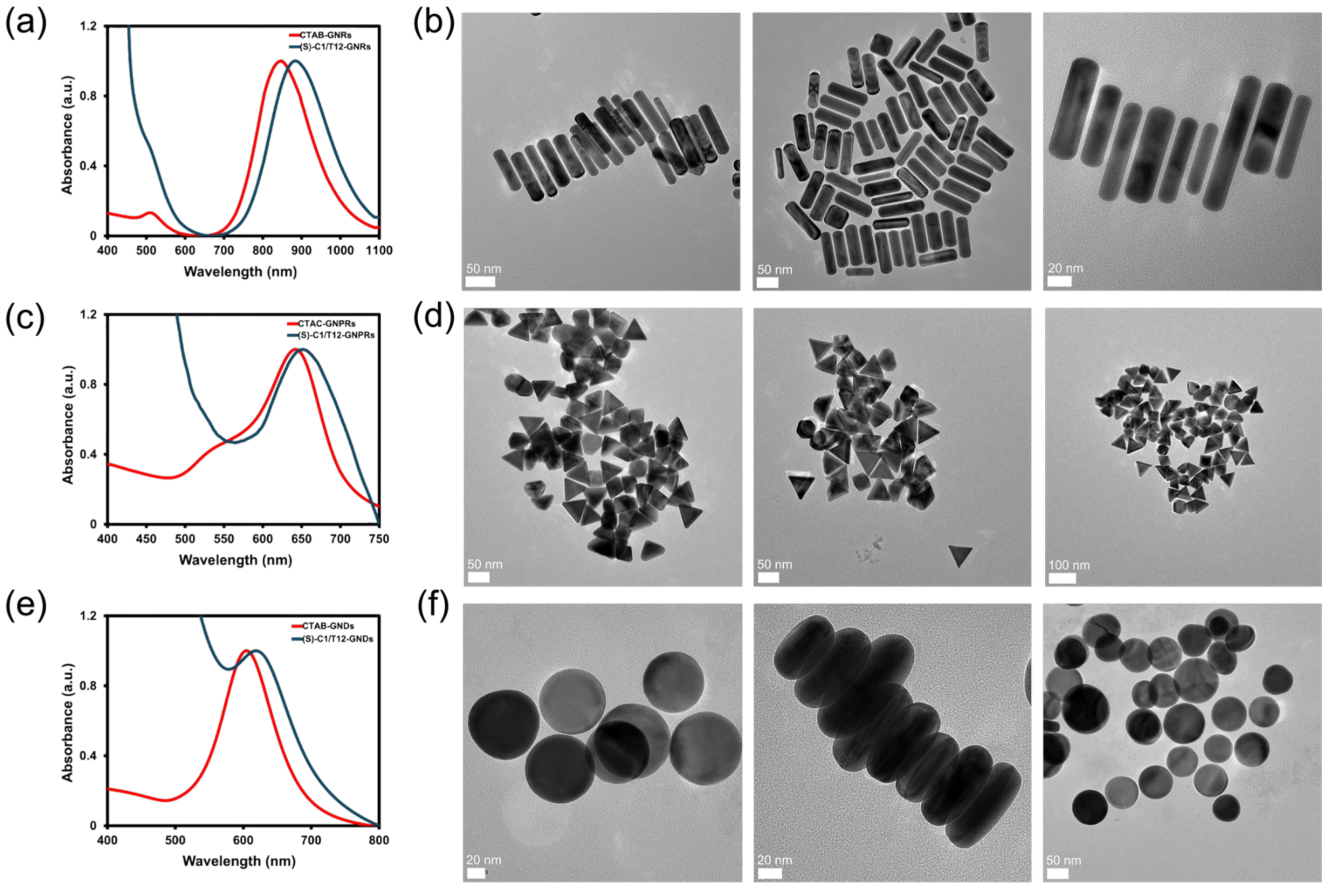

Figure S34. (a, c, e) Vis-NIR spectra of: (a) CTAB-capped GNRs (red), (*S*)-C1/T12-S−capped GNRs (blue), (c) CTAC-capped GNPRs (red), (*S*)-C1/T12-S−capped GNPRs (blue), (e) CTAB capped GNDs (red), (*S*)-C1/T12-S−capped GNDs (blue), all (*S*)-C1/T12-S−capped nanoshapes spectra recorded in chloroform solvent. (b, d, f) Transmission electron microscopy (TEM) images of: (b) (*S*)-C1/T12-S−capped GNRs after ligand exchange, (d) (*S*)-C1/T12-S−capped GNPRs after ligand exchange, and (f) (*S*)-C1/T12-S−capped GNDs after ligand exchange (the center image, as one example, permitted measurement of the average GND height).

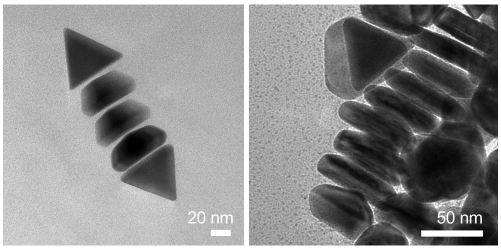

Figure S34 continued. Transmission electron microscopy (TEM) images of: (*S*)-C1/T12-S−capped GNPRs after ligand exchange showing some of them side-on allowing for measuring their average height H = 26.5 nm.

S6.2 ^{1}H-NMR spectroscopy

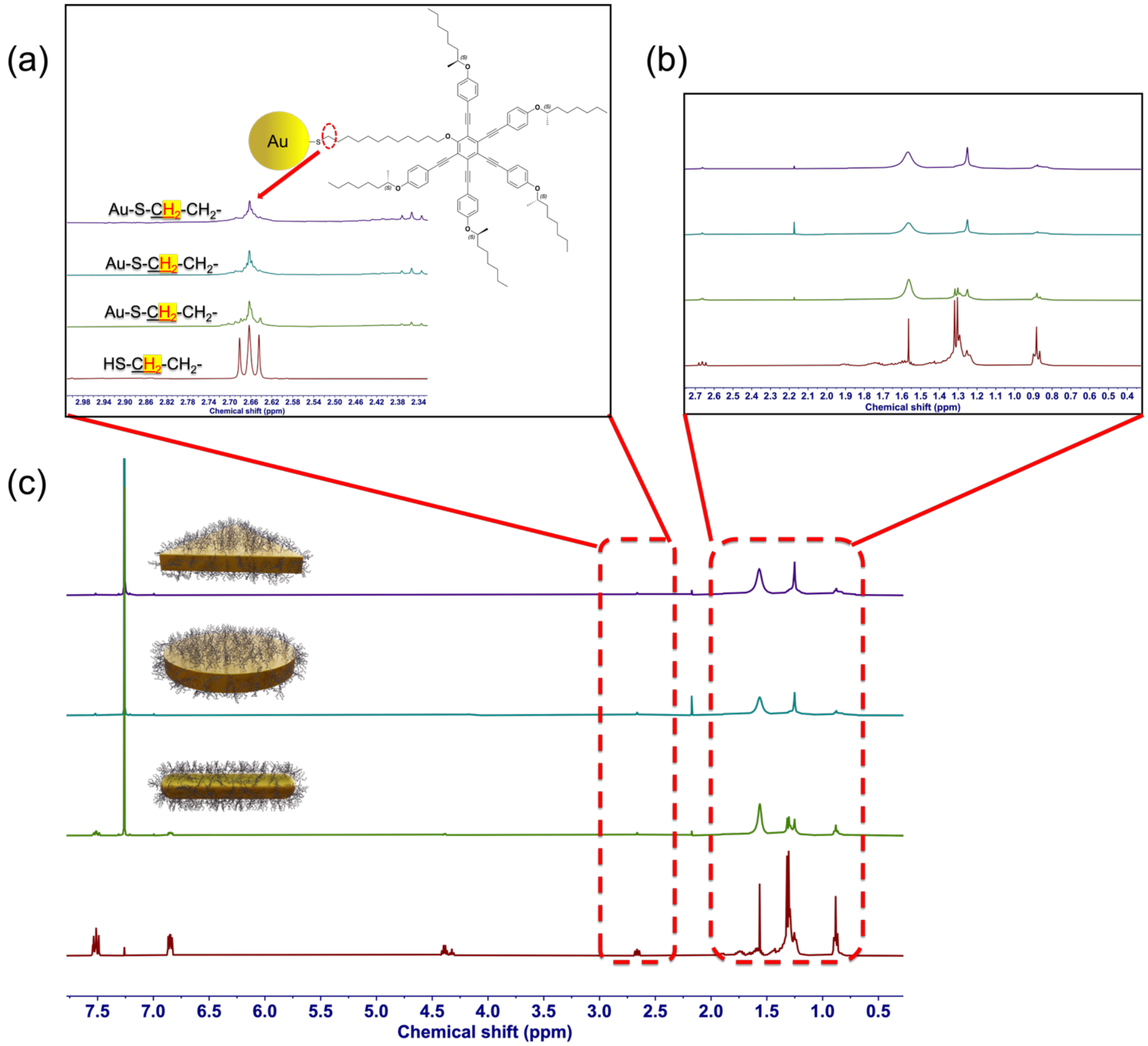

Figure S35. Comparative ^{1}H-NMR spectra of pristine (*S*)-C1/T12-SH (red) and (*S*)-C1/T12-S−capped gold nanoshapes, including GNRs (green), GNDs (cyan), and GNPRs (purple), recorded in $CDCl_3$. (a) Expansion highlighting the methylene protons adjacent to the thiol group (-CH_2-S), which appear as a distinct triplet in the free ligand however appear broadened upon coordination to each nanoshapes, (b) spectral region 0.4 – 2.7 ppm for aliphatic region, showing the transition from well-resolved multiplets in the pristine ligand to broadened signals for all nanoparticle-bound samples, consistent with restricted ligand mobility on the gold surface; the chemical shift positions remain unchanged, and (c) full comparative ^{1}H-NMR spectra of (*S*)-C1/T12-SH and its corresponding gold nanoshapes (GNRs, GNDs, GNPRs). All nanoshape-bound ligands show pronounced and uniform peak broadening compared to the free ligand, arising from ligand immobilization on the nanoparticle surface. Upon formation of a self-assembled monolayer (SAM), the alkyl chains become tightly packed, and protons located near the Au-core experience accelerated spin–spin (T_2) relaxation due to strong dipolar interactions and restricted segmental mobility. As a result, all ligand protons in close proximity to the gold surface-particularly those along the longitudinal facets appear as significantly broadened resonances in the nanoshape-bound spectra.

S6.3 Thermogravimetric analysis (TGA) of (*S*)-C1/T12-S−capped gold nanoshapes & calculations of surface coverage

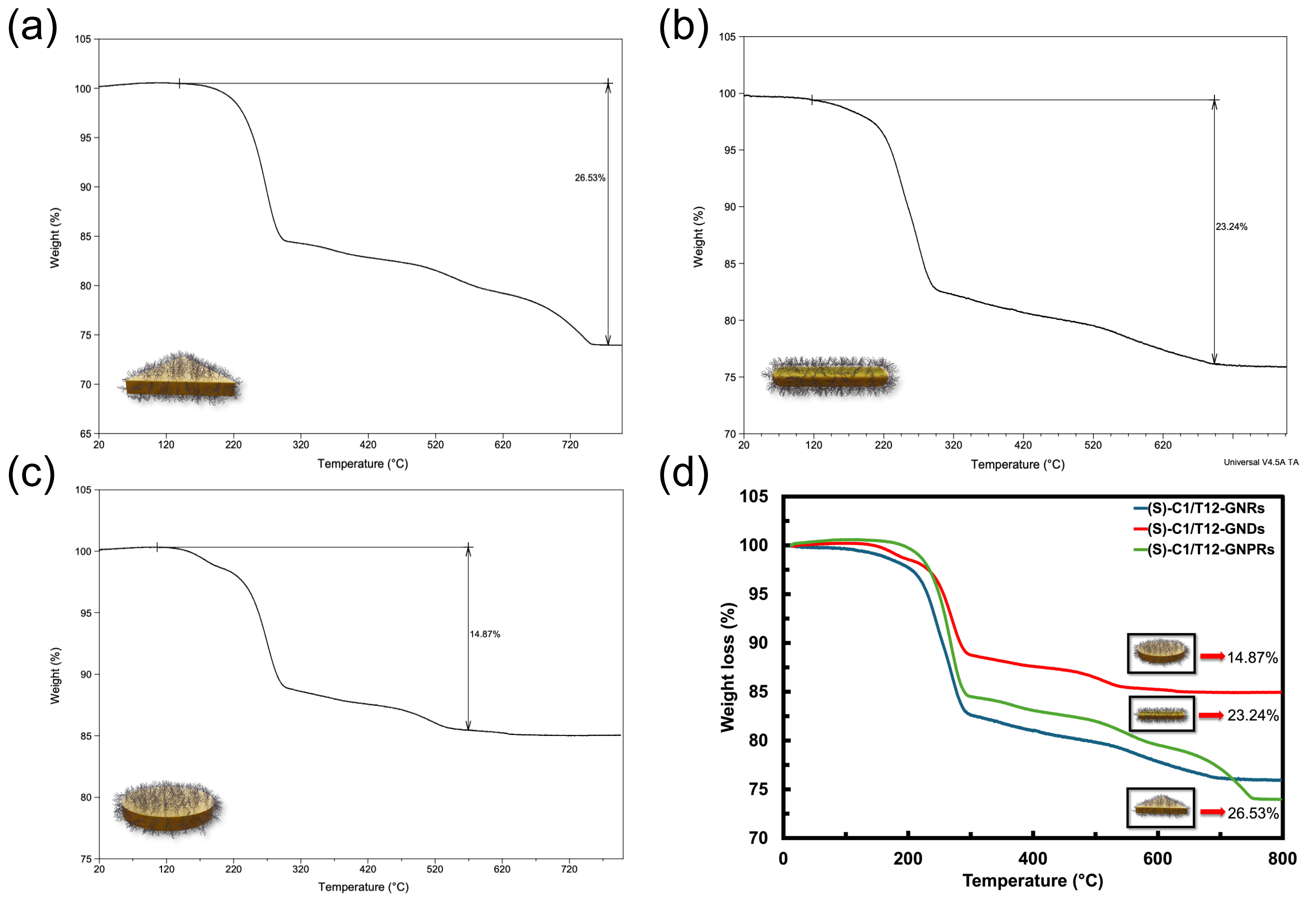

Figure S36. Thermogravimetric analysis of (*S*)-C1/T12-S–capped gold nanoshapes: (a-c) TGA profiles for GNPRs, GNRs, and GNDs, respectively. (d) Summary of mass-loss values obtained from TGA, showing 14.87%, 23.24%, and 26.53% for GNDs, GNRs, and GNPRs respectively, which reflect the relative ligand coverage (wt.%) on each gold nanoshapes.

Considering the real shapes of the GNRs, GNPRs, and GNDs as seen by TEM, the idealized shapes employed to estimate the ligand coverage in wt.% overestimate the number of gold atoms and underestimate the number of (*S*)-C1/T12-S− ligands as shown in Figure S36. The calculations followed the approach reported previously.[8b,12a] For the nanoshapes we assumed that the idealized shape over-estimates the number of gold atoms by 30% for the GNPRs, 20% for the GNRs, and 25% for the GNDs and underestimates the number of ligands by 20% for the GNPRs, 40% for the GNRs, and 25% for the GNDs. These estimates were based on the overall degree of curvature any given nanoshape (largest for the GNRs) as well as interdigitation and intercalation of the ligands on curved surfaces (Figure S36c). Intercalation, in line with data presented by Chen et al.[15a] for disc-like molecules such as **1** (likely also (*S*)-C1/T12-S−), is assumed since these molecules tend to form clusters (based on XRD data) due to $\pi - \pi$ interactions.

Table S1. Measured weight loss determined by thermogravimetric analysis (TGA) in comparison to the calculated ligand coverage (wt.%) of the (*S*)-C1/T12-S− ligands on the different nanoshapes. Calculated wt.% continue to underestimate the number of ligands but in a somewhat consistent fashion (3.6 – 5.4 wt.%) but both datasets show the same consistent trends, i.e. the shape with the largest surface to volume ratio (the GNPRs) shows the highest wt.% ligand coverage and the shape with the smallest surface to volume ratio the lowest wt.% (the GNDs).

Nanoshape	wt.%$_{Ligand}$ (estimated)	wt.%$_{Ligand}$ (TGA)
**(*S*)-C1/T12-S−GNR**	18.7	23.2
**(S)-C1/T12-S−GNPR**	21.1	26.5
**(S)-C1/T12-S−GND**	11.3	14.9

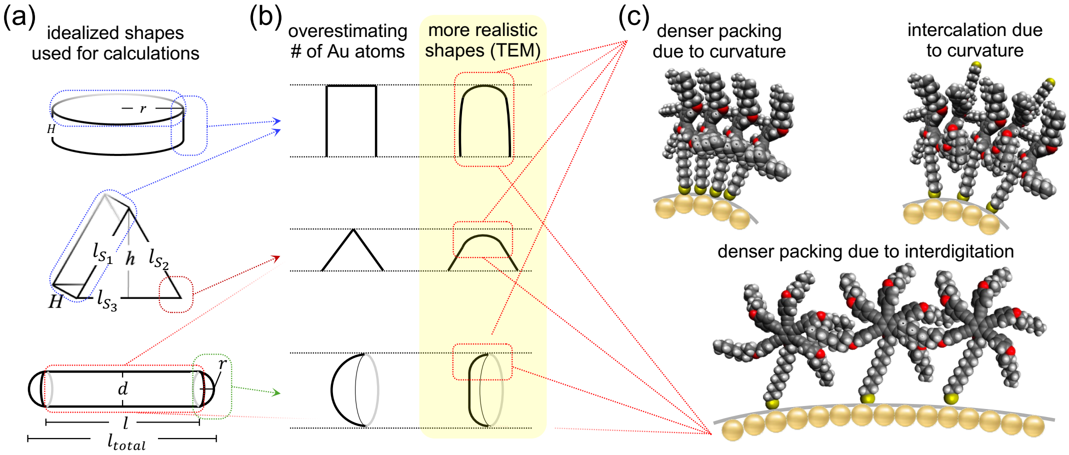

Figure S37. Comparison of idealized and real nanoshapes: (a) idealized shapes, (b) locations where the idealized shapes overestimate the number of gold atoms, and (c) ways how curbed shapes lead to underestimation of the number of (*S*)-C1/T12-S− ligands.

S7. N_D*-LC phase induced by chiral free ligands

S7.1. Polarized optical microscopy images of induced N_D*-LC phase

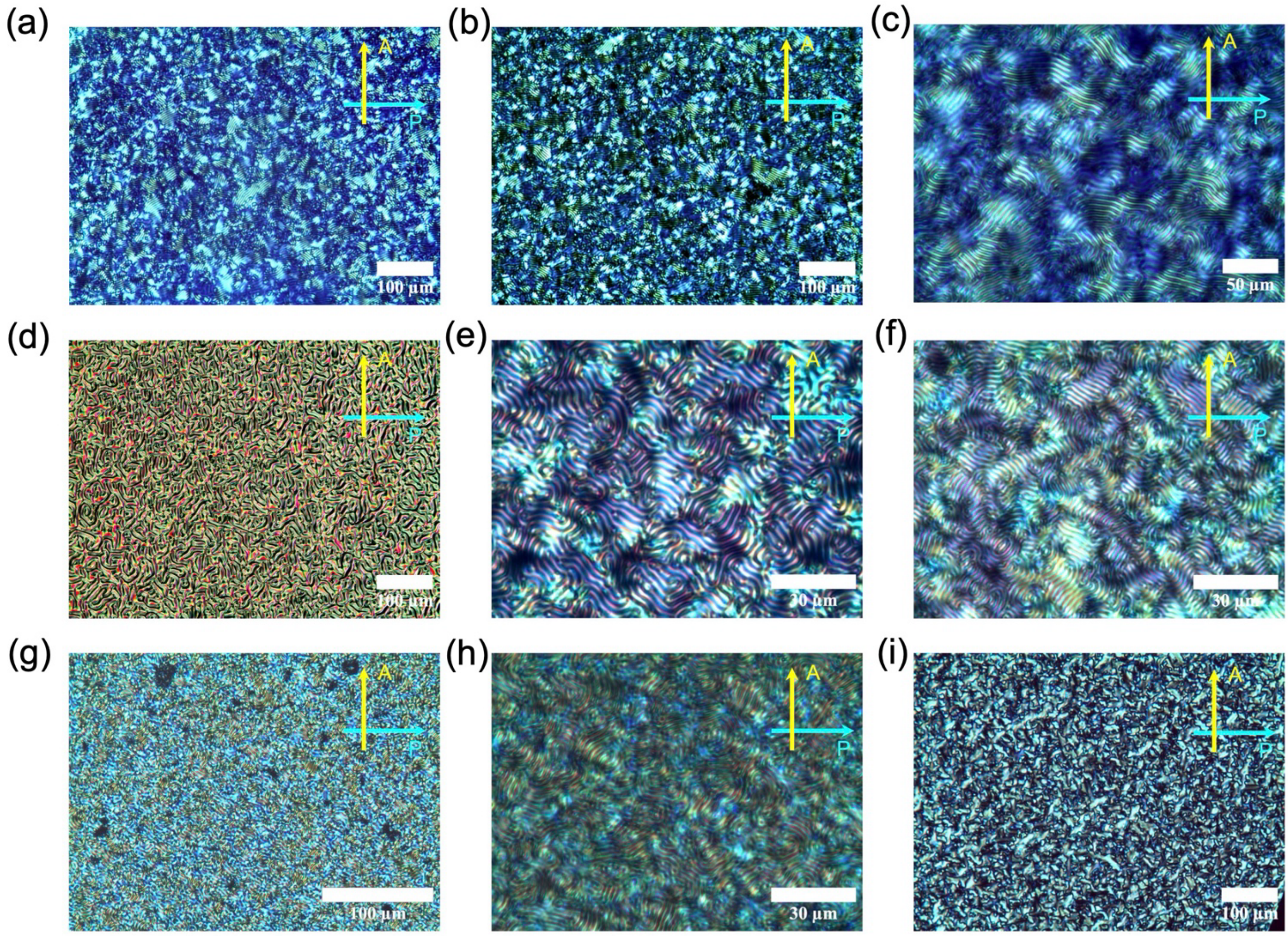

Figure S38. Polarized optical micrographs (crossed polarizers) of the N_D*-LC at $T - T_{NI}$ = −5 °C after doping with: (a-c) 30.0 wt.% (*R*)-C3/T8-SAc, (d) 1.0 wt.% (*S*)-C1/T8-SAc, (e) 2.0 wt.% (*S*)-C1/T8-SAc, (f) 3.0 wt.% (*S*)-C1/T8-SAc, (g) 1.0 wt.% (*S*)-C1/T12-SAc (h) 2.5 wt.% (*S*)-C1/T12-SAc, (i) 3.5 wt.% (*S*)-C1/T12-SAc. All mesomorphic behavior was investigated through a cooling scan and in sandwiched glass cells treated to favor homeotropic anchoring conditions with a cell gap = 10.0 µm.

S7.2 Twist direction of induced N_D*-LC phase depending on position and configuration of chiral center for the chiral ligands (Gray-McDonnell Rules)

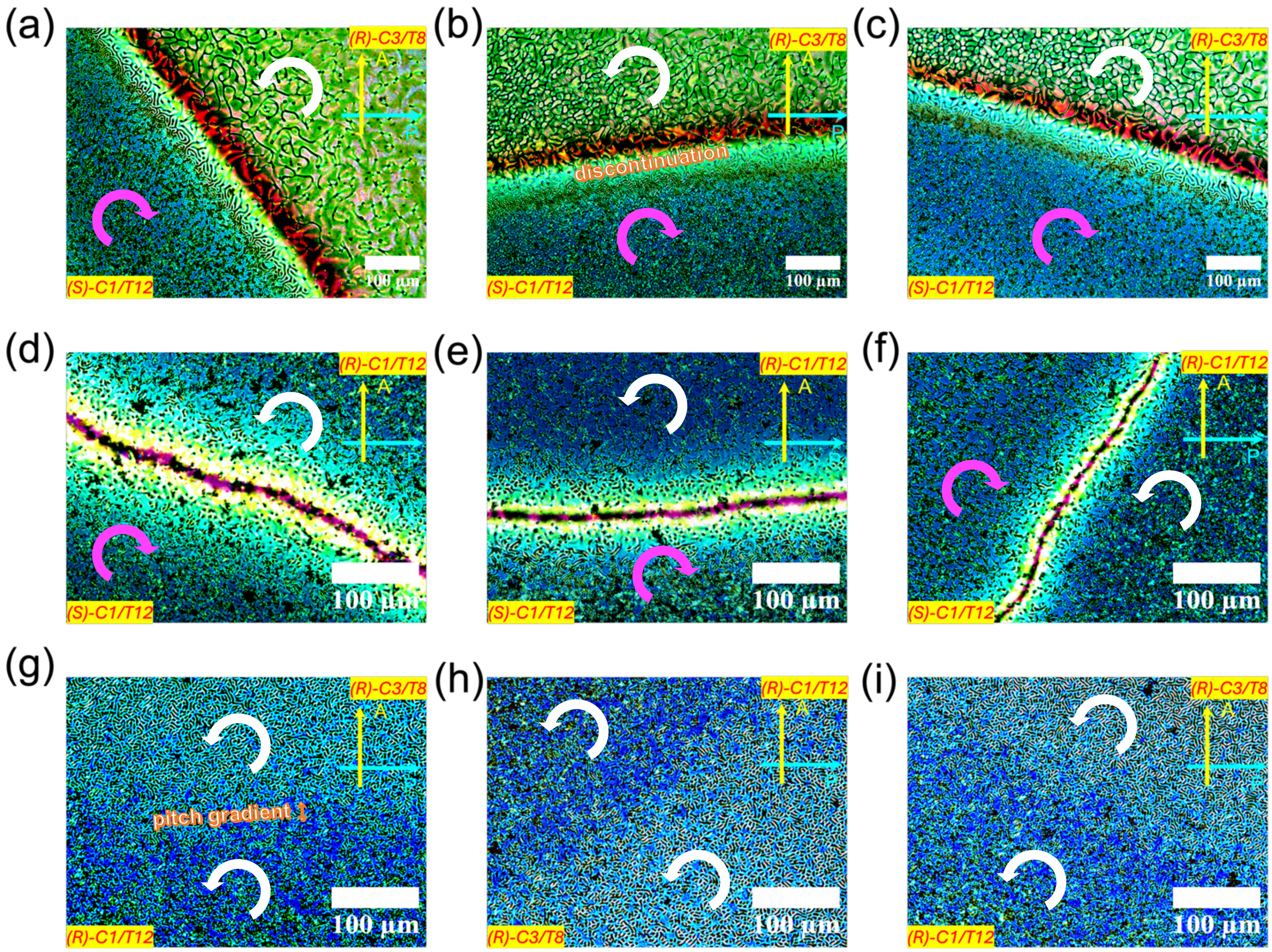

Figure S39. Polarized optical micrographs (crossed polarizers) of the miscibility test (contact preparations) between the N_D*-LC induced by: (a-c) 20.0 wt.% (*R*)-C3/T8-SAc and 2.5 wt.% (*S*)-C1/T12-SAc, (d-f) 2.5 wt.% (*S*)-C1/T12-SAc and 2.5 wt.% (*R*)-C1/T12-SAc, (g-i) 2.5 wt.% (*R*)-C1/T12-SAc and 20.0 wt.% (*R*)-C3/T8-SAc. All mesomorphic behavior was investigated through a cooling scan and in sandwiched glass cells treated to favor homeotropic anchoring conditions with a cell gap = 10.0 µm. Curly arrows show the handedness.

S7.3 Thin film induced circular dichroism (ICD) spectropolarimetry of induced N_D*-LC phase and solution CD

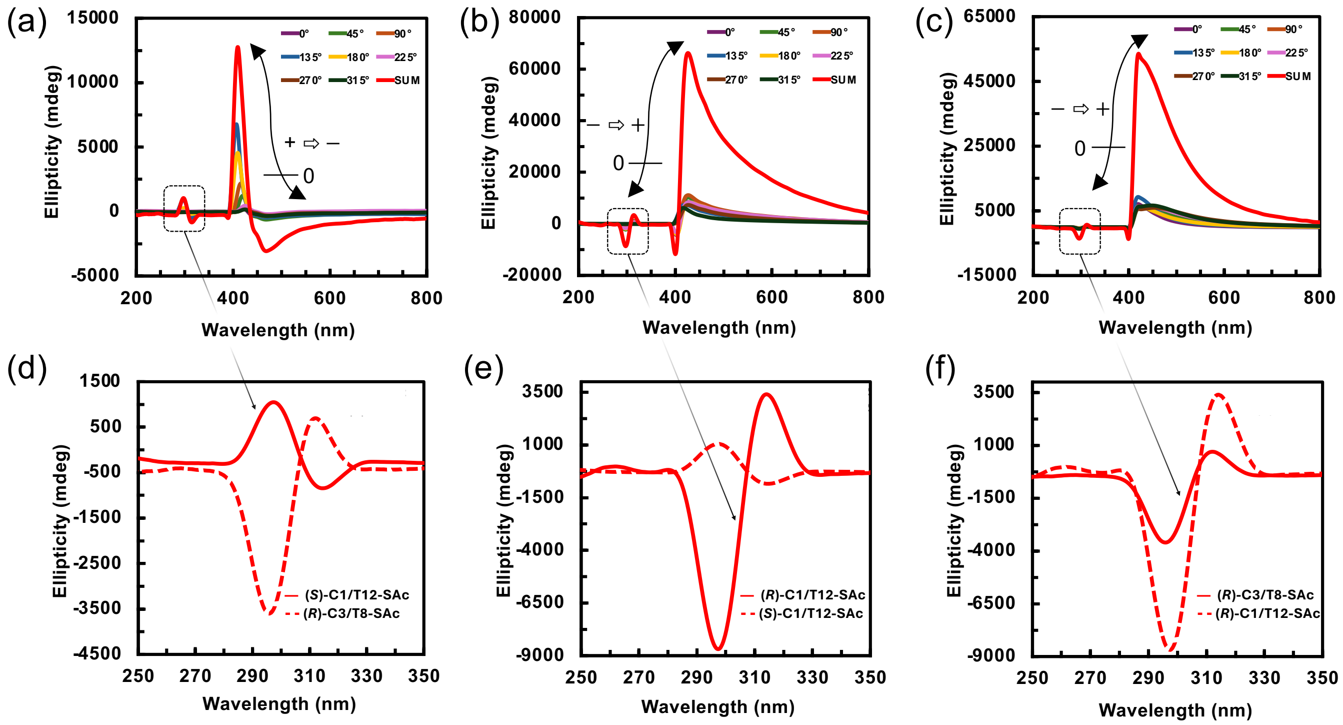

Figure S40. (a-c) Thin-film induced circular dichroism (ICD) spectra of N_D*-LC induced by taken at *T* = 25 °C between untreated quartz substrates with a cell gap = 10.0 µm: (a) 5.0 wt.% (*S*)-C1/T12-SAc, (b) 5.0 wt.% (*R*)-C1/T12-SAc, and (c) 35.0 wt.% (*R*)-C3/T8-SAc in **1**. The spectra recorded at 45° sample rotation angles as indicated in the legends; red or blue sum-CD signal datasets cancel out linear dichroism and birefringence. The change of sign of the induced CD signals between λ = 400 – 500 nm is consistent with the handedness differences observed in the contact preparations (inserts in Figures (a-c). (d-f) Zoom into the ICD spectra (spectral range from λ = 250 – 350 nm) for: (d) (*S*)-C1/T12-SAc in comparison to (*R*)-C3/T8-SAc, (e) (*R*)-C1/T12-SAc in comparison to (*S*)-C1/T12-SAc, and (f) (*R*)-C1/T12-SAc in comparison to (*R*)-C3/T8-SAc.

S8. Polarized optical microscopy images of the N_D*-LC phase obtained by admixing gold nanoshapes

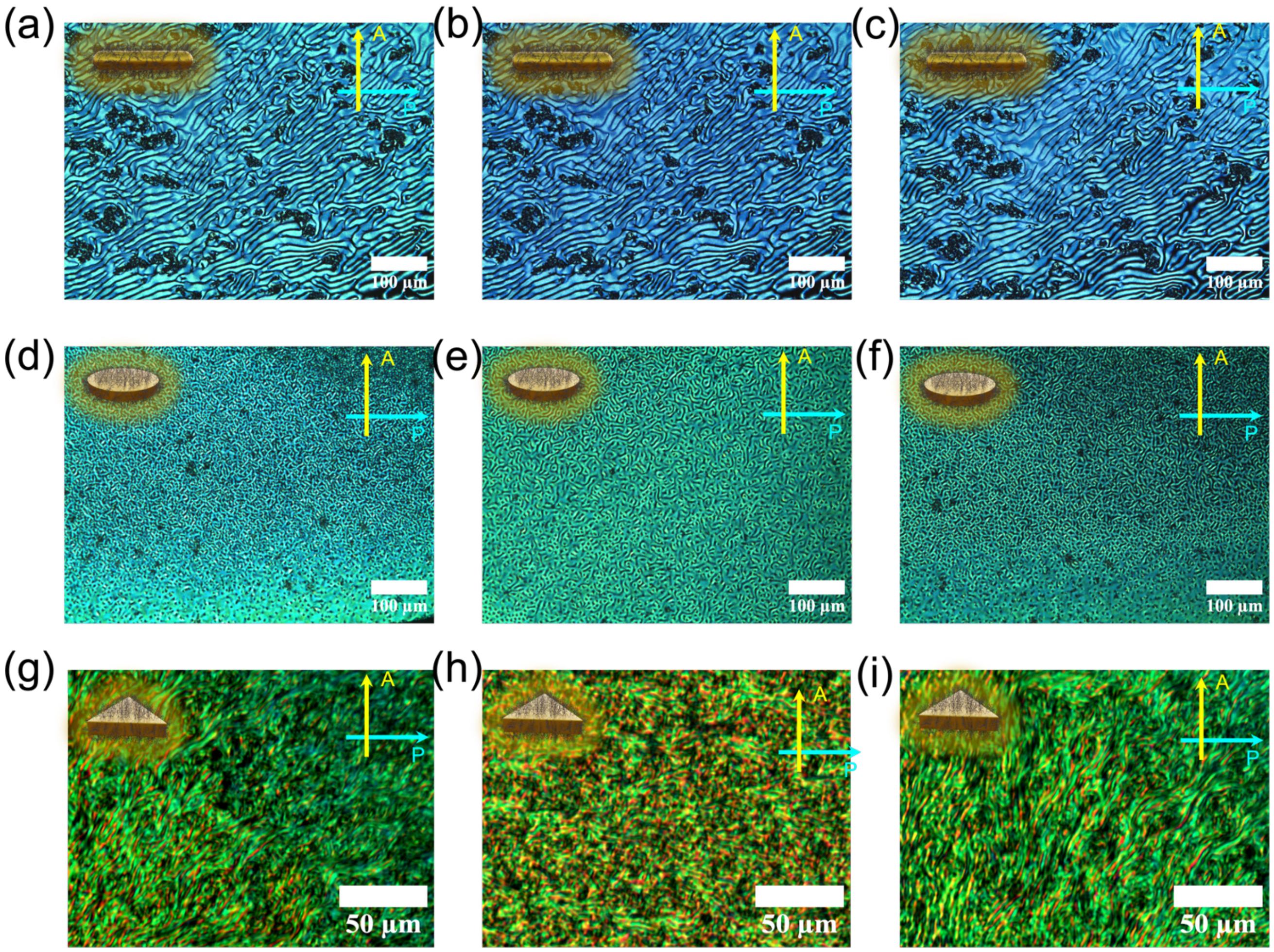

Figure S41. Polarized optical micrographs (crossed polarizers) of the N_D*-LC at $T - T_{NI}$ = −5 °C after doping with: (a-c) 1.0 wt.% (*S*)-C1/T12-S−GNRs, (d-f) 1.5 wt.% (*S*)-C1/T12-S−GNDs, (g-i) 1.5 wt.% (*S*)-C1/T12-S−GNPRs. All mesomorphic behavior was investigated through a cooling scan and in sandwiched glass cells treated to favor homeotropic anchoring conditions with a cell gap = 10.0 µm.

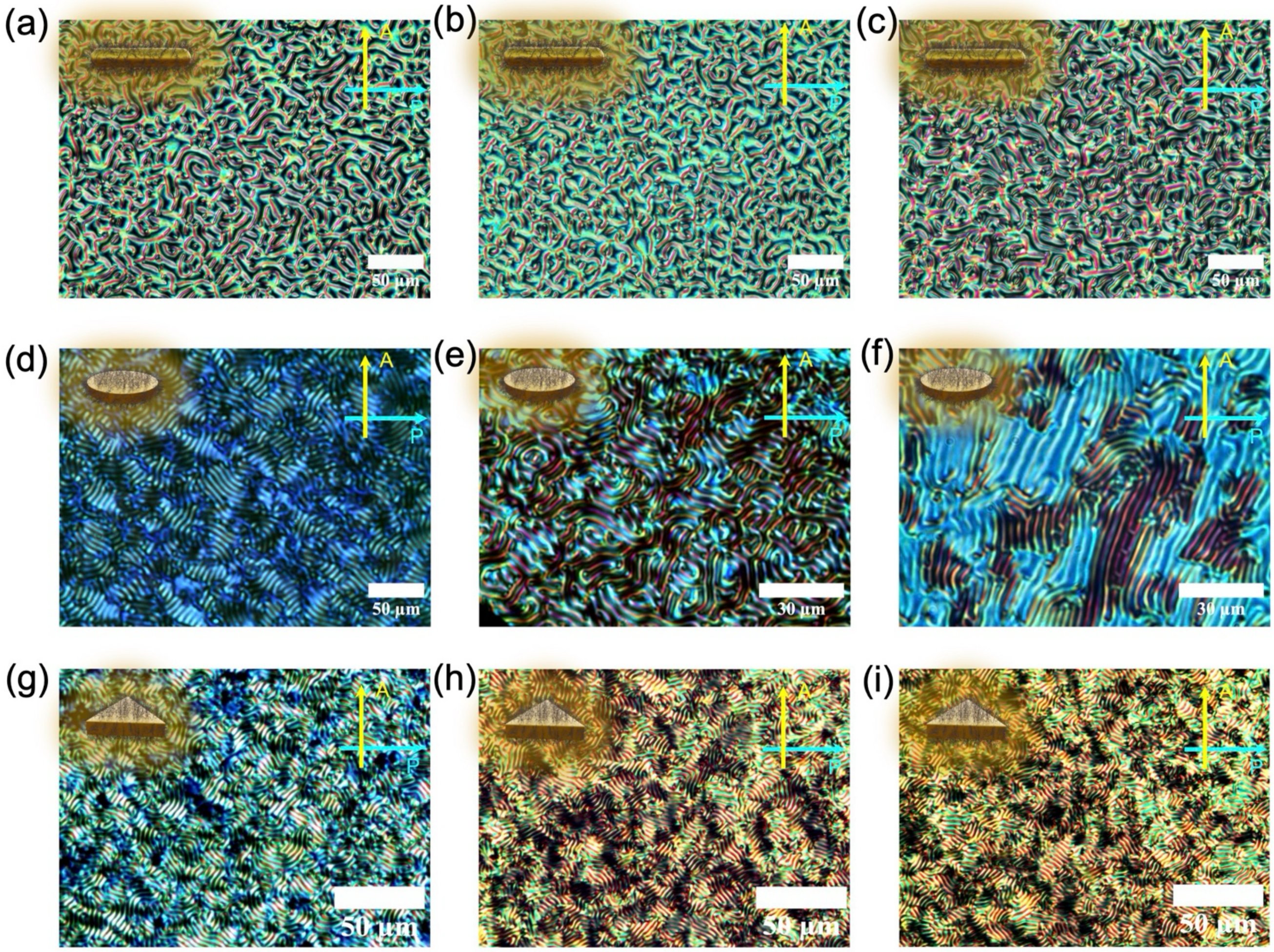

Figure S42. Polarized optical micrographs (crossed polarizers) of the N_D*-LC at $T - T_{NI}$ = −5 °C after doping with: (a-c) 2.0 wt.% (*S*)-C1/T12-S−GNRs, (d-f) 2.0 wt.% (*S*)-C1/T12-S−-GNDs, (g-i) 2.0 wt.% (*S*)-C1/T12-S−GNPRs. All mesomorphic behavior was investigated through a cooling scan and in sandwiched glass cells treated to favor homeotropic anchoring conditions with a cell gap = 10.0 µm.

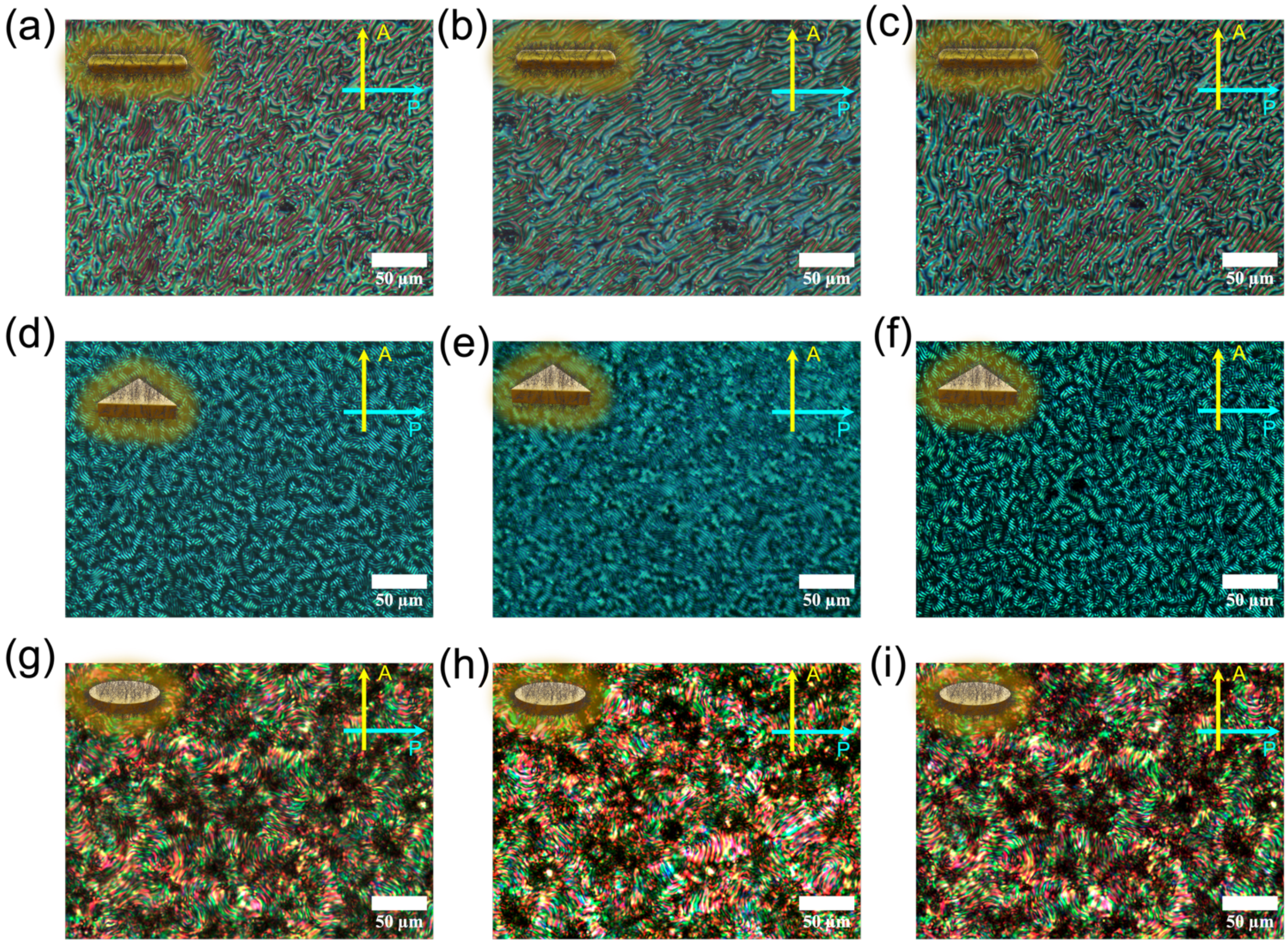

Figure S43. Polarized optical micrographs (crossed polarizers) of the N_D*-LC at $T - T_{NI} = -5$ °C after doping with: (a-c) 2.5 wt.% (*S*)-C1/T12-S−GNRs, (d-f) 2.5 wt.% (*S*)-C1/T12-S−GNDs, (g-i) 2.5 wt.% (*S*)-C1/T12-S−GNPRs. All mesomorphic behavior was investigated through a cooling scan and in sandwiched glass cells treated to favor homeotropic anchoring conditions with a cell gap = 10.0 µm.

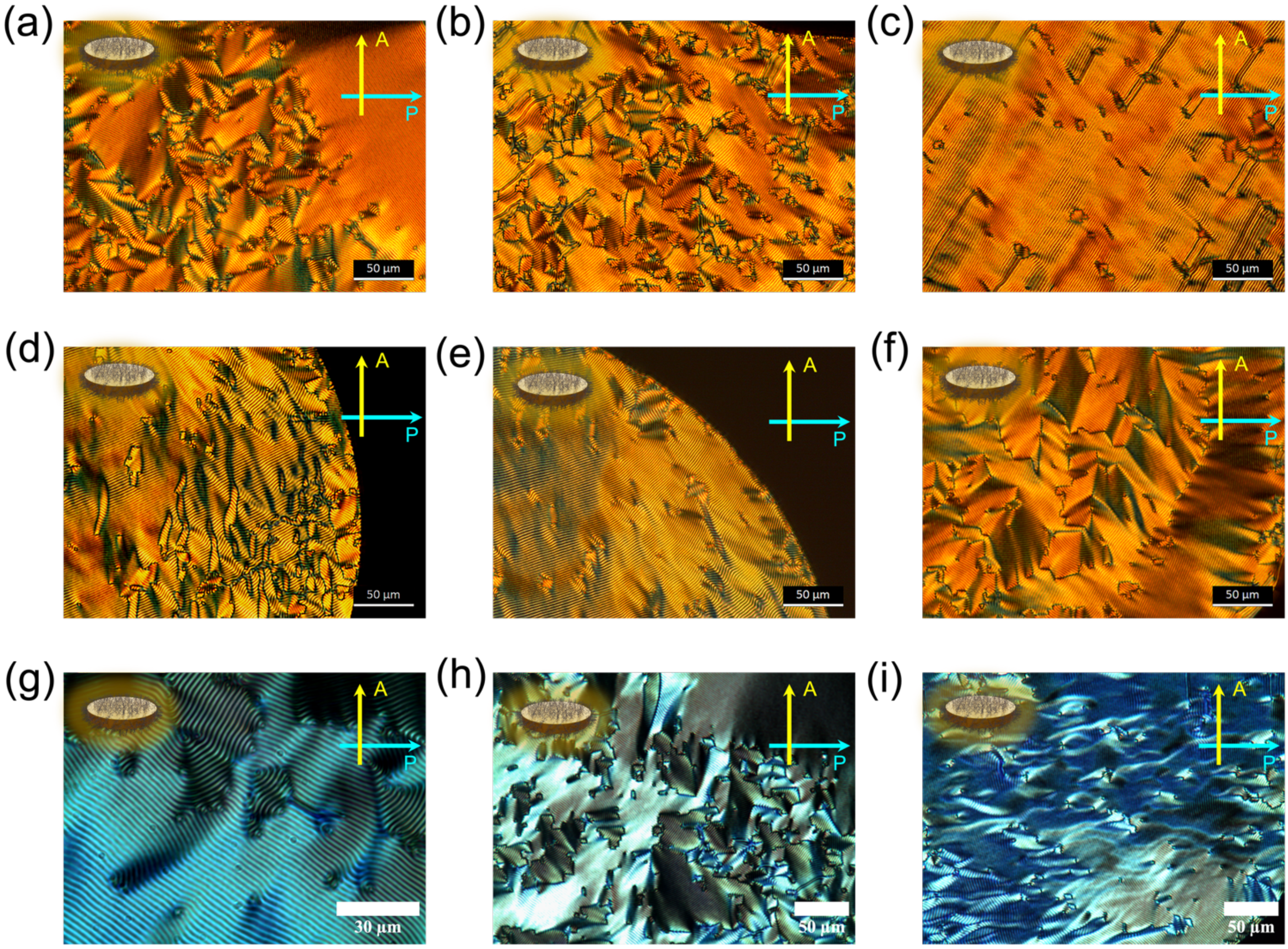

Figure S44. Polarized optical micrographs (crossed polarizers) of the N_D*-LC at $T - T_{NI}$ = −5 °C after doping with: (a-i) 3.0 wt.% (*S*)-C1/T12-S−GNDs. All mesomorphic behavior was investigated through a cooling scan and in sandwiched glass cells treated to favor homeotropic anchoring conditions with a cell gap = 10.0 µm.

S9. S^{XN} values for ST, RP, and RH shapes

Table S2. Values of S^{XN} with respect to the nematogen IPR$_{2D}$ computed as RP (Reuleaux pentagon), RH (Reuleaux heptagon), ST (semicircle with adjoined right isosceles triangle), and a rigid molecular envelope; some values appear the same due to rounding.

Nanoshape	S^{XN}_{ST}	S^{XN}_{RP}	S^{XN}_{RH}	S^{XN}_{mol}
**GNR**	0.08	0.08	0.08	0.09
**GNPR**	0.14	0.13	0.13	0.16
**GND**	0.77	2.24	4.57	0.50

S10. Plots of inverse pitch vs. mole fraction: calculation of $|\beta_{mol}|$

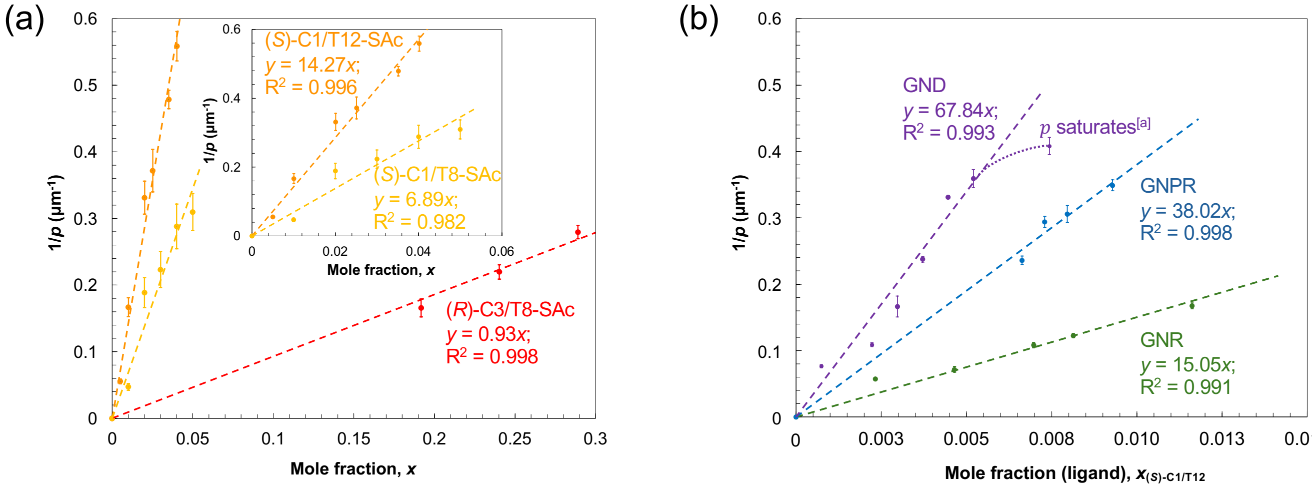

Figure S45. Plots of the inverse pitch, $1/p$ (measured by POM) vs. the mole fraction of: (a) the chiral free thiols (*R*)-C3/T8-SAc, (*S*)-C1/T8-SAc, and (*S*)-C1/T12-SAc and (b) the three nanoshapes capped with (*S*)-C1/T12-S−; [a] only the most potent GNDs show a pitch saturation at the highest concentration as previously observed for the GNR$_{MAR}$ in 5CB[8b].

S11. Quantification of chirality transfer by geometrical chirality model

S11.1 Atomistic and coarse-grained ligand structures

To evaluate whether the spatial arrangement of chiral ligands on the nanoshape surface contributes to a non-linear amplification of chirality and correspondingly to an enhanced helical twisting power an energy-minimized molecular model was constructed for the ligands (*R*)-C3/T8-SH, (*S*)-C1/T8-SH, and (*S*)-C1/T12-SH. All structures were built using the public domain software "Avogadro" and minimized (in vacuum) the structure of a united-atom molecule of the three ligands. The resulting minimized structures are shown below.

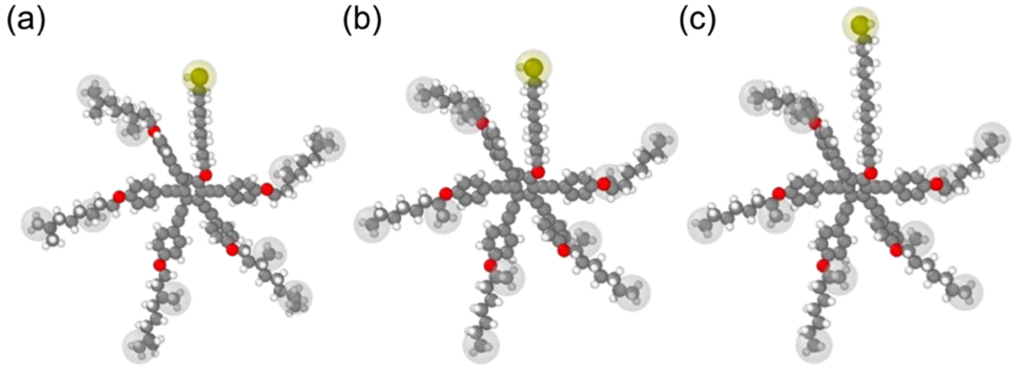

Figure S46. Energy-minimized structure of: (a) (*R*)-C3/T8-SH, (b) (*S*)-C1/T8-SH, and (c) (*S*)-C1/T12-SH; beads are solid for atomistic model, while translucent for coarse-grained one (identical CC solutions for —SH or —SAc).

S11.2 Gold nanoshapes for ligand-shell

The gold nanoshapes studied are GNRs (average total length l_{total} = 95.9 nm, average width = 29.3 nm), GNDs (average diameter = d = 79.5 nm, average height H = 29.9 nm) and triangular nanoprisms (average side length l_s = 50.2 nm, average height H = 26.5 nm). These nanoshapes have been used to model the positions of the ligands but only kept their centers of mass when computing chirality index.

S11.3 Chiral ligands

The coarse-grained version of the ligands was used, which were composed by 11 beads. A ligand in two distinct configurations has been chosen to consider, obtained by rotating the molecule along its principal axis of 180°. Moreover, it has been decided to apply a tilt angle (with respect to main molecular axis) of either 0° or 60°. These four configurations are shown in the figure below.

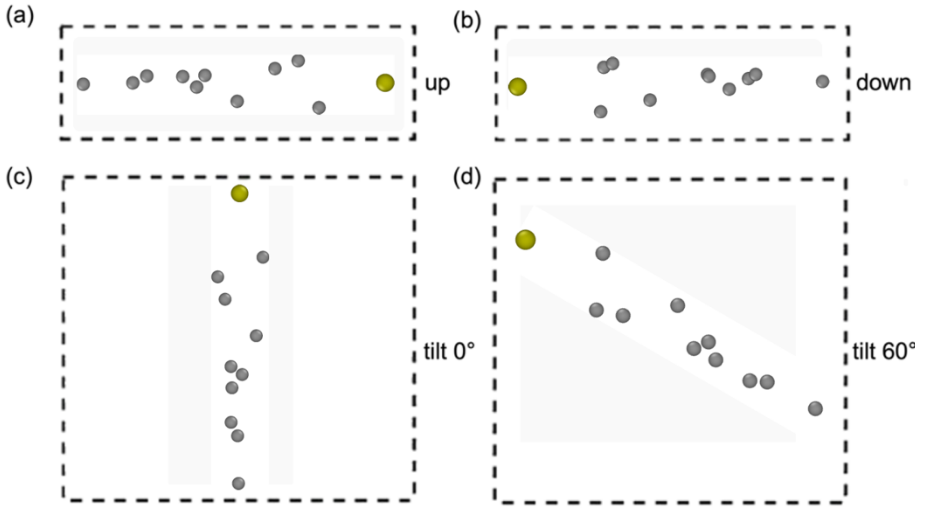

Figure S47. The four different configurations of coarse-grained ligands: (a) in up direction, (b) in down direction, (c) tilted in 0° angle, and (d) tilted in 60° with respect to principal molecular axis.

S11.4 Ligand decorated nanoshapes[8b,12a]

For each ligand and nanoshape, a set of 22 sterically permissible binding sites on the nanoparticle surface was considered. Combinatorial arrangements comprising 20 ligands were subsequently constructed from these sites, resulting in 231 possible configurations for each ligand-nanoshape system.

The following five distinct systems were selected for analysis:

- ligands with “up” configuration, tilt angle of 0°
- ligands with “down” configuration, tilt angle of 0°
- ligands with “up” configuration, tilt angle of 60°
- ligands with “down” configuration, tilt angle of 60°
- ligands randomly chosen from previous systems

The figure below shows as an example the nanoparticles decorated with (*S*)-C1/T12 in mixed configuration.

(a)
(b)
(c)

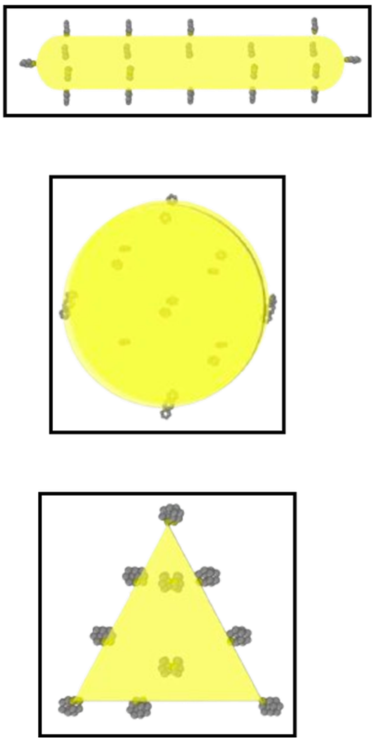

Figure S48. Representative images of (*S*)-C1/T12-S−capped gold nanoshapes: (a) GNRs, (b) GNDs, (c) GNPRs.

S11.5 Values of IPR^{X}_{2D}, S^{XN}_{mol}, and $|G^{a}_{oa,max}S^{XN}_{mol}|$ for chiral organic solutes and nanoshapes assuming rigid molecular envelope

Table S3. Values of IPR^{X}_{2D}, S^{XN}_{mol}, and $|G^{a}_{oa,max}S^{XN}_{mol}|$ for the chiral ligands with respect to the nematogen IPR_{2D} computed as molecular envelope with IPR^{N}_{2D} = 14.74 as outlined in Section S10.5.

Chiral Solute X	IPR^{X}_{2D}	S^{XN}_{mol}	$\lvert G^{a}_{oa,max}S^{XN}_{mol}\rvert$
**(*R*)-C3/T8-SAc**	13.75	1.01	0.79
**(*S*)-C1/T8-SAc**	14.20	1.84	1.82
**(*R*)-C1/T128-SAc**	14.56	5.59	6.71
**(*S*)-C1/T12-SAc**	14.56	5.59	6.71
**(*S*)-C1/T12-S-GNR**	25.94	0.09	886.50
**(*S*)-C1/T12-S-GNPR**	20.79	0.16	1311.04
**(*S*)-C1/T12-S-GND**	12.57	0.50	1334.50

S11.6 Plots of $|G^{a}_{oa,max}S^{XN}|$ or $|G^{a}_{oa,max}S^{XN}_{mol}|$ vs. $|\beta_{mol}|$ assuming RH and RP shapes as well as rigid molecular envelopes of 1

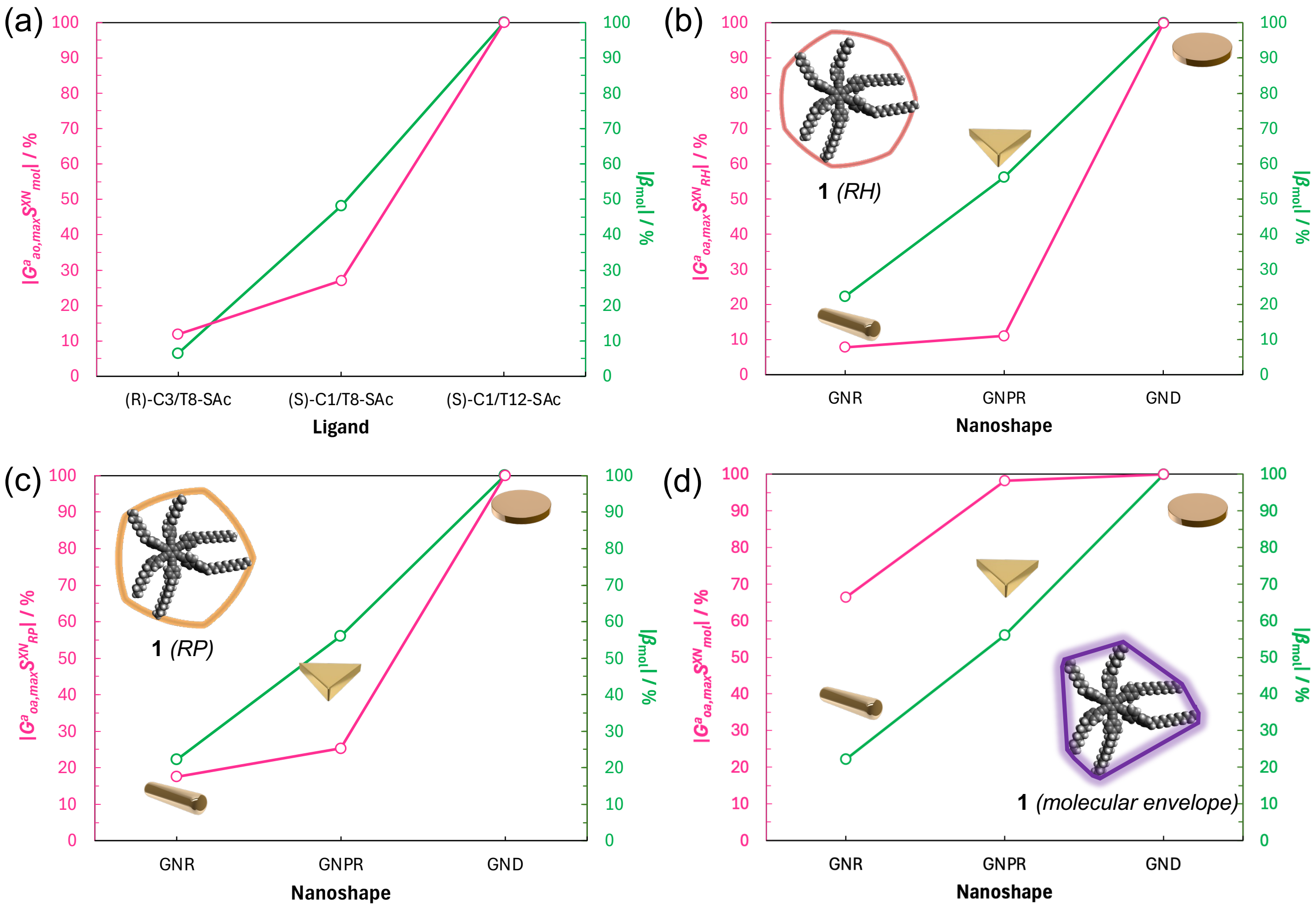

Figure S49. Trends of $|G^{a}_{oa,max}S^{XN}|$ or $|G^{a}_{oa,max}S^{XN}_{mol}|$ vs. $|\beta_{mol}|$ assuming: (a) a rigid molecular envelope for **1** and the chiral ligands, (b) an RH shape of **1**, (c) an RP shape of **1**, and (d) a rigid molecular envelope of **1**; all showing the correct overall correlations.

S12. Variation of GND height (i.e., aspect ratio, *AR*)

S12.1 Synthesis and characterization of GND with *AR* ~ 5.4 (GND$_{AR\sim5.4}$)

GNDs with a larger aspect ratio of *AR* ~ 5.4 were synthesized following a modified three-step seed-mediated growth protocol adapted from Wang et al. [18c,43] involving: (i) the formation of triangular gold nanoprisms, (ii) an overgrowth step into hexagonal nanoplates, and (iii) an anisotropic oxidative etching to yield GNDs.

Synthesis of precursor GNPRs: To prepare the seed solution, in a 50 mL Erlenmeyer flask, 36.0 mL of deionized (DI) water was stirred at 400 rpm at room temperature. Subsequently, 1.0 mL of 0.01M trisodium citrate and 1.0 mL of 0.01 M $HAuCl_4$ were added sequentially under continuous stirring. After 2 min, 1.0 mL of freshly prepared ice-cold 0.1M $NaBH_4$ was rapidly injected under vigorous stirring (1,200 rpm), resulting in the formation of a brown colored seed solution. The seed solution was aged for 7 h at room temperature to ensure complete hydrolysis of residual $NaBH_4$ prior to use.

Preparation of three growth solutions (Growth-I and Growth-II solutions): In two separate 20 mL scintillation vials, 9.0 mL of 0.05M CTAB and 0.25 mL of 0.01M $HAuCl_4$ were mixed and stirred at 30 °C (250 rpm) for 2 min. Then, 0.05 mL of 0.1M NaOH and 0.05 mL of 0.01M KI were added sequentially, followed by 0.05 mL of 0.1M ascorbic acid. Upon addition of ascorbic acid, the solution became colorless, indicating reduction of Au(III) to Au(I). Stirring was then stopped.

Growth-III Solution: In a 125 mL Erlenmeyer flask, 90.0 mL of 0.05M CTAB and 2.5 mL of 0.01M $HAuCl_4$ were mixed and stirred at 30 °C (300 rpm) for 2 min. Subsequently, 0.5 mL of 0.1M NaOH and 0.5 mL of 0.01 M KI were added, followed by 0.5 mL of 0.1M ascorbic acid. The solution turned colorless, and stirring was stopped.

Preparation of gold nanoprism solution and purification: After aging, 1.0 mL of the seed solution was injected into the Growth-I solution and gently mixed for less than 3 s, producing a faint pink color. Immediately, 1.0 mL of this intermediate solution was transferred to Growth-II under rapid mixing (< 3 s). The entire resulting mixture was then added to the Growth-III solution and gently shaken for ~5 s. A characteristic color transition (colorless → pink → purple → magenta) indicated the initiation of triangular nanoprism formation.

The reaction mixture was transferred to a 100 mL graduated cylinder and left undisturbed for 24 h to allow precipitation driven by surfactant-induced depletion forces. The supernatant was carefully decanted without disturbing the precipitate. The GNPRs were redispersed in 40.0 mL DI water (green-colored dispersion) and purified via centrifugation (12,000 rpm, 10 min) twice. The purified nanoprisms were concentrated into 2.0 mL DI water. The stock solution concentration was adjusted to an absorbance of 3.0 by using Vis-NIR spectroscopy for subsequent use as seeds in the overgrowth step.

Synthesis of overgrown hexagonal nanoplates: In a 4.0 mL vial, 0.25 mL of 0.1M CTAB was heated to 30 °C. Then, 0.02 mL of 0.01M $HAuCl_4$ was added and stirred at 300 rpm for 2 min. Next, 0.01 mL of 0.1M ascorbic acid was added, resulting in a color change from yellow to colorless. Stirring was stopped, and 1.72 mL DI water was added with gentle shaking for 10 s. Finally, 0.5 mL of the GNPR stock solution (Abs = 3.0) was added. The reaction mixture was left undisturbed at 30 °C for 13 h to yield overgrown hexagonal nanoplates.

Synthesis of gold nanodiscs (GND$_{AR\sim5.4}$): Facet-selective oxidative etching of the hexagonal nanoplates was performed using H_2O_2 and HCl under controlled conditions. 0.035 mL of 6% H_2O_2 and 0.030 mL of 1.0 M HCl were added, and the reaction was performed at 40 °C with stirring (250 rpm) for 1 h. Prolonged reaction times led to overoxidation and loss of anisotropy; therefore, strict control of reaction duration was essential.

Purification of GNDs$_{AR\sim5.4}$:
Following oxidation, the reaction mixture was immediately purified to remove residual oxidants. Centrifugation was performed at 10,000 rpm for 12 min (three cycles). The supernatant was discarded, and the purified gold nanodiscs were redispersed in 1.0 mL DI water to obtain the final stock solution. The resulting GNDs were subsequently used for ligand exchange with (*S*)-C1/T12-SH as described in Section S6 above.

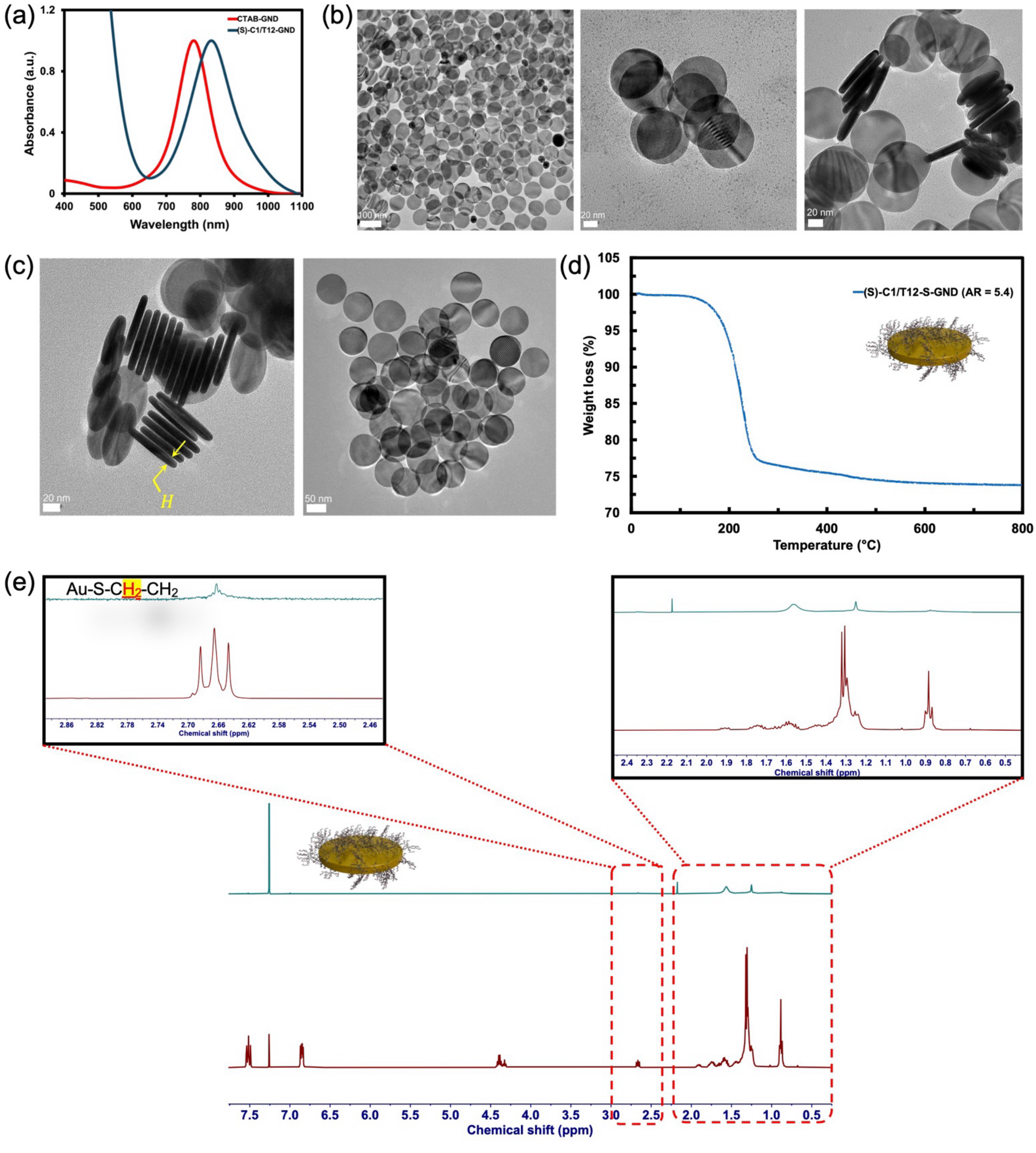

Figure S50. (a) Vis-NIR spectra before (CTAB-capped) and after surface functionalization of the GNDs (*AR* ~ 5.4) with the chiral ligand (*S*)-C1/T12-SH, exhibiting the characteristics red-shift of the plasmonic band (similar with the other nanoshapes) upon functionalization from λ_{max} = 788 nm (red) to λ_{max} = 833 nm (blue), (b) TEM images of the CTAB-coated GNDs with average diameter d = 73.6 nm and a height H = 13.6 nm (*AR* ~ 5.4), (c) TEM images of the final (*S*)-C1/T12-S−capped GNDs (images like the one on the left were used to measure the height of these GNDs, (d) Thermogravimetric analysis (TGA) of (*S*)-C1/T12-S–capped GNDs (*AR* ~ 5.4): obtained mass-loss by TGA wt.%$_{Ligand}$ = 22.9% which reflect the relative estimated ligand coverage on the GND surface (estimated: wt.%$_{Ligand}$ = 18.6%; see Section S6.3), and (e) comparative ^{1}H-NMR spectra of pristine (*S*)-C1/T12-SH (red) and (*S*)-C1/T12-S−capped GNDs (cyan), recorded in $CDCl_3$; (top left) expansion highlighting the methylene protons adjacent to the thiol group (-CH_2-S), which appear as a distinct triplet in the free ligand however appear broadened upon coordination on the surface of gold nanodiscs, (top right) spectral region 0.5 – 2.4 ppm for aliphatic region, showing the transition from well-resolved multiplets in the pristine ligand to broadened signals for GND-bound ligands.

S12.2 Polarized optical microscopy images of the N_D*-LC phase obtained by admixing GND with *AR* ~ 5.4

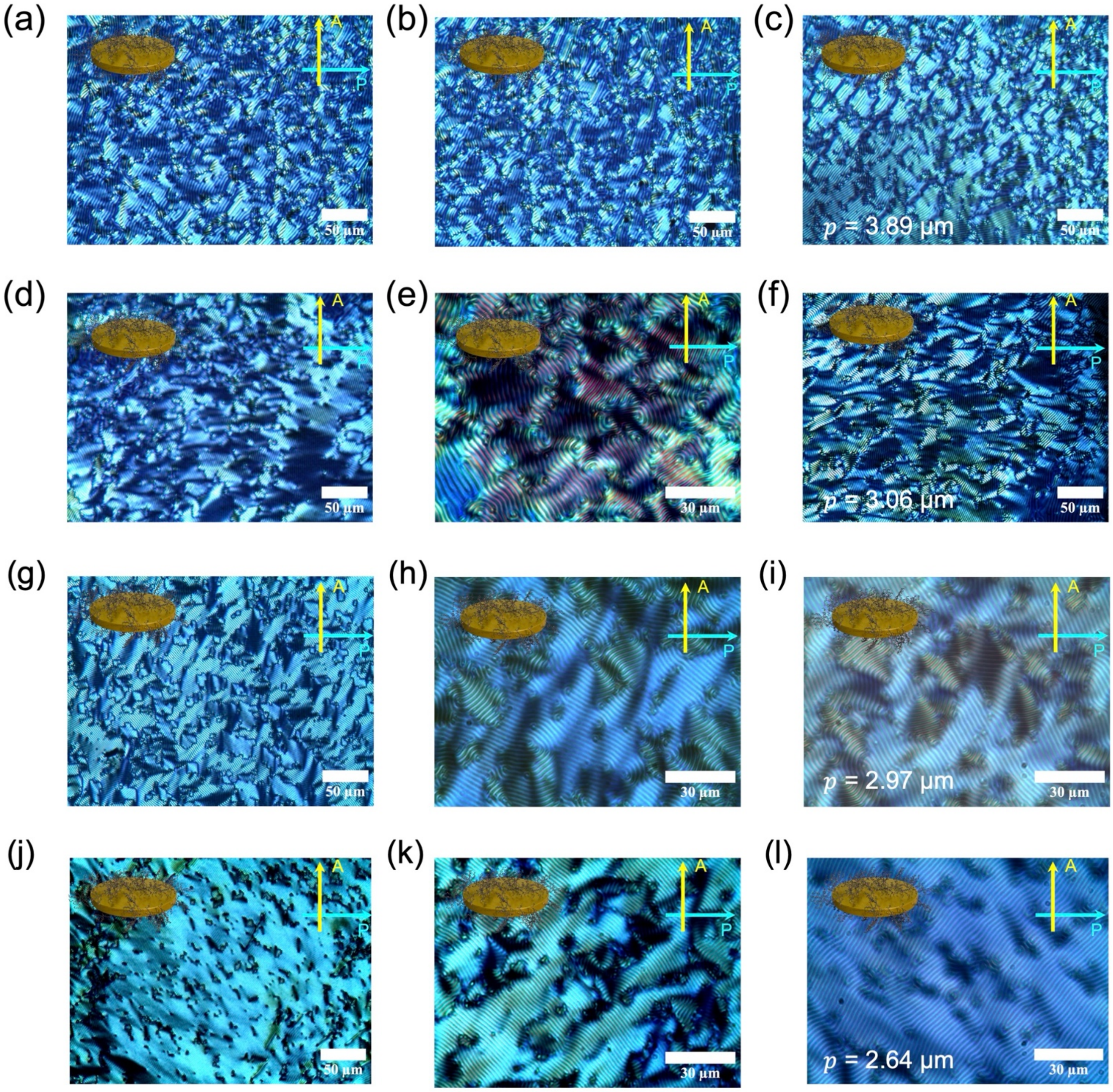

Figure S51. (a) Polarized optical micrographs (crossed polarizers) of the N_D*-LC at $T - T_{NI}$ = −5 °C after doping with: (a-c) 1.0 wt.% (*S*)-C1/T12-S−$GND_{AR\sim5.4}$, (d-f) 1.5 wt.% (*S*)-C1/T12-S−$GND_{AR\sim5.4}$, (g-i) 2.0 wt.% (*S*)-C1/T12-S−$GND_{AR\sim5.4}$, and (j-l) 2.5 wt.% (*S*)-C1/T12-S−$GND_{AR\sim5.4}$. All mesomorphic behavior was investigated through a cooling scan and in sandwiched glass cells treated to favor homeotropic anchoring conditions with a cell gap = 10.0 µm. Average helical pitch values measured by microscopy and used to calculate $|\beta_{mol}|$ are given in the images on the right, respectively.

S12.3 Plots of $1/p$ vs. mole fraction and $|G^{a}_{oa,max}S^{XN}|$ vs. $|\beta_{mol}|$ for GND$_{AR\sim 5.4}$ assuming a circular shape for 1

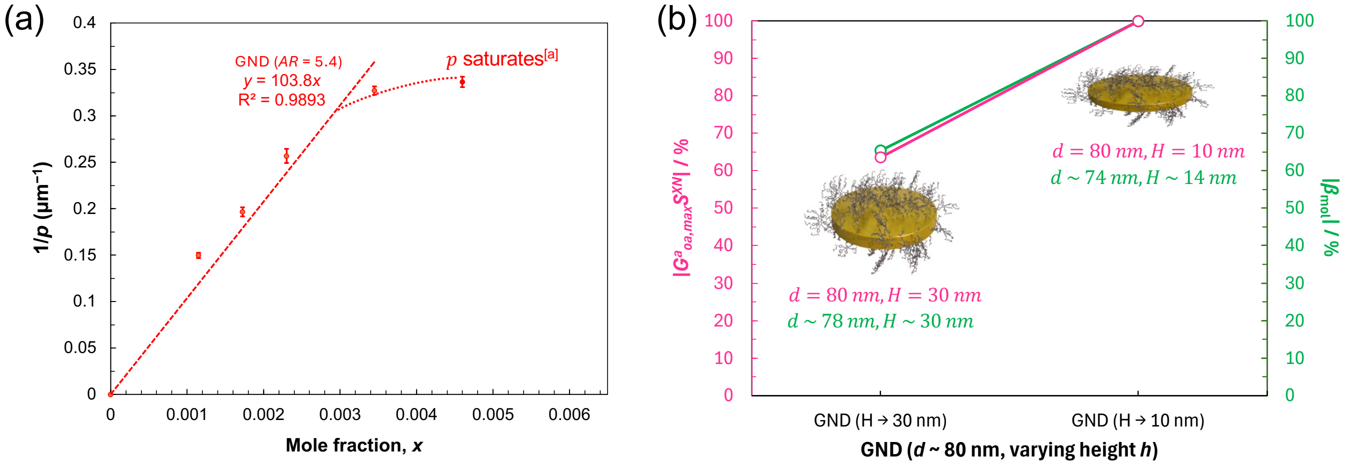

Figure S52. (a) Plot of the inverse pitch, $1/p$ (measured by POM) vs. the mole fraction of the GND$_{AR\sim 5.4}$ capped with (S)-C1/T12-S−; [a] as for the GNDs with lower AR (GND$_{AR\sim 2.6}$; see Figure S45b) a pitch saturation was observed for the highest concentration as previously observed for the GNR$_{MAR}$ in 5CB[8b]. (b) Trends of $|G^{a}_{oa,max}S^{XN}|$ vs. $|\beta_{mol}|$ for GNDs with an average diameter of $d\sim 80$ nm and a varying height H assuming a circular shape for **1** (i.e., S^{XN} = 1.0); values assumed for the calculation of $|G^{a}_{oa,max}S^{XN}|$ are given in pink; experimentally achieved dimensions close to those used for the calculations for the GNDs (determined by TEM) in green.

Author Contributions

G.A. with some assistance from N.M.K. and A.G. performed the synthesis and characterization of the organic compounds. G.A. completed the synthesis and characterization of the gold nanoprisms and gold nanorods; K.Z. performed and J.W. directed the synthesis of the gold nanodiscs. G.A. some assistance from A.S. did the POM studies. G.A.R.R and G.A. prepared the homeotropic cells for all the pitch measurements. G.A. performed all CD, NMR, and, with the help of G.A.R.R., the TEM studies. S.K.P. completed the X-ray diffraction experiments and analyzed these data. L.Q. and C.Z. performed all the chirality indicator calculations. T.H. proposed and directed the research as well as wrote the manuscript with major input from G.A. (specifically most of the SI section) and C.Z. G.A.R.R. provided the 3-D renderings of the N$_D^{(*)}$ phases.
